# The Wrong Place at the Wrong Time? Territorial Autonomy and Conflict During Regime Transitions

**DOI:** 10.1177/00104140231168365

**Published:** 2023-04-01

**Authors:** Andreas Juon, Daniel Bochsler

**Affiliations:** 1 27219 ETH Zurich, Switzerland; 2 47797 Central European University (CEU), Wien, Austria; 3 University of Belgrade, Serbia

**Keywords:** federalism, race, ethnicity and politics, civil war, ethnic conflict, democratization and regime change

## Abstract

This article evaluates how territorial autonomy affects ethnic mobilization and conflict during regime transitions. Previous research has highlighted its conflict-inducing role during prominent transition contexts. Alternatively, it has shown its pacifying role in the “average” case, without distinguishing transition periods from stable contexts. Addressing these gaps, we argue that the de-escalatory consequences of autonomy depend on critical stabilizing factors which are themselves “muted” during transitions. We test our expectations in a cross-national analysis, covering all regime transitions between 1946 and 2017. We also revisit the 1989 transition from Communism, focusing on the role of “inherited” autonomy in the post-communist successor states. This enables us to address concerns whereby autonomy is offered to ward off transitions or whereby transitions are themselves induced by mobilization. Our findings indicate that during transitions, territorial autonomy increases the likelihood of ethnic mobilization, government concessions in response, and violent escalation where these are not forthcoming.

## Introduction

Regime transitions are profoundly unstable times and often entail intense, sometimes violent, political conflict (Cederman et al., 2010; Hegre et al., 2001; Snyder, 2000). Does territorial autonomy reduce these risks, by reassuring diverse ethnic groups of their security in unstable times? Or is territorial autonomy a basis for ethno-nationalist mobilization, which group leaders might opportunistically exploit while the new regime is still unconsolidated (Cornell, 2002; Hale, 2004; Saideman et al., 2002)? A recent surge of conflicts in autonomous regions in democratizing or newly independent states underlines the importance of these questions.

In spite of these questions’ profound implications, previous research faces difficulties addressing them. A first strand of research highlights the potentially destabilizing role of autonomy and reaches pessimistic conclusions. However, it focuses on a small set of regime transitions during which territorial autonomy did coincide with ethnic mobilization and conflict (Bunce, 1999; Hechter, 2000, p. 149–152; Sambanis & Shayo, 2013, p. 316). In contrast, a second strand attains more optimistic conclusions. Using evidence from large global samples, these studies indicate that autonomy reduces ethnic mobilization and conflict in most cases, by alleviating the grievances of diverse groups (Anderson, 2014; Cederman et al., 2015; Saideman et al., 2002). This strand provides compelling evidence for the “average” case. Moreover, it pays increasing attention to the conditions under which autonomy operates. However, it has so far not distinguished stable contexts from periods of regime transitions, which constitute far more difficult environments for territorial autonomy to “work,” similar to post-conflict contexts (Cederman et al., 2015).

In this article, we address this gap. We argue that the uncertainty associated with regime transitions moderates how territorial autonomy affects ethnic mobilization and conflict. First, the lack of reliable information on the new regime’s capabilities and intentions increases the risk of strategic miscalculations between autonomous groups and the government. Second, the unpredictable consequences of transitions for future ethnic relations reduce the grievance-alleviating effect of territorial autonomy. And, third, the prospect that territorial autonomy might *itself* be withdrawn during transitions incentivizes group leaders to mobilize while the new regime is still unconsolidated. In consequence, during regime transitions, we expect autonomy to be more likely to fuel destabilizing bargaining and thereby raise the risks of ethnic mobilization and conflict. In contrast, during periods of regime stability, these escalatory dynamics are “muted” by the grievance-alleviating consequences of territorial autonomy.

We test our expectations in two quantitative, cross-national analyses. First, we investigate how autonomy affects ethnic mobilization and conflict in a comprehensive, global sample of regime transitions since the Second World War. Thereby, we avoid an exclusive focus on prominent transitions associated with conflict. Second, we revisit the already prominently discussed case of ethnic mobilization during the transition from Communism. In contrast to previous work, we shift focus from the socialist federations’ initial breakup to the consequences of “transmitted” autonomy in their successor states. Complementing our global analysis, this enables us to alleviate concerns whereby autonomy might be offered to ward off regime transitions or whereby transitions might *themselves* be induced by ethnic mobilization in the first place. In this second set of analyses, we use new fine-grained data on territorial autonomy. This enables us to study its impact in a stepwise manner.

Together, our analyses indicate that the relationship between territorial autonomy and ethnic mobilization and conflict differs between periods of regime transition and periods of regime stability. During transitions, ethnic groups with substantial territorial autonomy are not only more likely to mobilize for self-determination, but also more likely to obtain concessions, and to violently escalate their contestations. We find that this relationship is mitigated where autonomous groups are included in the central government. Thereby, they underline advice that autonomy should be combined with central government inclusion (Bakke, 2015; Cederman et al., 2015) to generate trust and enable institutionalized bargaining during regime transitions.

## Literature Review

We start by defining two key terms used in this article. First, we employ the term (territorial) “autonomy” to refer to the degree of territorial self-government available to an ethnic group. While some scholars conceive of autonomy as a dichotomous concept (Vogt et al., 2015), others conceptualize it as a matter of degree, whereby autonomy arrangements are composed of political autonomy (ethnic groups having their own executive and legislative institutions), broad policy-making competences, and independent fiscal resources (Bakke, 2015; Hooghe et al., 2016). Second, under “regime transitions” we subsume periods where a previously authoritarian regime introduces competitive elections, or vice-versa where democracy is suspended, and periods where a polity recently attained statehood (Hegre et al., 2001, p. 37).

We now discuss prior evidence on how territorial autonomy affects ethnic mobilization and conflict. A first influential body of research highlights that territorial autonomy generates opportunities for ethnic mobilization. This is because territorial autonomy increases the capabilities of ethnic groups vis-à-vis the government (Roeder, 2007). In this vein, numerous studies attribute the near-simultaneous dissolution of the three socialist federations—the Soviet Union (USSR), Yugoslavia, and Czechoslovakia—to the combination of federal structures with the sudden delegitimization of Communist rule (e.g., Bunce, 1999; Cornell, 2002; Filippov et al., 2004; Giuliano, 2006; Hale, 2004; Hechter, 2000).

**Figure 1.**
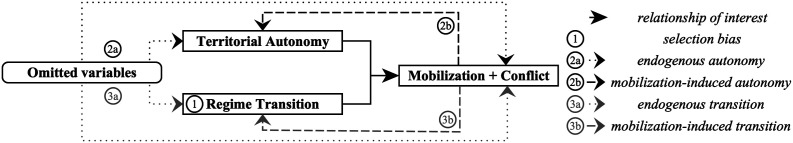
Relationship of interest and empirical challenges.

However, the evidence provided in this literature for the mobilization- and conflict-inducing consequences of territorial autonomy during transitions is faced by three empirical challenges (see Figure 1). A first challenge is *selection bias*. Most studies in this literature focus on regime transitions, during which governments’ capabilities to address challenges are particularly weak. Moreover, they focus on a prominent *subset* of such transitions in which autonomy was associated with conflict or state collapse (Anderson, 2014; Grigoryan, 2012). Hence, it remains unclear whether these findings extrapolate to other contexts. This includes other cases of regime transition, some of which may have “held together” *because* of territorial autonomy, for instance India during the post-independence period (Ahuja & Varshney, 2005).

A second challenge is posed by *mobilization-induced autonomy*. According to Anderson’s (2014, pp. 178–181) count, many ethnofederal states are “coming together” federations, with little prospect of surviving as a common state. Generalizing this argument, territorial autonomy is often provided to the most “threatening” groups in the first place (Cederman et al., 2015), especially those with substantial economic resources, large size, regional concentration (Cetinyan, 2002; Jenne et al., 2007; De la Calle, 2015, pp. 187–194), and prior mobilization (Grigoryan, 2015).

Finally, a third challenge is posed by *mobilization-induced regime transitions*. Regime transitions and ethnic mobilization may simultaneously be made more likely by the same risk factors, such as economic crises. Alternatively, regime transitions may *themselves* be initiated by subnational actors (Samuels & Abrucio, 2000) or brought about by the mobilization of already previously autonomous groups (Leff, 1999). This complicates assessments of how territorial autonomy affects mobilization and conflict during transitions. For instance, ongoing mobilization of autonomous groups may simply continue in the post-transition period, rather than be induced by it.

A second set of studies addresses the first of these challenges by investigating how territorial autonomy affects conflict in a global sample. These highlight that territorial autonomy alleviates diverse ethnic groups' grievances. Thereby, it attenuates ethnic mobilization and conflict (Bunce, 2007; Cederman et al., 2015; Cetinyan, 2002; Grigoryan, 2015; Jenne et al., 2007). Many scholars additionally argue that territorial autonomy reassures ethnic groups of their physical or cultural survival. Especially in post-conflict contexts, mobilized groups face security dilemmas, whereby they fear future repression if they lay down arms (Fearon, 1998; Lake & Rothchild, 2005). Territorial autonomy mitigates this dilemma by raising the costs for governments to renege on their promises (Hartzell & Hoddie, 2008; Mattes & Savun, 2009; Saideman et al., 2002; Walter, 2006).

Autonomy is thus strategically offered by governments to reduce grievances (Staniland, 2021, p. 135). Comparative studies indicate that it indeed decreases ethnic mobilization and conflict in most contexts (e.g., Brancati, 2006; Cederman et al., 2015; Hartzell & Hoddie, 2008). Clearly, these findings are immensely important. Yet, they do not rule out countervailing effects of territorial autonomy during regime transitions, which constitute particularly difficult environments. Following similar considerations, several studies investigate the context-specific effects of territorial autonomy in periods of transition (but without comparison to stable periods, cf. Samuels & Abrucio, 2000; Hale, 2004), or during post-conflict transitions to peace. These show that autonomy may be “too little” on its own once conflict has already escalated (Cederman et al., 2015). While these findings are instructive, the overlap between post-conflict environments and regime transitions is partial at best. In peace negotiations, autonomy is tailored to address the concerns of diverse ethnic groups. Vice-versa, in regime transitions, autonomy is frequently “transmitted” from the previous regime, and it is not evident whether autonomy offers the same kind of reassurance under the new regime.

Building on these conditional arguments, recent studies investigate the *conditions* under which autonomy exerts stabilizing effects (cf. Juon & Bakke, 2013). These highlight that autonomy is aimed at incorporating regional elites into political power. However, only their inclusion into statewide parties (De la Calle, 2015; Bakke, 2015) or statewide political networks (Roessler, 2016, pp. 142–145) improves information flows between the center and peripheral groups. Regional elites that are embedded in statewide parties can ward off challengers and avoid destabilizing outbidding; moreover, their inclusion avoids political segregation and forestalls the emergence of strong independence movements (De la Calle, 2015, pp. 17–28). Supporting this reasoning, there is evidence that autonomy is stabilizing, if it is embedded in a wider mix of power-sharing institutions (Hartzell & Hoddie, 2008), or complemented by central government inclusion (Cederman et al., 2015; Filippov et al., 2004; Mattes & Savun, 2009). These studies powerfully highlight the importance of contextual factors (cf. Hale, 2008, pp. 80–87), such as whether autonomous groups are included in the center. However, they have so far abstained from discussing contexts of regime transition, in which central government institutions are *themselves* in flux (Staniland, 2021).

In sum, previous scholarship provides important insights. However, its predominant focus on prominent and difficult cases, or the lack of distinction between transitional and stable contexts, makes is hard to assess how territorial autonomy affects ethnic mobilization and conflict during regime transitions. Several cross-national investigations illuminate how regime transitions affect conflict risks more broadly (Gleditsch, 2002a; Hegre et al., 2001; Snyder, 2000). However, these do not consider how territorial autonomy may moderate conflict risks during such transitions.

## Theory

To address these gaps, we formulate and test a theoretical argument of how territorial autonomy affects ethnic mobilization and conflict, differentiating between periods of regime transitions and regime stability. Our starting point is the conception of a group’s degree of autonomy as influenced by a three-stage bargaining process between its leadership and the government (cf. Cetinyan, 2002; Jenne et al., 2007).^1^ In this model, the ethnic group first decides whether to demand higher degrees of autonomy by mobilizing in a self-determination movement (SDM). Second, the government responds, either by offering concessions that increase the group’s degree of autonomy or by refusing to do so. Third, in case the government does not offer concessions or group leaders consider its concessions as insufficient, group leaders decide whether or not to violently escalate their demands. Each of these stages—initial mobilization, government concessions, and violent escalation—is critically affected by an ethnic group’s relative capabilities. These shape its ability to threaten the government with the costs of violent conflict in case its demands are not met and hence affect its bargaining power vis-à-vis its government.

We expand this framework by integrating arguments from previous research on how territorial autonomy increases the capabilities of ethnic groups. We distill five sets of reasons for this expectation from this literature: First, higher degrees of autonomy enable group leaders to overcome coordination problems and mobilize their members both for peaceful and violent SDMs (McGarry & O’Leary, 2009; Snyder, 2000; Bunce, 1999; Roeder, 1991). Second, where autonomy comes with regional media platforms and security forces, it increases group elites’ ability to inculcate and arm followers (Brancati, 2006; Cornell, 2002). Third, by demarcating specific territories, autonomy provides group leaders with ready-made “proto-states,” thus increasing the credibility of secessionist threats (Cornell, 2002). Fourth, the higher a group’s autonomous policy scope, the more its elites can build experience in governing, thereby making independence more feasible (Brancati, 2006; Roeder, 1991). Finally, autonomy provides transnational kin states with a local collaborator on the ground (Bunce, 2007; Cetinyan, 2002; Cornell, 2002). These boost the chances of successful secession (Jenne et al., 2007).

By increasing a group’s relative capabilities vis-à-vis its government, higher degrees of autonomy should have similar implications for their bargaining behavior as other aspects influencing the distribution of relative capabilities between these two actors. Most importantly, the higher the degree of territorial autonomy, the higher an ethnic group’s ability to threaten its government with the cost of protracted conflict in case its leadership’s demands are not met. In turn, this means that the government faces pressure to respond to the mobilization of autonomous groups with concessions that substantially increase their degree of autonomy. Finally, by raising the chance of concessions, leaders of groups with high degrees of territorial autonomy should also have higher incentives to initiate SDMs in the first place.

However, due to two de-escalating mechanisms, we are unlikely to witness the empirical implications of this mechanism for the “average” case. First, governments are less likely to grant autonomy concessions to groups that might opportunistically exploit them and mobilize for increased self-determination (Cetinyan, 2002; Grigoryan, 2015; Jenne et al., 2007). Second, even where territorial autonomy does result in an unanticipated increase in group-wise capabilities, it may “mute” elites’ incentives to mobilize, by alleviating grievances (McGarry & O’Leary, 2009, p. 6) and increasing group members’ assessments that the central government protects their life chances (Hale, 2008, p. 72). Hence, even if tempted by their increased capabilities, group elites may struggle to attain the support required to organize SDMs.

We argue that these de-escalatory mechanisms are closely linked to periods of regime stability. They depend on institutions that enable ethnic groups and their governments to anticipate and coordinate each other’s actions, such as joint statewide parties or inclusive governments (De la Calle, 2015, pp. 17–28; Bakke, 2015; Roessler, 2016, p. 143). Statewide parties facilitate the establishment of patronage networks in autonomous regions (De la Calle, 2015). Constitutional guarantees of inclusion render ethnic group members confident in the longevity of their attained territorial autonomy and allow for settling conflict through institutionalized bargaining (Filippov et al., 2004; Hartzell & Hoddie, 2008; Mattes & Savun, 2009; McGarry & O’Leary, 2009; Walter, 1997). In contrast, during regime transitions, which are almost by definition characterized by intense uncertainty, these stabilizing factors *themselves* either dissolve or become subject to change (Hale, 2008, pp. 65–89). Moreover, third parties that provide security guarantees, and thereby stabilize territorial autonomy, might turn from allies into adversaries in light of recent political changes (Gleditsch, 2002a).

We argue that the institutional instability associated with regime transitions affects the relationship between territorial autonomy and ethnic mobilization and conflict in three detrimental ways. First, during regime transitions, not only the composition of the government, but its underlying institutional regime is subject to change. For example, transitions often entail far-ranging shifts in the political paradigms governing inter-group relations (cf. Walter, 2006). This means that trust and channels facilitating bargaining between the government and group leaders need to be reestablished (Roessler, 2016, p. 143). Lacking institutionalized channels of communication, group leaders may find it difficult to anticipate how the new regime will react to SDMs (see also Bas & Orsun, 2021). Vice versa, the new, unconsolidated regime may initially lack reliable information on the capabilities and intentions of ethnic groups. This mutual information deficit critically raises the probability of strategic miscalculations, and conflict escalation, between ethnic groups and their government.

Second and relatedly, ethnic groups might not take the longevity of their attained autonomy for granted during regime transitions. Owing to changes in fundamental constitutional rules, they fear that the new regime might hollow out preexisting autonomy arrangements. For instance, it might do so by altering the mode of federal appointments or by abolishing autonomy altogether (Hale, 2008, pp. 75–76; Siroky & Cuffe, 2015). Regime transitions might also engender more exclusionary definitions of the nation more broadly. This creates fears among ethnic group members that they might become the target of exclusionary nation-building policies (Brubaker, 1996; Leff, 1999; Snyder, 2000). In the course of transitions, countries might switch international alliances, making external security guarantees void (Staniland, 2021, p. 12). Thereby, this situation resembles other environments with low trust, such as post-conflict or post-coup periods (Cederman et al., 2015; Roessler, 2016). Among group members, these anticipated threats foster the perception that they are bound together in an ethnic “community of fate” and increase ethnic identification (Hale, 2008, p. 50). Moreover, they generate fears that group members’ life chances may be adversely affected by future exploitative government policies (Hale, 2008, p. 70). Hence, during regime transitions, territorial autonomy is less suited to reduce group members’ grievances and reassure them than during more stable contexts.

Finally, regime transitions not only increase the potential benefits of mobilization, but also decrease its risks.^2^ Most importantly, for group leaders, regime transitions create a suitable window of opportunity to mobilize and reap further concessions while the new regime is still unconsolidated (Brancati, 2006; Fearon, 1998; Walter, 2006) and potentially unable to use force (Hale, 2008, p. 72). This is most evident where transitions create new states, but also applies where they change the regime type. This incentivizes ethnic elites to mobilize for further concessions or even independence in the immediate wake of regime transitions.

In sum, our arguments lead us to formulate two expectations. First, higher degrees of territorial autonomy should increase ethnic mobilization and raise the risks of violent escalation during regime transitions. In such contexts, we expect autonomy to behave in similar ways as the more stable structural capabilities commonly highlighted in bargaining models of autonomy. Thereby, during regime transitions, higher degrees of autonomy should provide more capabilities to ethnic groups, increase their mobilization, make it more likely that governments respond with concessions, and, especially if sufficient concessions are not forthcoming, render violent escalation more likely. Second, we expect the effect of autonomy during regime transitions to differ from its effect during periods of regime stability. In stable contexts, territorial autonomy is better suited to alleviate ethnic group members' grievances and reassure them, and is less likely to give rise to security dilemmas. We capture these key expectations in three testable hypotheses:

**Hypothesis 1:** *During regime transitions, higher degrees of autonomy increase the propensity of ethnic groups to initiate self-determination movements*.

**Hypothesis 2:** *During regime transitions, higher degrees of autonomy increase the propensity of governments to offer concessions to ethnic groups*.

**Hypothesis 3:** *During regime transitions, higher degrees of autonomy increase the propensity of ethnic groups to violently escalate self-determination movements, especially if the government does not offer concessions*.

## Quantitative Analysis: Global Sample

### Overarching Empirical Strategy

We test our expectations in two sets of quantitative analyses that investigate the context-specific effects of autonomy during periods of regime transitions. Although neither set-up enables us to fully address the empirical challenges identified in our literature review (see Figure 1), each of them enables us to attenuate them to variable degrees and in complementary ways (see Table 1).

**Table 1.** Addressing Key Challenges in Our Two Analyses.

	Sample 1 (global)	Sample 2 (CEE-FSU)
1. Selection bias	Global sample, 1946–2017	
2a. Endogenousautonomy	Control variables; group-fixed effects (RC)	Control variables; autonomy awarded in dissimilar bargaining environment, based on strategic considerations mostly unrelated to group-wise capabilities vis-à-vis successor states; unforeseen, simultaneous transition
2b. Mobilization-induced autonomy	Excluding mobilized groups before transition (RC)	Autonomy awarded based on strategic considerations mostly unrelated to previous ethnic mobilization
3a. Endogenoustransition	Control variables; group-fixed effects (RC)	Control variables; unforeseen, simultaneous transition
3b. Mobilization-induced transition	Accounting for mobilization in predecessor state; excluding mobilized groups before transition (RC)	Accounting for mobilization in predecessor state; exclusion of Republican-level titular groups

RC = robustness checks.

In a first step, we conduct a global analysis, covering the period since the Second World War. This comprehensive sample is best suited to help us guard against selection bias. While we cannot overcome endogeneity challenges in our observational approach, we employ strategies related to the construction of our main variables, controls, and limitation to sub-samples to mitigate the challenges of mobilization-induced autonomy and transitions, as described below. Second, we revisit the case of the transition from Communism in Central and Eastern Europe (CEE) and the Former Soviet Union (CEE-FSU). Here, we focus on autonomy that was transmitted from the pre-transition period, and its consequences for bargaining in the post-communist successor states. This allows us to further mitigate the dangers of mobilization-induced autonomy, as described in the next section. By analyzing countries undergoing simultaneous and largely unexpected transitions events, we can also alleviate concerns about endogenous transitions.

### Unit of Analysis and Data

We start by testing our hypotheses in a global sample. Our unit of analysis is the ethnic group year. Our sample encompasses all groups with a geographically distinct settlement pattern between 1946 and 2017, as given by the Ethnic Power Relations Dataset (EPR, cf. Vogt et al., 2015). From this sample, we exclude groups that rule the central government alone, to the exclusion of all other groups in the country. These groups, which EPR (Vogt et al., 2015) codes as having “dominant” or “monopoly” status, will almost by definition not engage in SDMs against the government which they control by themselves.^3^ By investigating the consequences of territorial autonomy during a comprehensive set of regime transitions around the world, we alleviate the challenge of selection bias (Figure 1, challenge 1).

We rely on the SDM dataset (Sambanis et al., 2018), updated with Cederman et al. (2022), to operationalize our dependent variables. This enables us to test hypothesis 1 and conduct a partial test of hypothesis 3 (in which we are, however, unable to account for recent concessions). This dataset contains a comprehensive list of both peaceful and violent SDMs. SDMs are identified by the presence of at least one political organization that demands increased self-determination, such as (higher degrees of) autonomy or secession. Most of these SDMs are associated with ethnic groups in our sample (Germann & Sambanis, 2021). We code an *SDM onset* for each year in which a group is involved in at least one SDM, while not being involved in one in the previous 2 years. We code a *violent SDM onset* in years where an SDM onset is accompanied by violence or where an ongoing SDM turns violent. In the SDM dataset, SDMs are coded as violent as long as they meet a comparably low threshold, given by at least 25 deaths in a given year.

Of particular concern for our study are cases where regime transitions resulted in the creation of new states. In these cases, mobilization-induced transitions are a particular concern (Figure 1, challenge 3b). Thereby, ongoing SDMs of a group may have precipitated the observed regime transition in the first place and simply continued in the respective successor state. For instance, according to our data, the SDM of Bosnian Croats first started under Yugoslav rule in 1989 and continued after Bosnia attained independence in 1992. To avoid coding a new SDM onset or escalation in such cases, we compiled a list of *predecessor groups* for all cases where a group newly enters our sample. In each case, we only code a (violent) SDM onset where a group’s predecessor was not active in a (violent) SDM in the respective predecessor state, thus mitigating this inferential danger.

Following our broad conception of transitions, we combine information on democratization and autocratization episodes, relying on the V-Dem Episodes of Regime Transition Dataset (Maerz et al., 2021), with information on the creation of new states. In our main models, we construct variables that subsume all transitions, regardless of their type. In our robustness checks (Appendix C.1), we distinguish between democratizing and autocratizing episodes and periods following the creation of new states to trace our findings further. We variably capture the conditioning role of transitions with two variables. First, we code a dichotomous variable to denote *transition periods*. This takes the value 1 for country years that are within 5 years of either democratization or autocratization processes or of newly attained statehood. Second, we capture regime transitions as a “shock,” whose magnitude decays in years after a transition event. We implement this by coding a variable regime *transition proximity* with 1 during transition years, with this value decaying in subsequent years with a 3-year half-life.^4^

Finally, to obtain information on group-wise autonomy, we rely on the dichotomous *autonomy* measure provided by the Ethnic Power Relations Dataset (Vogt et al., 2015). This has the advantage of providing extensive expert-coded and standardized information on the de-facto autonomy of each group in this global sample. However, the dichotomous coding of this variable restricts our ability to fully test our expectations, as it does not enable us to capture the hypothesized impact of the *degree* of autonomy, nor to consider concessions thereof. We address these limitations with a more fine-grained measure in our second sample.

Across our models, we control for factors that might influence both a group’s degree of autonomy and its involvement in SDMs or that might simultaneously increase the probability of regime transitions and ethnic mobilization (Figure 1, challenges 2a/3a). At the group level, we control for politically *most powerful* status,^5^ central government inclusion, relative size (all three based on Vogt et al., 2015), distance from the state border (logarithmized), whether a group is subject to irredentist claims from a kin state government (Cederman et al., 2022), whether there are simultaneous SDMs involving a kin group (based on Germann & Sambanis, 2021; Vogt et al., 2015), and the percentage of its settlement area covered by discovered petroleum reserves (based on Lujala et al., 2007). At the country-level, we control for the level of democracy, given by a corrected version of the Polity index (normalized to a range of 0–1, Marshall et al., 2019), to exclude the problematic PARREG component (Vreeland, 2008), GDP per capita, and population size (both logarithmized).^6^ We also include a term for the total number of SDMs in the previous year. Finally, to account for time dependence, we include a cubic term measuring the years since a group last experienced the respective event we are modeling. In appendix A, we provide descriptive statistics for these variables.^7^

### Specification and Results

We are now in a position to quantitatively investigate how territorial autonomy affects (violent) SDM onsets in the comprehensive set of regime transitions offered by our global sample. Our unit of analysis is the ethnic group *i* in country *c* in year *t*. We focus on our two dependent variables: *SDM onset* and *violent SDM onset*. As groups with an ongoing (violent) SDM in the previous year can by definition not see a (violent) SDM onset, we drop these from our sample, respectively.^8^ As all our dependent variables are dichotomous (y_*i,c,t*_ ∼ binomial(π_*i,t*_,1) see above), we rely on a series of logistic regressions of the following basic form

(1) $$logit\left(\pi_{i,c,t}\right)=\beta_{0}+\beta_{1}\;{Autonomy}_{i,c,t}\times {Regime\;transition}_{c,t}+\beta_{2}X_{1i,c,t}+\beta_{3}X_{2,c,t}+{ɛ}_{i,c,t}$$

We estimate four models (see Table 2), reporting country-clustered standard errors. In our first two models, we interact autonomy with our binary variable *transition period*, as constructed above. In our second two models, we interact it with our continuous variable for regime *transition proximity*. X_1*i,c,t*_ and X_2*c,t*_ are our group- and country controls, respectively, as operationalized in the last section. In all models, we include region- and year-fixed effects to account for differing baseline risks in different world regions and global shocks.^9^

**Table 2.** The Effects of Autonomy on Ethnic Self-Determination Movement (SDM) Onsets and Violent SDM Onsets, During Regime Transitions and Other Time Periods.

	SDM onset	Violent SDM onset	SDM onset	Violent SDM onset
	Model 1	Model 2	Model 3	Model 4
Autonomy	−.096	−.335	−.097	−.405
	(.433)	(.366)	(.448)	(.398)
Transition (0–5)	.675^***^	−.076		
	(.225)	(.218)		
Autonomy x transition (0–5)	1.111^**^	1.125^***^		
	(.512)	(.420)		
Transition proximity			.909^***^	.008
			(.256)	(.262)
Autonomy x transition proximity			1.119^**^	1.307^***^
			(.552)	(.497)
Most powerful	−1.042^**^	−.736	−1.026^**^	−.745
	(.441)	(.572)	(.439)	(.568)
Included	−.292	−.593^**^	−.255	−.586^**^
	(.318)	(.269)	(.319)	(.268)
Group size	−.424	.066	−.448	.059
	(.642)	(.628)	(.637)	(.629)
TEK irredentism	.048	.885	.025	.851
	(.715)	(.769)	(.715)	(.773)
TEK SDMs	−.008	.290	−.0002	.297
	(.238)	(.205)	(.235)	(.203)
Petroleum % in settlement area	.502	.430	.493	.398
	(.339)	(.319)	(.336)	(.321)
Previous SDMs in country	.025^*^	−.016	.020	−.021
	(.013)	(.016)	(.013)	(.016)
Normalized polity score	.591^*^	−.018	.531^*^	−.072
	(.311)	(.517)	(.314)	(.519)
Distance border (log)	−.053^***^	−.033	−.053^***^	−.033
	(.013)	(.023)	(.013)	(.022)
GDP p.c. (log)	−.081	−.459^***^	−.087	−.464^***^
	(.140)	(.173)	(.141)	(.177)
Population (log)	.112	.058	.121	.062
	(.079)	(.087)	(.079)	(.089)
Ongoing SD		2.270^***^		2.281^***^
		(.270)		(.270)
Constant	−3.082^***^	−1.134	−3.181^***^	−1.134
	(1.100)	(1.130)	(1.113)	(1.170)
Wald *χ2*				
*β*_Autonomy x transition (0-5)_ *= 0*	4.7086^*^	7.1894^**^		
*β*_Autonomy_ *+ β*_Autonomy x transition (0-5)_ *= 0*	7.881^**^	9.7684^**^		
*β*_Autonomy x transition proximity_ *= 0*			4.1186^*^	6.9286^**^
*β*_Autonomy_ *+ β*_Autonomy x transition proximity_ *= 0*			7.3645^**^	10.789^**^
N	24,466	31,044	24,466	31,044
Log likelihood	−1172.670	−866.713	−1166.895	−865.265
AIC	2533.341	1923.425	2521.790	1920.530

Country-clustered errors in parentheses. Cubic term for time dependence of dependent variable and year- and region-fixed effects included but not reported.

***p < .01, **p < .05, *p < .1.

**Figure 2.**
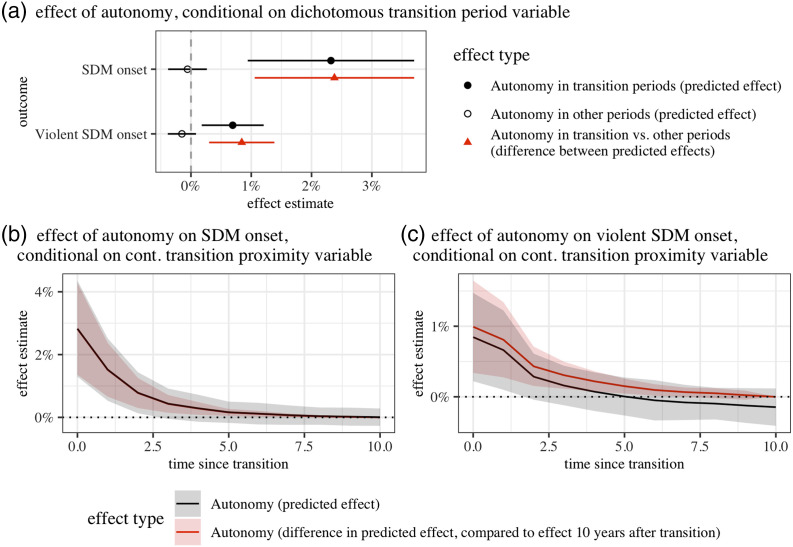
First and second-differences in the predicted probability of (violent) SDM onset, depending on autonomy and transition periods. Based on models 1–4 in Table 2.

Because of the conditional nature of our hypotheses, we provide Wald tests at the bottom of our results tables. These test for equality between the coefficients of *autonomy* in transition and other contexts and for joint significance of *autonomy* and its interacted terms. This allows us to avoid relying on the statistical significance of interacted coefficients alone (cf. Berry et al., 2010). Additionally, in Figure 2, we plot two types of effects, based on the observed values in the sample (Hanmer & Ozan Kalkan, 2013): First, the (first) difference in predicted probabilities of (violent) SDM onset between autonomous and non-autonomous groups, depending on regime transitions. And second, the (second) difference between these effects. This corresponds to the difference in how autonomy affects these outcomes during transitions, as compared to stable contexts (cf. Berry et al., 2010).

Our results offer tentative support for hypotheses 1 and 3. Autonomy is strongly associated with the onset of (violent) SDMs. However, as we have argued, this association only holds during periods of regime transition (models 1–2) or in the initial years following a transition (models 3–4), respectively. In contrast, we find no statistically significant association of autonomy with both outcomes during other time periods (models 1–2) or as the last transition becomes less proximate (models 3–4).

In line with previous research on regime transitions and conflict (Cederman et al., 2010; Regan & Bell, 2010), we also find that SDM onsets become more likely during transition periods (model 1) or closer to the last transition more generally (model 3), irrespective of territorial autonomy.

### Additional Analyses

In additional analyses, we probe whether contextual factors highlighted by previous research might account for the conditional relationship between autonomy and (violent) SDM onsets (Tables A2–A8 in appendix B.1). For this purpose, we distinguish between autonomy with/without simultaneous government inclusion and autonomy before/after initial violence has broken out (Bakke, 2015; Cederman et al., 2015; De la Calle, 2015). Moreover, we account for how autonomy and transitions interact with ethno-regional organizations (Brancati, 2006; Vogt et al., 2015), economic inequality (Bakke, 2015; Deiwiks et al., 2012), ethnic cleavages (Bakke, 2015), and changes in the government’s identity more broadly.

Our findings remain similar in all of these procedures, with one important caveat: they indicate that the positive association of autonomy with (violent) SDM onset during regime transitions is limited to constellations where an autonomous group is excluded from government. Conversely, we do not attain a similar relationship in rarer cases where a group is, in addition to possessing autonomy, *itself* included in the new regime. This highlights an important scope condition of our findings: As we have argued, transitions generate uncertainty and distrust among autonomous groups toward the government’s future behavior, and thereby incentivize them to initiate and escalate SDMs. However, similar to post-conflict contexts (Cederman et al., 2015), this danger is mitigated where autonomous groups are *themselves* part of the new regime.

We also distinguish between different *types* of transitions (Table A11 in Appendix B.3). We find that the two investigated stages (mobilization and escalation) are driven to different degrees by periods of democratization, autocratization, and state creation. We find that, during democratizing periods, autonomy has a strong effect on mobilization, but not on violent escalation. This echoes findings that autonomy may foster peaceful ethnic mobilization (e.g., Hechter, 2000). New democracies are likely more transparent in building new institutions and thereby in a better position to settle self-determination disputes peacefully (cf. Hale, 2008, p. 81). Conversely, we find that, during autocratizing periods and especially following the creation of new states, autonomy entails a higher risk for a violent escalation. This may reflect the fact that these new regimes may be unable to accommodate self-determination demands. Autocratizing regimes and new states may want to signal regime strength and forcibly underline the inviolability of new international borders to deter other potential challengers (Walter, 2006), thereby making SDMs more prone to escalation.

Finally, we conduct numerous general robustness checks (Appendices B.2–B.4). We probe reverse causation and endogeneity concerns further by excluding groups with SDM involvement before the last regime transition, incorporating group-fixed effects, and conducting causal sensitivity analyses. Moreover, we employ different operationalizations of key variables, sample alterations, and alternative specifications. Our findings are remarkably robust to each of these alterations and appear unsensitive to the presence of omitted confounders.

## Revisiting Autonomy and Ethnic Mobilization in the CEE-FSU Region

Our findings based on this global sample provide robust evidence in line with our hypotheses 1 and 3. However, two critical caveats remain. First, as discussed in our literature review (see again Figure 1), we are faced by difficult empirical challenges. Multiple states in our sample are “coming-together” federations of territories with a history of prior mobilization (Anderson, 2014). Moreover, governments may anticipate and strategically counter SDMs with autonomy before they become observable in our data. Hence, in our observational analyses, we still risk mistakenly attributing an explanatory role to autonomy where states unsuccessfully granted autonomy to counter the looming risks of transition or where the transition was induced by mobilized groups in the first place.

Second, to obtain information on group-wise autonomy in our extensive, global sample, we had to rely on a binary measure for autonomy. This does not allow us to study how its *degree* affects ethnic mobilization. It also bars us from investigating autonomy concessions which are crucial for testing our stepwise hypotheses 2 and 3. Concessions in the degree of autonomy are frequent and consequential, as the case of the Russian Federation demonstrates, which allowed expansions in the degree of autonomy for *all* its ethno-regional territorial units in the early 1990s (Roeder, 2007; Zuber, 2011).

We address these caveats with a second set of analyses in which we focus on ethnic groups settling in areas of the Former Soviet Union (FSU) and CEE after the 1989 transition from Communism, covering the period 1990–2017. In the next sub-sections, we explain the rationale for this case selection, describe the evolution of autonomy in these cases, introduce our new territorial autonomy measure, present a second set of quantitative models, and discuss our results.

### Rationale for Case Selection

A focus on ethnic groups in the former socialist states in the CEE-FSU region is well-suited to complement our global analysis for three reasons. First, by focusing on the post-1989 period, our sample excludes secessionist movements at the level of the Soviet and Yugoslav member Republics, which precipitated the transition from Communism in the first place. In contrast to earlier research on this region, we thereby shift focus away from how territorial autonomy affected the initial disintegration of the socialist federations (Brubaker, 1996; Bunce, 1999; Leff, 1999; Roeder, 2007; Slezkine, 1994; Suny, 1993).^10^

Instead, we study the consequences of “transmitted” autonomy for ethnic groups settling in the successor states of the FSU and Central and Eastern Europe (CEE-FSU), *after* the initial transition (1990–2017). Thereby, we focus on autonomy arrangements that the Soviet and Yugoslav successor states inherited from the *second*-level federal institutions in the Soviet Union and in Yugoslavia. For example, we study the effect of transmitted autonomy on the mobilization of Hungarians in Serbia or of Bashkirs in the newly independent Russian Federation, or the lack thereof for the (non-)mobilization of Serbs in newly independent Slovenia. As our analyses remain observational, these strategies cannot overcome the challenge of mobilization-induced transitions. However, they help us further mitigate the risk of attaining biased results (Figure 1, challenge 3b).

Second, while some first-order Soviet and Yugoslav republics had a history of independent statehood before the formation of these federations, this is not the case for second-order autonomous territories within the Soviet or Yugoslav Republics, and neither for other states in the CEE region that transitioned from communism after 1989. The literature is concerned that in ethno-federal states whose regions have a history of independent statehood, instability can be traced back to the founding period (Anderson, 2014). While our global sample includes such cases “coming-together” federalism, our analysis of autonomous territories and SDMs in the post-communist successor states is based on a sample of cases that excludes those types.

Instead of arising through “coming-together” processes, the autonomous regions in our sample were all created under communist rule, and amid actor constellations that were largely dissimilar from the post-transition conflicts. Importantly, these actors’ strategic calculations were often unrelated to the later interactions between ethnic groups and their governments in the successor states. When federal or autonomous institutions were inaugurated, the communist party controlled the governments at all levels; therefore, the threat of potential future SDMs was temporally removed. While our observational analyses cannot exclude such concerns altogether (see next paragraph), this temporal and actor-related disjuncture allows us to further mitigate the challenges of endogenous and mobilization-induced autonomy (Figure 1, challenges 2a/2b).

Third, the cases included in this second set of analyses do not vary with regards to regime transition, our conditional variable. However, the 1989 transition was simultaneous, and demonstrably unexpected by actors across the region (Beissinger, 2002). Focusing on this narrower set of cases thereby further decreases the role of autonomy strategically granted by governments trying to ward off regime transitions. Thereby, we are able to mitigate the challenge of endogenous transitions for one specific sub-sample (Figure 1, challenge 3b).

### The Evolution of Autonomy Arrangements in the CEE-FSU Region

We now expand these arguments by discussing the historical evolution of autonomy arrangements in the CEE-FSU region in more detail. The historical and political context when territorial autonomy was first provided to ethnic groups in our sample, going back in the USSR to the 1920s and 1930s (Cornell, 2002; Slezkine, 1994; Suny, 1993), was highly dissimilar from the post-transition context after 1989. In this formative period of territorial autonomy in the CEE-FSU region, early Soviet nationalities ideology, called “korenizatsiya,” was adopted to provide socialism with ethnic roots. Forming but one part of this wider policy, federal Republics and autonomous regions were created, with distinct strategic goals in mind. These goals were largely unrelated to the capabilities of the successor states’ ethnic groups, which enter our sample.

In some cases, external strategic considerations dominated that were insulated entirely from internal bargaining dynamics. For instance, at the USSR’s Western frontier, the Belarusian and Ukrainian Republics and the Moldovan Autonomous Republic appealed to what the Communist Party sought to portray as the respective titular group’s suppressed co-ethnics living under Polish and Romanian rule. Outside the USSR, similar arrangements were imposed on other states, culminating in the establishment of a (short-lived) Hungarian Autonomous Region in Romania in 1952 (Bottoni, 2018).

In other cases, internal considerations related to domestic ethnic power relations played a larger role. However, again they were not directly affected by characteristics influencing the later relative capabilities between the successor states' ethnic groups and their governments in our sample. Instead, they followed the aims of making the USSR’s “hardline” economic reforms more “palatable to the larger population” (Martin, 2001, p. 21), and to counterbalance the power of the titular nations within the respective Republics (Martin, 2001, p. 33). For instance, some groups (for example, the Jews and Kalmyks) were moved to new “autonomous homelands” through resettlement programs, both for economic reasons and to create artificial minority-majority enclaves with self-government (Martin, 2001, p. 43).

In several cases, including some where conflict erupted after the breakdown of the USSR, autonomous regions were created on political-strategic grounds, and not along ethnic boundaries or previous lines of conflict. For instance, in the Caucasus, the dividing lines between culturally close groups were reinforced to mitigate the risk of joint action against the center (Cornell, 1999, pp. 187–188). Conversely, in Central Asia, “korenizatsiya” built on weak, embryonic identities, and engineered new nations in a top-down fashion (Martin, 2001; Cornell, 1999). In sum, where the USSR’s decisions to create autonomous territories were affected by considerations related to ethnic groups' capabilities, these centered mostly on the purpose of weakening Republican-level titular groups. These groups (such as Azerbaijan’s Azeri) generally do not enter our sample, as they monopolize government control in the respective successor states and do not engage in SDMs within them.

These autonomy provisions mostly remained in place until 1989 and were not substantially affected by attempts to ward off a looming regime transition. While the USSR’s multi-tiered system of ethnic autonomy was occasionally adjusted, this process was not generally driven by strategic bargaining. Illustrating their weak bargaining power, ethnic minorities were disproportionally targeted by Stalinist terror, aimed at eradicating potential rivals, intelligentsia, and remnants of civil society (Alexopoulos, 2003, p. 57).^11^ Additionally, the bargaining space of autonomous groups was clearly limited by the political agenda of the Communist party (Anderson, 2014; Grigoryan, 2012; McGarry & O’Leary, 2009; Roeder, 2007, pp. 145–6). This is less the case for Yugoslavia and Czechoslovakia, where domestic considerations and bargaining were more consequential for changes in the federal order of the states, resulting in the autonomy of Kosovo and the creation of separate Czech and Slovak member Republics in the 1960s–1970s. We consider this important distinction in our analyses.

**Figure 3.**
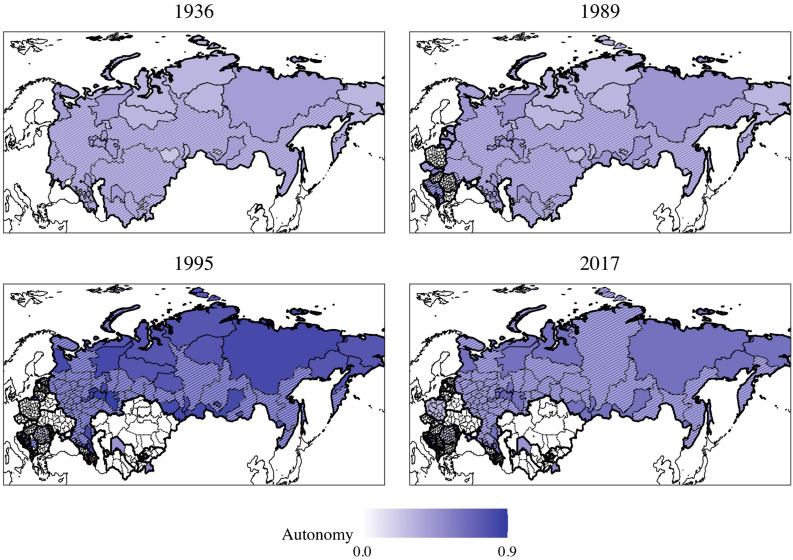
Evolution of territorial autonomy in former socialist states in the CEE-FSU region, 1936–2017. Note: Territorial units that are designated to post-transition majority groups or where these form the demographic majority are hatched.

After 1989, this situation changed suddenly and simultaneously across the region, as previously inconsequential autonomy arrangements became politically relevant almost overnight (Beissinger, 2002; Filippov et al., 2004). Across the former socialist states, ethnic groups obtained substantively meaningful autonomy. This unexpectedly increased their bargaining power vis-à-vis the successor state governments, who were predominantly constituted by the former Republican-level titular groups. Figure 3 illustrates this continuity of territorial autonomy arrangements and confirms the continued importance of autonomy transmitted from the pre-transition period, using our new fine-grained data for territorial autonomy (see below). We see a number of important changes, yet no uniform trend: autonomy expanded in Russia in the early 1990s, but was subsequently reined in (cf. Zuber, 2011). Conversely, the South Caucasus republics and Yugoslavia show a pattern of initially downgraded autonomy, followed by restorations or even expansion thereof.

Importantly for our analysis, most conflicts in the successor states entering our sample are not associated with bargaining in the pre-transition period. Hence, it is unlikely that these affected the transition itself or influenced governmental behavior toward the involved groups in the pre-transition period. There are important exceptions to this rule, especially in Yugoslavia. However, the actor constellations and driving factors for the introduction of these groups’ territorial autonomy in the 1920s–1940s were largely unrelated to the bargaining situation characterizing the post-1989 conflicts that we study.

This can be further illustrated with three examples. First, the USSR’s Bashkirs mobilized both before and after the 1989 transition. However, their “homeland,” the Republic of Bashkortostan, was created for reasons unrelated to both their prior mobilization and to potential concerns of a looming transition. Instead, its creation allowed the USSR to undermine the political links between Bashkirs and Tatars (Schafer, 2001). Second, the Nagorno-Karabakh conflict erupted in both the pre- and post-transition periods. However, Nagorno-Karabakh’s initial autonomy was not negotiated between the Azerbaijani Republican government and its Armenian minority (whose interactions enter our sample). Rather, it was imposed by the Soviet Union to instrumentally alter the balance of strength between the Armenian and Azerbaijani Soviet Republics (Broers, 2019, pp. 24–27). Finally, the conflict between Moldova and Transnistria was active in both the pre- and post-transition periods. However, again, Transnistria was not created as a response to previous mobilization. Rather, in the inter-war period, a Moldovan autonomous republic was created, corresponding in large parts to the territory of the de-facto Transnistria. It was a foreign policy instrument, helping to raise the credentials of the Soviet Union as a national liberation project for the Romanian part of Bessarabia (which in 1940 became Soviet), whereas the Transnistria of 1991 sought independence from Moldova (King, 1998, p. 60).

### Expanded Data for Territorial Autonomy

For our CEE-FSU sample, we constructed a new, continuous measure for the degree of territorial autonomy available to each group. This addresses the second caveat highlighted above. Most importantly, this new measure enables us to fully test hypotheses 2 and 3, both of which are concerned with concessions that increase a group’s *degree* of autonomy. Our continuous variable, ranging from 0 to 1, mirrors the degree of territorial self-government available to a group. It is based on state constitutions and autonomy statutes, taking maximum values where all group members enjoy formally entrenched, substantial policy, fiscal, and political autonomy in their settlement area. Its underlying indicators capture each autonomous region’s ability to independently formulate its policies, how expansive its policy scope is, its taxing and borrowing competencies, and the existence of independently selected legislatures and executives (see Appendix C for details).

We use this new measure to operationalize two of our main variables in our second set of analyses. First, we create a measure for each group’s *autonomy in 1989*, which forms the key independent variable across our models in this second sample. We do so by calculating the degree of autonomy, ranging from 0 to 1, enjoyed by members of a group immediately before the collapse of Communist rule in 1989 in their settlement territory that overlaps with the respective predecessor state.^12^ Second, we code a variable for *concessions* in years where a group’s degree of autonomy increased by .1 or more as compared to the previous year. We use this variable in our models that focus on the second (the government’s decision to award concessions) and third (the ethnic group’s decision to escalate contestations, depending on prior concessions) stages of the bargaining process (which correspond to our hypotheses 2 and 3).

### Specification and Results

We now quantitatively examine how autonomy in 1989 affected ethnic mobilization in the post-socialist successor states in the CEE-FSU region between 1990 and 2017. Similar to our global analysis, our unit of analysis is the ethnic group *i* in country *c* in year *t*. Again, we exclude groups that rule the central government alone (Vogt et al., 2015). Thereby, we exclude the analytically more problematic titular groups that broke away immediately from the socialist federations and ruled alone in their successor states (for example, the Estonians in Estonia).

Analogously to our global analyses, we are interested in bargaining over self-determination in the temporal vicinity of transition periods. We hence limit our sample to years with an ongoing or recent transition, as identified by our dichotomous *transition (0–5)* variable, or years between 1990 and 1994 (i.e., 5 years after the initial transition shock). Thereby, we exclude SDM onsets and escalation processes during the initial transition process (1989) and in years where the successor states were more consolidated from our analysis. Again, we only code SDM onsets and escalation where a group was not involved in a (violent) SDM in the respective predecessor state immediately before the transition (see above).

Capitalizing on our new fine-grained measure for territorial autonomy, we are able to study all three steps of bargaining over autonomy for this sample: SDM onset, concessions, and violent escalation, depending on recent concessions. We rely on a series of logistic regressions of the following basic form

(2) $$logit\left(\pi_{i,c,t}\right)=\beta_{0}+\beta_{1}\;{Autonomy\;\left(1989\right)}_{i,c,}+\beta_{2}X_{1i,c,t}+\beta_{3}X_{2c,t}+{ɛ}_{i,c,t}$$

We estimate three models, whose dependent variables correspond to the three stages of bargaining between ethnic groups and their government (Table 3). Model 5 estimates the impact of a group’s autonomy in 1989 on its propensity to initiate SDMs in the post-1989 period (stage 1).^13^ The next two models turn to interactions between already mobilized groups and the government. Hence, we subset our sample to groups with an ongoing SDM in the previous year. Model 6 estimates the impact of a group’s autonomy in 1989 on its probability to attain further concessions (stage 2).^14^ Model 7 estimates the impact of autonomy in 1989 on the probability that an already ongoing SDM escalates into violence (stage 3), depending on a group’s autonomy in 1989 and recent concessions in the last 5 years.^15^ In all models, our group- (X_1*i,c,t*_) and country controls (X_2*c,t*_) are analogous to our global analyses.

**Table 3.** The Effects of Autonomy Transmitted From the Pre-Transition Period (1989) on Ethnic Self-Determination Movement (SDM) Onset, Resident State Concessions During SDMs, and Violent Escalation of Ongoing SDMs (CEE-FSU Sample).

	SDM Onset	Concession During SDM	Escalation During SDM
	Model 5	Model 6	Model 7
Autonomy (1989)	6.362^*^	2.778^***^	2.914^**^
	(3.840)	(1.054)	(1.215)
Recent concession			−.535^*^
			(.320)
Most powerful	−9.453^***^	−17.956^***^	−15.536^***^
	(2.815)	(2.155)	(2.760)
Included	−.901	.475	1.120
	(1.315)	(.939)	(1.076)
Group size	11.739^***^	4.519	−4.221
	(3.869)	(7.095)	(7.746)
TEK irredentism	1.549	−.226	−14.159^***^
	(1.562)	(.541)	(1.749)
TEK SDMs	−.248	−.526	.318
	(.966)	(.430)	(.867)
Petroleum % in settlement area	−.749	.927^*^	1.531^*^
	(1.553)	(.555)	(.807)
Previous SDMs	.030	.141^***^	.050
	(.039)	(.027)	(.038)
Normalized polity score	.946	1.856	1.204
	(1.533)	(1.167)	(1.206)
Distance border (log)	−.097^**^	.074^***^	−.303^**^
	(.040)	(.019)	(.126)
GDP p.c. (log)	1.076^*^	−1.753^**^	−.489
	(.589)	(.864)	(.708)
Population (log)	−.292	−.134	−.549
	(.398)	(.363)	(.361)
Constant	−16.469^**^	9.434	−2.021
	(7.481)	(6.941)	(6.829)
N	879	429	969
Log likelihood	−63.073	−71.948	−55.390
AIC	158.145	169.895	144.779

***p < .01, **p < .05, *p < .1. Country-clustered errors in parentheses. Cubic term for time dependence of dependent variable included but not reported.

**Figure 4.**
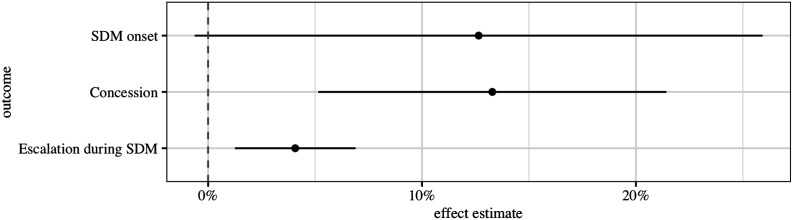
First difference in the predicted probability of SDM onset, concessions during SDM, and escalation during SDM, depending on autonomy in 1989. Based on models 5–7 in Table 3.

We again visualize the substantive meaning of our results, using the observed values approach (Hanmer & Ozan Kalkan, 2013) (Figure 4). Throughout our discussion, we illustrate our findings with several influential cases in our analysis (see Appendix D, Figure A4).

Our results offer evidence in accordance with our expectations. In line with hypothesis 1, we attain a positive, statistically significant association of autonomy transmitted from 1989 with the onset of SDMs in the transition period (model 5). Corroborating our hypothesized mechanism, the cases underlying this association indicate that previously autonomous groups were more likely to claim self-determination during years closer to the initial 1989 transition. These include the prominently discussed case of minorities during the Yugoslav disintegration: the Bosniaks (1990), Macedonians (1990), Montenegrins (1990), and Hungarians (1992) where domestic bargaining played a larger role in determining initial autonomy (Anderson, 2014; McGarry & O’Leary, 2009). Yet similar relationships also apply to a larger number of predominantly Western Russian groups that inherited high degrees of autonomy and quickly experienced SDM onsets after the 1989 transition, such as the Udmurt, Komi, Kalmyks, Karelians (all in 1990), and Kabardins (1991).

In line with hypothesis 2, we find that a group had a higher chance of obtaining further concessions if it already possessed high degrees of autonomy in 1989 (model 6). Again, this relationship is reflected in several prominently discussed cases, such as the Montenegrins in Serbia and Montenegro (2003). Yet, it also applies to the afore-mentioned Western Russian groups, which attained autonomy concessions almost immediately following their initial mobilization in an SDM, owing to their institutional resources and the concurrent weakness of the Russian state immediately after the transition (Zuber, 2011).

Finally, in line with hypothesis 3, we find that SDMs of groups with high degrees of transmitted autonomy were more likely to escalate, especially if they are not offered concessions in response to previous mobilization (model 7). Cases illustrating this relationship go beyond the inferentially problematic groups in Yugoslavia (Croats, Slovenes, both 1991; see robustness checks below). Indeed, this escalatory effect similarly applies to diverse groups such as the Abkhaz in Georgia (1992, 1997, 2001), the Pamiri Tajiks in Tajikistan (1992), and the Ingush in Russia (2007).

### Additional Analyses

Again, we conduct numerous additional analyses (Appendix E). First, we probe the robustness of our findings to different “time windows,” variably including *all* years between 1990 and 2017 or only those in the immediate aftermath of the USSR’s disintegration (Tables A22 and A23). Second, we variably limit our sample to states with significant minority population shares and to the FSU—for which we additionally replace our 1989 measure with a temporally less proximate 1936 measure for “transmitted” autonomy (Tables A24-A26). In the latter sub-sample, internal bargaining dynamics played a less substantial role than in more problematic cases, such as Yugoslavia and Czechoslovakia. This helps us mitigate concerns of endogenous and mobilization-induced autonomy (Figure 1, challenges 2a/2b).

Third, we incorporate additional control variables that capture important developments in the post-1989 period and bargaining in the pre-1989 period (Tables A27-A29). One concern is that results might be driven by the withdrawal of autonomy in the course of the transition after 1989. In one check, we control for autonomy losses since 1989 to make sure the positive association between autonomy in 1989 and destabilizing bargaining is not driven by such instances (Germann & Sambanis, 2021; Siroky & Cuffe, 2015). In another, we account for reputational dynamics whereby governments might be reluctant to offer concessions during unstable transition periods (Walter, 2006). Finally, we incorporate a variable for SDM involvement before the 1989 transition, helping us further address concerns of mobilization-induced autonomy (Figure 1, challenge 2b).

Fourth, we adopt an alternative empirical approach. Instead of estimating the consequences of autonomy in 1989 directly, we use this variable to instrument for each group’s time-variant degree of autonomy in the post-transition period. Reassuringly, our results do not appear overly sensitive to any of these alterations and alternative approaches (Table A30).

In addition, we also probe two key assumptions underlying our case selection. First, we have argued that bargaining dynamics were muted during Communist rule, helping us mitigate the dangers of mobilization-induced autonomy (Figure 1, challenge 2b). To probe this assumption, we investigate whether mobilization in SDMs predicts changes in each group’s degree of autonomy before 1989 (Table A31 in appendix E.5). We find an effect that is of minor magnitude, but statistically significant. Our analyses reveal that this is driven by Yugoslavia and Czechoslovakia, where strategic bargaining played a larger role. For the more limited sample comprising only the Soviet Union, we find no such relationship (Tables A25-A26 in appendix E.2). Reassuringly, our findings remain robust when limiting our sample to the smaller set of cases comprised by the FSU. Second, and relatedly, we have also argued that a group’s transmitted autonomy should be largely unrelated to its future bargaining power vis-à-vis the respective successor states. To test this assumption, we reverse our main analyses, probing whether we can predict a group’s autonomy in 1989 with its post-transition SDM onsets and SDM escalation events (Table A32 in appendix E.5). Reassuringly, while we obtain positive associations for both factors, these clearly fail to reach statistical significance.

## Conclusion

In this article, we have investigated how territorial autonomy affects ethnic mobilization and conflict during regime transitions. Thereby, we expand previous research on regime transitions and conflict more broadly (Cederman et al., 2010; Hegre et al., 2001). We do so by bringing in the role of territorial autonomy, which forms one of the most prominent institutional devices aimed at securing peace in multiethnic countries. By focusing on regime transitions, we also contribute to an increasing body of research that has investigated the context-specific consequences of territorial autonomy. Analogously to these studies, we move on from investigating *whether* autonomy works to probing the *circumstances* under which it does (e.g., Bakke, 2015; De la Calle, 2015; Hale, 2004; Juon & Bakke, 2023; Siroky & Cuffe, 2015).

Our results indicate that autonomous territories during regime transitions may be the “wrong place, at the wrong time” for inter-ethnic stability and peace. Whereas territorial autonomy may inhibit escalatory bargaining between ethnic groups and the government during periods of regime stability, our results highlight that higher degrees of autonomy may have destabilizing consequences during regime transitions. The uncertainty entailed by transitions makes miscalculations between governments and autonomous groups more likely, increases fears that the new regime will violate group rights, and creates incentives for groups to demand further concessions while the new regime is still unconsolidated.

Our analyses indicate that these risks are mitigated where autonomous groups are *themselves* part of the new regime. This echoes research that highlights the importance of combining autonomy with central government inclusion, particularly in difficult contexts (Bakke, 2015; Cederman et al., 2015). Together, these results suggest that, during regime transitions, autonomous groups should be incorporated into the central government, in order to avoid miscalculations, reduce uncertainty, and support institutionalized bargaining.

Our analyses inevitably only represent one puzzle piece in a larger endeavor to understand the context-dependent consequences of territorial autonomy. In our analyses, we have focused on autonomy as an overarching strategy, mostly in isolation from other factors. Yet, our arguments clearly hinge on specific institutions whose relative importance should be disentangled further (Bakke, 2015). Additionally, the merits of combining territorial autonomy with other peace-building institutions should be explored in fuller detail than has been possible in our study. Our results highlight the benefits of combining autonomy with central government inclusion. However, other domestic or international institutions might similarly safeguard against the destabilizing consequences of territorial autonomy during regime transitions.

In sum, our evidence suggests that territorial autonomy on its own may be less suited to safeguard inter-ethnic peace during regime transitions. Obviously, this does not amount to a blanket rejection of territorial autonomy, whose de-escalatory consequences are demonstrably augmented during contexts of relative regime stability. However, our findings highlight the vital importance of remaining alert to its potential risks for mobilization and conflict during regime transitions. Thereby, they underline the necessity of finding avenues to mitigate these risks, for example, but not limited to, by incorporating autonomous groups’ representatives into the central government.

## Supplemental Material

Supplemental Material - The Wrong Place at the Wrong Time? Territorial Autonomy and Conflict During Regime Transitions Click here for additional data file. Supplemental Material for The Wrong Place at the Wrong Time? Territorial Autonomy and Conflict During Regime Transitions by Andreas Juon and Daniel Bochsler in Comparative Political Studies

## Notes

1. As we highlight in our discussion of autonomy in the Central and Eastern Europe-Former Soviet Union region, autonomy may also be the result of considerations unrelated to strategic bargaining, such as regional norms, ideology, and efforts to integrate previously disparate territories into a single country (Brancati, 2008).

2. As highlighted by Hale (2008, pp. 80–87), other contextual factors influence ethnic groups’ assessments of risks and benefits of mobilizing for self-determination. These include the “thickness” of ethnic divisions, past discriminatory treatment, institutional change more broadly, elite framing, and the institutional mediation of group interests at the national level. While these factors do not form a central part of our framework, we attempt to partially account for them in our main analyses and more extensively so in our robustness checks (Appendix B).

3. See Germann and Sambanis (2021), who similarly exclude such groups from their sample.

4. This is analogous to how Bormann and Savun (2018) capture the “decaying” signal of autonomy concessions.

5. We identified the group with the highest power status (Vogt et al., 2015) in each country year. If multiple groups jointly hold the highest status (e.g., Yugoslavia) we identify the largest of these groups as the most powerful, analogously to Bormann et al. (2017) (e.g., in the case of Yugoslavia, the Serbs).

6. Data for both taken from the Penn World Tables 7.1 (Heston et al., 2012), updated with World Bank (2020) and Gleditsch (2002b).

7. Replication materials and code can be found at Juon and Bochsler (2023).

8. From a total 33′260 group years, this results in the omission of 8794 group years with ongoing SDMs and 2216 group years with ongoing violent SDMs, respectively.

9. We employ a six-fold classification of regions: Americas, Asia, Europe, MENA, Oceania, and sub-Saharan Africa.

10. Some studies have previously discussed post-transition bargaining of a limited number of second-level federal units (Bunce, 2007; Bunce & Watts, 2005; Cornell, 2002; Grigoryan, 2015). Hale (2008, pp. 216–225) compares across all three levels, and includes the period of transition. In contrast to these studies, we conduct a quantitative investigation comparing the complete set of minority groups across all regional successor states from 1990 to 2017.

11. The targets included some of the most mobilized groups at the time. For example, the Tatar Autonomous Region had higher rates of disenfranchisement than most other autonomous regions (Alexopoulos, 2003: 58–59). This soon extended to the loss of many other essential citizen rights.

12. To account for changing international boundaries, this measure only considers a group’s predecessor population in the respective area it currently settles in. For example, ethnic Armenians enjoyed autonomy in the Azerbaijani part of the Soviet Union, but not in the Georgian one.

13. As minorities can by definition not experience an SDM onset if they are already engaged in one, group years with an existing SDM at time *t-1* are discarded from this first specification.

14. As we are interested in initial concessions, we exclude all years within a continuous group period (SDM or non-SDM) after which ethnic groups have already obtained a concession.

15. As only ongoing SDMs can escalate by definition, this last specification considers only years with an ongoing, but non-violent SDM at time *t-1*.
