# Supplementary material for: The Wrong Place at the Wrong Time? Territorial Autonomy and Conflict During Regime Transitions
Source: Comp Polit Stud. 2023 Apr 1;56(13):1996–2029. doi: 10.1177/00104140231168365 (PMC10584658; doi:10.1177/00104140231168365)
Supplement: Supplemental Material - The Wrong Place at the Wrong Time? Territorial Autonomy and Conflict During Regime Transitions [file sj-pdf-1-cps-10.1177_00104140231168365.pdf]

The wrong place at the wrong time? Territorial autonomy and conflict during regime transitions: Online Appendices

Appendix A: Descriptive statistics ... 2
Appendix B: Robustness checks (global sample) ... 3
Appendix B.1: Effects of autonomy, conditional on other factors ... 3
Appendix B.1.1: Combined autonomy and government inclusion ... 5
Appendix B.1.2: Autonomy and previous self-determination violence ... 7
Appendix B.1.3: Autonomy and ethno-regional organizations ... 9
Appendix B.1.4: Autonomy and economic inequality ... 11
Appendix B.1.5: Autonomy and cultural cleavages ... 12
Appendix B.1.6: Autonomy and changes in the government's support group ... 13
Appendix B.2: Probing reverse causation and endogeneity further ... 15
Appendix B.2.1: Exclusion of groups with SDMs in 3-year time period preceding last transition ... 16
Appendix B.2.2: Incorporating group-fixed effects ... 17
Appendix B.2.3: Causal sensitivity analyses ... 18
Appendix B.3: Unpacking transitions ... 19
Appendix B.3.1: Different types of regime transitions ... 20
Appendix B.3.2: Different operationalization of regime transitions and control for anocracy ... 22
Appendix B.4: Alterations to the dependent variable, sample, and specification ... 24
Appendix B.4.1: Broader dependent variable (SDM radicalization) ... 24
Appendix B.4.2: Sample alterations ... 25
Appendix B.4.3: Alternative specification ... 26
Appendix C: Territorial autonomy data (CEE-FSU sample) ... 27
Appendix C.1: Identification of territorial autonomy arrangements ... 27
Appendix C.2: Measurement of territorial autonomy indicators ... 28
Appendix C.3: Geo-coding of administrative boundaries ... 28
Appendix C.4: Aggregation onto group-level ... 29
Appendix C.5: Coding examples ... 30
Appendix C.6: Complete list of autonomy scores in our sample ... 38
Appendix D: Illustrative cases (CEE-FSU sample) ... 39
Appendix E: Robustness checks (CEE-FSU sample) ... 40
Appendix E.1: Robustness to different ‘time windows’ ... 42
Appendix E.2: Robustness to sample alterations ... 43
Appendix E.3: Robustness to additional controls ... 44
Appendix E.4: Instrumenting for autonomy with autonomy in 1989 ... 47
Appendix E.5: Probing additional assumptions ... 49
Additional literature ... 51

Appendix A: Descriptive statistics

Table A1. Descriptive statistics.

	Global sample					CEE-FSU sample				
Statistic	N	Mean	St. Dev.	Min	Max	N	Mean	St. Dev.	Min	Max
SDM onset	33,168	0.008	0.089	0	1	2,508	0.011	0.105	0	1
Concession	33,168	0.017	0.128	0	1	2,508	0.033	0.180	0	1
Violent SDM onset	33,168	0.006	0.075	0	1	2,508	0.009	0.093	0	1
Autonomy (dichotomous, based on EPR)	33,168	0.255	0.436	0	1	2,508	0.423	0.494	0	1
Autonomy (continuous, own coding)	33,168	0.204	0.269	0	1	2,508	0.333	0.311	0	1
Autonomy 1989	33,168	0.175	0.249	0	1	2,508	0.193	0.227	0	1
Recent concession	33,168	0.076	0.265	0	1	2,508	0.120	0.325	0	1
Transition	33,168	0.240	0.427	0	1	2,508	0.585	0.493	0	1
Transition (0-5)	33,168	0.367	0.482	0	1	2,508	0.760	0.427	0	1
Transition proximity	33,168	0.347	0.418	0	1	2,508	0.718	0.379	0	1
Most powerful	33,168	0.090	0.286	0	1	2,508	0.049	0.215	0	1
Included	33,168	0.302	0.459	0	1	2,508	0.127	0.333	0	1
Group size	33,168	0.108	0.164	0.0001	0.964	2,508	0.069	0.144	0.0001	0.872
Distance border	33,168	35.950	121.734	0	925	2,508	46.021	122.752	0	925
TEK irredentism	33,168	0.009	0.093	0	1	2,508	0.033	0.179	0	1
TEK SDMs	33,168	0.242	0.429	0	1	2,508	0.352	0.478	0	1
Petroleum % in settlement area	33,168	0.108	0.242	0.000	1.404	2,508	0.366	0.416	0.000	1.404
GDP p.c.	33,168	7,891.399	11,204.230	227.886	158,327.500	2,508	13,237.710	7,517.973	702.945	31,137.780
Population	33,168	141.234	297.353	0.148	1,378.665	2,508	64.816	66.426	0.332	148.689
Previous SDMs in country	33,168	2.765	4.652	0	45	2,508	10.510	12.008	0	45
Normalized polity score	33,168	0.458	0.343	0.000	1.000	2,508	0.684	0.240	0.056	1.000
Year	33,168	1,985.493	19.817	1,946	2,017	2,508	2,003.641	8.102	1,990	2,017
Ongoing SD	33,168	0.264	0.441	0	1	2,508	0.512	0.500	0	1
Years without SDM	33,168	20.542	20.408	0	71	2,508	14.656	22.379	0	71
Years without violent SDM	33,168	28.161	20.620	0	71	2,508	46.171	21.865	0	71

Appendix B: Robustness checks (global sample)

In this appendix, we probe the robustness of our first set of analyses that comprise our full global sample. We first discuss the possibility that our results may be driven by **other conditional factors** that might influence the relationship of autonomy with (violent) SDM onsets, including simultaneous government inclusion, prior violence, ethno-regional organizations, economic inequality, ethno-cultural cleavages, and changes in the government's identity (appendix B.1). Next, we probe for **left-over endogeneity and reverse causation** that might bias our findings, using more restrictive sample subsets, group-fixed effects, and a causal sensitivity analysis (appendix B.2). Third, we distinguish between **different types of transitions** and employ different operationalizations thereof (appendix B.3). Finally, we probe the **general robustness** of our findings to alterations to our dependent variable, sample composition, and specification (appendix B.4).

Appendix B.1: Effects of autonomy, conditional on other factors

In this section, we address the possibility that our attained relationship of autonomy with (violent) SDM onsets, conditional on transition periods, might, in reality, be conditioned by *other factors*, some of which might co-vary with transition periods. In what follows, we focus on six factors that previous research has shown may condition the consequences of autonomy for conflict processes.

In a first step, we **distinguish between autonomous groups that are simultaneously included in the central government and those that remain excluded from it**. One of our key arguments has been that autonomy during regime transitions increases the risks of both SDMs and their escalation because the creation of a new regime generates uncertainty and distrust among ethnic groups towards the government's future behavior. However, similar to post-conflict contexts (Cederman et al., 2015), this danger should be mitigated where autonomous groups are *themselves* part of the new regime, and possibly linked to it through co-partisanship (Bakke 2015; De la Calle 2015; Filippov et al. 2004; Mattes & Savun 2009). Such links might generate trust and foster more institutionalized bargaining between autonomous groups and the government. To account for this possibility, we replace our *autonomy* and *included* dummies with a three-way classification that accounts for their potential interactive nature. We follow Cederman and colleagues (2015) by distinguishing between *autonomy, not included*, *autonomy, included*, and *included without autonomy*. To probe whether our attained conditional relationship applies to both autonomous groups that are included and those that are excluded, we interact each of these dummy variables with our transition measures (appendix B.1.1).

Our findings indicate that the positive association of autonomy with (violent) SDM onsets during transition periods **is limited to the majority of constellations where autonomy is awarded to excluded groups** (~74% of autonomous group years in our sample). Conversely, we attain no similar relationship for the rarer constellation where a group is, in addition to possessing autonomy, simultaneously included in the central government. This finding points to an important, politically relevant, remedy for mitigating the potentially destabilizing consequences of autonomy in transition periods: to avoid potentially escalating self-determination claims during regime transitions, autonomous ethnic groups should be incorporated in the central government. This may build mutual trust and foster more predictable, institutionalized bargaining (see conclusion of the main article).

In a second step, we **distinguish between autonomous groups that have previously engaged in self-determination violence and those that have never been involved in such violence**. As highlighted in numerous important contributions (e.g., Anderson 2014; Grigoryan 2012; McGarry & O'Leary 2009), autonomy provided *after* violence has already erupted may be 'too little, too late' and fail to appease its beneficiaries (Cederman et al. 2015). In particular, groups suffering from prior targeted violence may be more likely to assess that the central government will not protect their life chances in the future (Hale 2008, pp. 80-81). To account for this possibility, we split up our autonomy dummy into two dichotomous variables, depending on whether a group has previously engaged in violent SDMs up to the preceding year (t-1): *autonomy, no previous violence* and *autonomy, previous violence*.[1] To probe whether our attained conditional relationship holds in both cases, we again interact both of these dichotomous variables with our transition measures. We also incorporate a dummy variable for *previous violence* and interact it with our transition measures. Thereby, we account for potential baseline differences between groups with and without previous violence, during both transition and other periods.

The results from these analyses (appendix B.1.2) provide more nuance. Our **findings hold fully for autonomous groups that have not been involved in prior self-determination violence**, though they are less pronounced for violent SDM onsets. When focusing on the smaller number of cases (22% of autonomous group years in our sample) where autonomy was provided to **groups with prior self-determination violence, our findings hold for violent SDM onsets and are similar in direction and magnitude yet fail to attain statistical significance for SDM onsets**. Overall, this reassures us that our findings are not explained 'away' by this factor.

In a third step, we account for three **additional conditional relationships** of autonomy with (violent) SDM onsets. We do so by incorporating control variables for three important factors and interacting them with both autonomy and our transition measures. Following previous scholarship, we focus on three factors:

[1] For this purpose, we identify all groups that previously engaged in violence SDMs up to the previous year (t-1), according to our measures.

- First (appendix B.1.3), we account for the existence and influence of **ethno-regional organizations**. These constitute a critical link between autonomy and ethnically-based claims and violence (Brancati 2006) and may generate destabilizing outbidding processes (Vogt et al. 2021). Thereby, they may make autonomous groups more likely to initiate and violently escalate self-determination movements. In one check that comprises our full sample, we proxy for the influence of ethno-regional organizations with the combined vote share of all ethnic and regional parties in the last national election, according to the V-Parties dataset (Lindberg et al. 2022). In another check, limited to 37 countries in the EPR Organizations Dataset (EPR-O, Vogt et al. 2021), we proxy for it with a group-specific variable based on EPR-O. This counts the (logged) number of political organizations that are linked to each ethnic group in each year through ethnic claims, recruitment, or electoral support.
- Second (appendix B.1.4), we account for **inter-ethnic economic inequalities**. For both relatively poorer and relatively wealthier groups, autonomy may make the onset and violent escalation of SDMs more likely; conversely, for groups of average wealth, autonomy may be more likely to exert stabilizing effects (see Bakke 2015; Deiwiks et al. 2012). We account for this factor with two variables that capture economic disparities for groups that are relatively poorer (*low ratio*) and relatively wealthier (*high ratio*) than other groups in the same country, respectively. These variables are constructed equivalently to Cederman et al. (2011), based on the spatial wealth measures provided by Nordhaus (2006).[2]
- Third (appendix B.1.5), we account for the strength of **cultural cleavages** demarcating a group from other groups. Culturally dissimilar groups may have a stronger degree of ethnic identification (Hale 2008), be more likely to engage in conflict (cf. Bormann et al. 2017), and may thereby also more likely to initiate and escalate SDMs. At the same time, they may also be more likely to be pacified by autonomy, particularly if autonomy includes a cultural component (Bakke 2015). To account for this factor, we rely on data from the EPR-Ethnic Dimensions Dataset (Vogt et al. 2015) and use information on group-wise religious practices and spoken languages. We construct a variable for mean cultural cleavages, given by the average of religious and linguistic cleavages dividing a group from all other groups in the same country, similar to the procedures used by Bormann et al. (2017).

Our findings indicate that **groups with a higher number of ethnic organizations, that are relatively poorer, and that are culturally more dissimilar are more likely to initiate (violent) SDMs.** However, in our approach, we find no pronounced interactive effects between these factors, autonomy, and transition periods. Moreover, our **original findings remain substantively unchanged**, thereby reassuring us that they are indeed due to the conditioning role of transition periods, and not due to these other factors.

In a fourth step, we account for the possibility that our findings could be driven by **shifts in the government's identity** more broadly, beyond transition contexts, for example owing to elections that bring parties to power that ideologically break with the previous government. Similar to transitions, such shifts could generate uncertainty among autonomous groups and incentivize them to make self-determination demands while the new government is not yet consolidated. To account for such shifts, we rely on the V-Parties dataset (Lindberg et al. 2022) to identify the support group(s) of all governing parties.[3] We code government support group changes in all years where the composition of governing parties' support groups changes. Analogously to our measures for regime transition, we capture such shifts using both dichotomous (change in government support group in the last five years) and continuous variables (proximity to government support group change, measured as a decaying shock).[4] We incorporate these variables as controls in our main models and interact them with *autonomy* to probe how they affect (violent) SDM onsets and whether our original findings remain robust (appendix B.1.6). As this information is generally only available for the post-1970 period, our sample is more limited in these analyses.

In line with the above reasoning, we find that autonomous groups are indeed **more likely to initiate SDMs during periods in which the government's support groups change**. This suggests that, similar to transitions, such shifts may generate uncertainty and motivate groups to seek greater self-determination. However, we do not find that such shifts, which constitute more limited breaks with past power constellations than profound regime changes, also lead autonomous groups to violently escalate SDMs. Moreover, our **interactions between autonomy and regime transitions remain positive and significant**, even when controlling for such shifts that may *themselves* be related to transitions. However, our results provide more nuance: We find that the effect of autonomy on (violent) SDM onsets is stronger where regime transitions coincide with shifts in ruling parties' support group(s). Moreover, for violent SDM onsets, we find that the effect of autonomy is only statistically significant in such cases. This may reflect the greater uncertainty during regime periods that coincide with large ideological shifts, as opposed to regime transitions that see no changes in the government's support group(s).

[2] Regrettably, these data were only available for the years 1990, 1995, 2000, and 2005. For years in-between, they were linearly interpolated; for years before and after, they were extrapolated.

[3] This provides information on whether each party's support groups include the aristocracy, agrarian elites, business elites, the military, an ethnic or racial group, a religious group, local elites, urban working classes, urban middle classes, rural working classes, rural middle classes, regional groups, or women.

[4] We again implement this by coding a variable regime *gov. support change proximity* which we code as 1 during years that see a change in the government's support group, with this value decaying in subsequent years with a three-year half-life.

Appendix B.1.1: Combined autonomy and government inclusion

Table A2. Results: combined autonomy and government inclusion.

	**SDM onset**	**Violent SDM onset**	**SDM onset**	**Violent SDM onset**
	**Model 1**	**Model 2**	**Model 3**	**Model 4**
Autonomy, not included	-0.375	-0.673	-0.321	-0.740
	(0.478)	(0.412)	(0.485)	(0.456)
Autonomy, included	0.132	0.291	0.223	0.241
	(0.652)	(0.490)	(0.645)	(0.547)
Included without autonomy	-0.075	-0.834*	-0.093	-0.860*
	(0.435)	(0.480)	(0.461)	(0.508)
Transition (0-5)	0.659***	-0.036		
	(0.251)	(0.248)		
Autonomy, not included x transition (0-5)	1.780***	1.463***		
	(0.539)	(0.496)		
Autonomy, included x transition (0-5)	-0.672	-0.291		
	(0.612)	(0.596)		
Included without autonomy x transition (0-5)	0.100	-0.109		
	(0.477)	(0.632)		
Transition proximity			0.894***	0.045
			(0.288)	(0.291)
Autonomy, not included x transition proximity			1.655***	1.637***
			(0.585)	(0.606)
Autonomy, included x transition proximity			-0.861	-0.202
			(0.834)	(0.766)
Included without autonomy x transition proximity			0.105	-0.065
			(0.570)	(0.715)
Constant	-2.738**	-1.248	-2.927***	-1.246
	(1.109)	(1.119)	(1.131)	(1.148)
Wald $\chi 2$				
$\beta_{\text{Autonomy, not included x transition (0-5)}} = 0$	10.884***	8.7135**		
$\beta_{\text{Autonomy, included x transition (0-5)}} = 0$	1.2062	0.2383		
$\beta_{\text{Autonomy, not included}} + \beta_{\text{Autonomy, not included x transition (0-5)}} = 0$	24.234***	6.3393*		
$\beta_{\text{Autonomy, included}} + \beta_{\text{Autonomy, included x transition (0-5)}} = 0$	0.4442	0		
$\beta_{\text{Autonomy, not included x transition proximity}} = 0$			8.0138**	7.3055**
$\beta_{\text{Autonomy, included x transition proximity}} = 0$			1.0641	0.0696
$\beta_{\text{Autonomy, not included}} + \beta_{\text{Autonomy, not included x transition proximity}} = 0$			18.751**	6.5471*
$\beta_{\text{Autonomy, included}} + \beta_{\text{Autonomy, included x transition proximity}} = 0$			0.4287	0.0101
N	24466	31044	24466	31044
Log Likelihood	-1162.623	-861.226	-1158.804	-860.379
AIC	2519.246	1918.451	2511.609	1916.758

***p < .01; **p < .05; *p < .1; Country-clustered errors in parentheses. Cubic term for time dependence of dependent variable, control variables, and year- and region-fixed effects included but not reported.

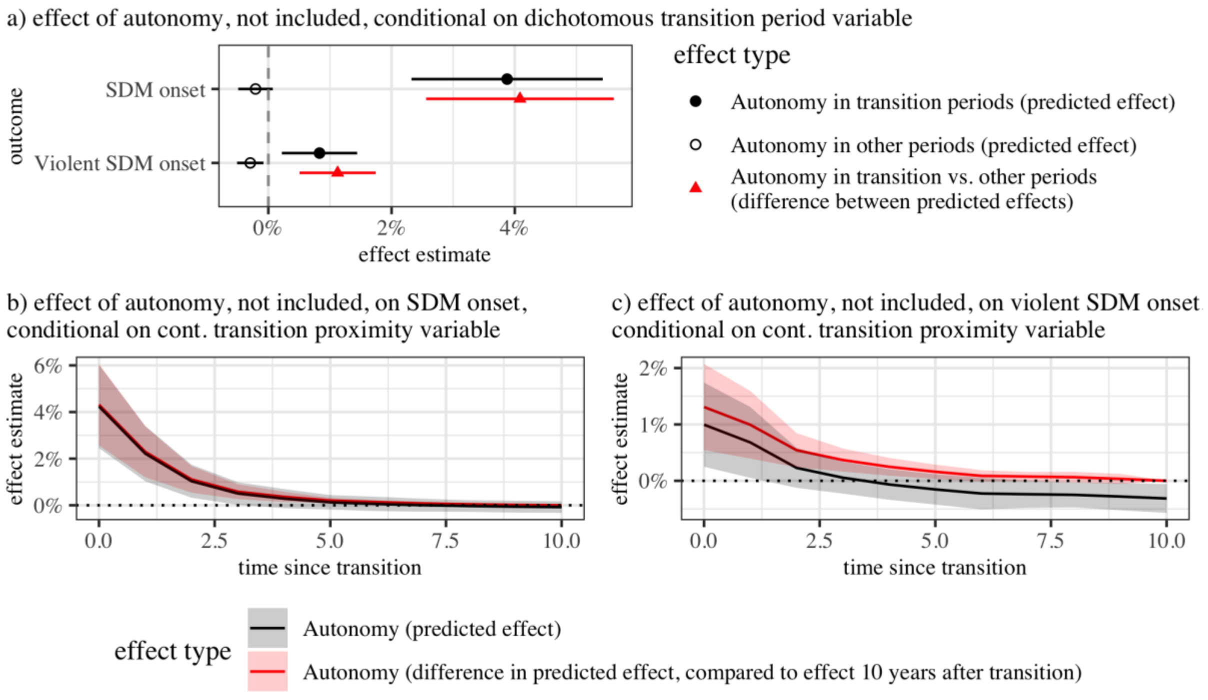

Figure A1. First and second-differences in the predicted probability of (violent) SDM onset, depending on autonomy, not included, and transition periods. Based on models 1-4 in table A2.

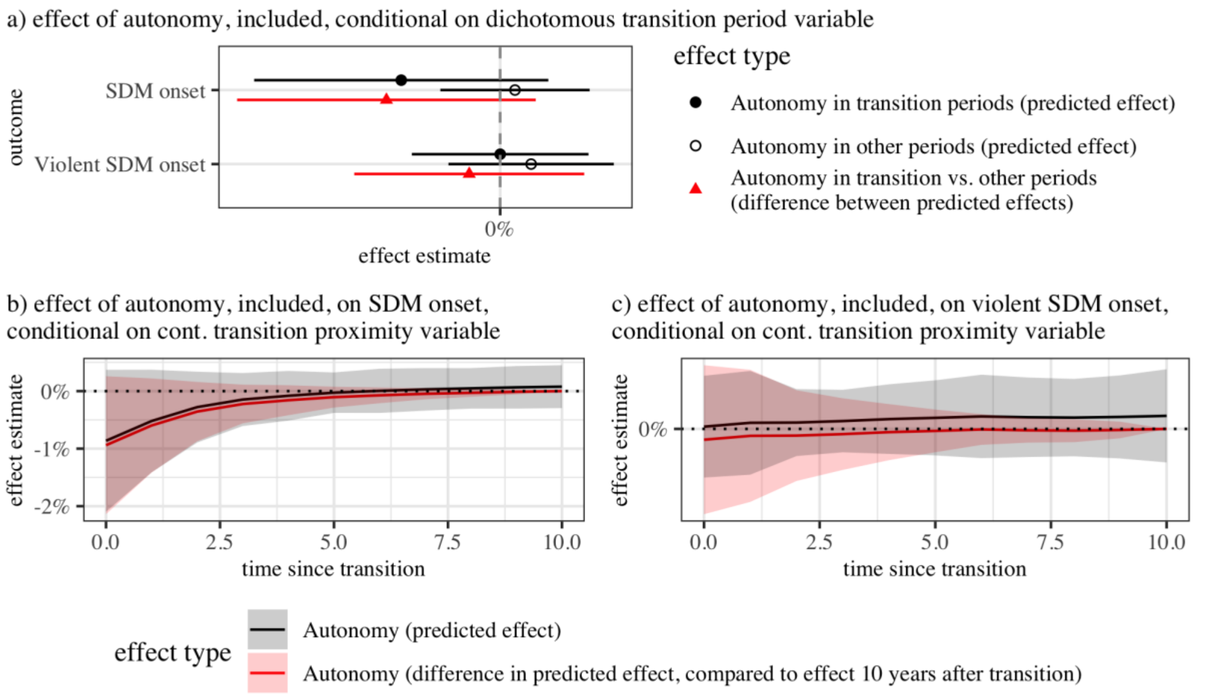

Figure A2. First and second-differences in the predicted probability of (violent) SDM onset, depending on autonomy, included, and transition periods. Based on models 1-4 in table A2.

Appendix B.1.2: Autonomy and previous self-determination violence

Table A3. Results: autonomy and previous self-determination violence.

	**SDM onset**	**Violent SDM onset**	**SDM onset**	**Violent SDM onset**
	**Model 1**	**Model 2**	**Model 3**	**Model 4**
Autonomy, no previous violence	-0.176	-0.174	-0.174	-0.143
	(0.445)	(0.563)	(0.458)	(0.593)
Autonomy, previous violence	0.097	-0.651	0.110	-0.864*
	(0.712)	(0.425)	(0.761)	(0.471)
Previous violence	1.256**	0.529	1.274**	0.701*
	(0.490)	(0.401)	(0.545)	(0.416)
Transition (0-5)	0.715***	0.209		
	(0.241)	(0.283)		
Autonomy, no previous violence x transition (0-5)	1.142**	0.956		
	(0.533)	(0.597)		
Autonomy, previous violence x transition (0-5)	1.480	1.617**		
	(1.108)	(0.658)		
Previous violence x transition (0-5)	-0.369	-0.883		
	(0.686)	(0.635)		
Transition proximity			0.954***	0.437
			(0.263)	(0.331)
Autonomy, no previous violence x transition proximity			1.148**	0.947
			(0.563)	(0.702)
Autonomy, previous violence x transition proximity			1.387	2.188***
			(1.365)	(0.776)
Previous violence x transition proximity			-0.408	-1.359*
			(0.966)	(0.734)
Constant	-2.891***	-1.029	-2.994***	-1.025
	(1.044)	(1.111)	(1.058)	(1.150)
Wald $\chi 2$				
$\beta_{\text{Autonomy, no previous violence x transition (0-5)}} = 0$	4.5925*	2.5652		
$\beta_{\text{Autonomy, previous violence x transition (0-5)}} = 0$	1.7848	6.0376*		
$\beta_{\text{Autonomy, no previous violence}} + \beta_{\text{Autonomy, no previous violence x transition (0-5)}} = 0$	6.886***	6.3812*		
$\beta_{\text{Autonomy, previous violence}} + \beta_{\text{Autonomy, previous violence x transition (0-5)}} = 0$	4.9335*	4.7128*		
$\beta_{\text{Autonomy, no previous violence x transition proximity}} = 0$			4.1583*	1.8177
$\beta_{\text{Autonomy, previous violence x transition proximity}} = 0$			1.0327	7.9375**
$\beta_{\text{Autonomy, no previous violence}} + \beta_{\text{Autonomy, no previous violence x transition proximity}} = 0$			6.6116*	5.5453*
$\beta_{\text{Autonomy, previous violence}} + \beta_{\text{Autonomy, previous violence x transition proximity}} = 0$			2.7944	6.4697*
N	24466	31044	24466	31044
Log Likelihood	-1161.996	-864.026	-1156.314	-860.942
AIC	2519.993	1926.051	2508.628	1919.883

***p < .01; **p < .05; *p < .1; Country-clustered errors in parentheses. Cubic term for time dependence of dependent variable, control variables, and year- and region-fixed effects included but not reported.

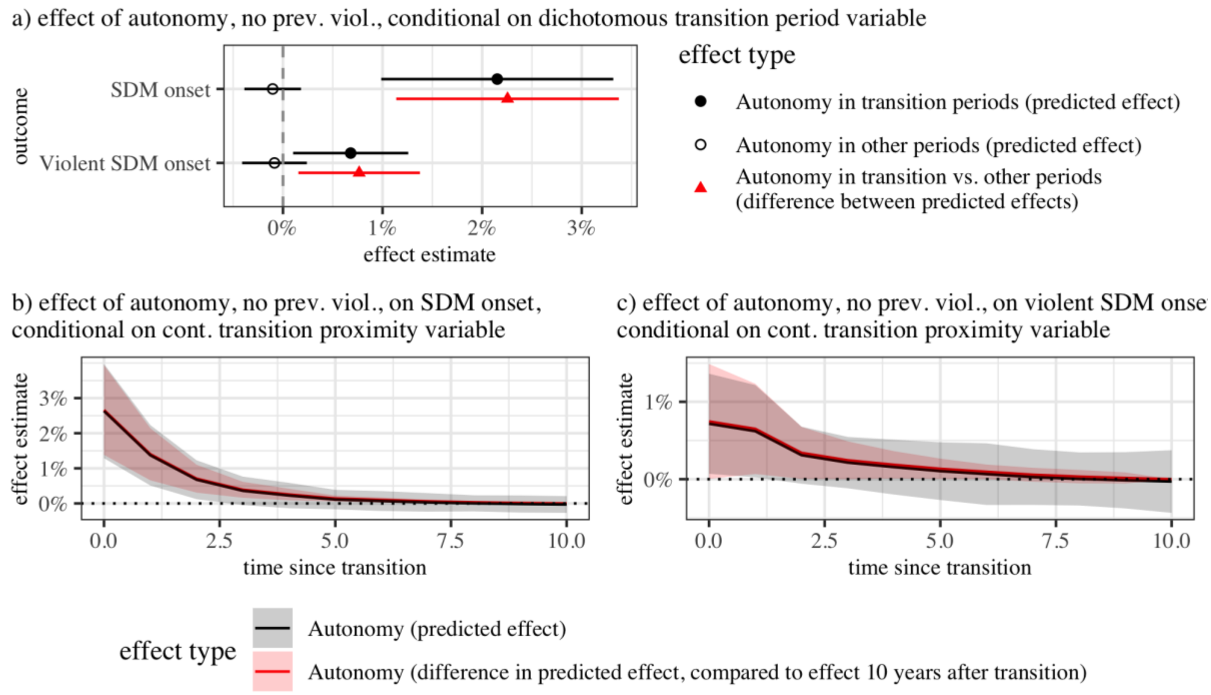

Figure A3. First and second-differences in the predicted probability of (violent) SDM onset, depending on autonomy, no previous violence, and transition periods. Based on models 1-4 in table A3.

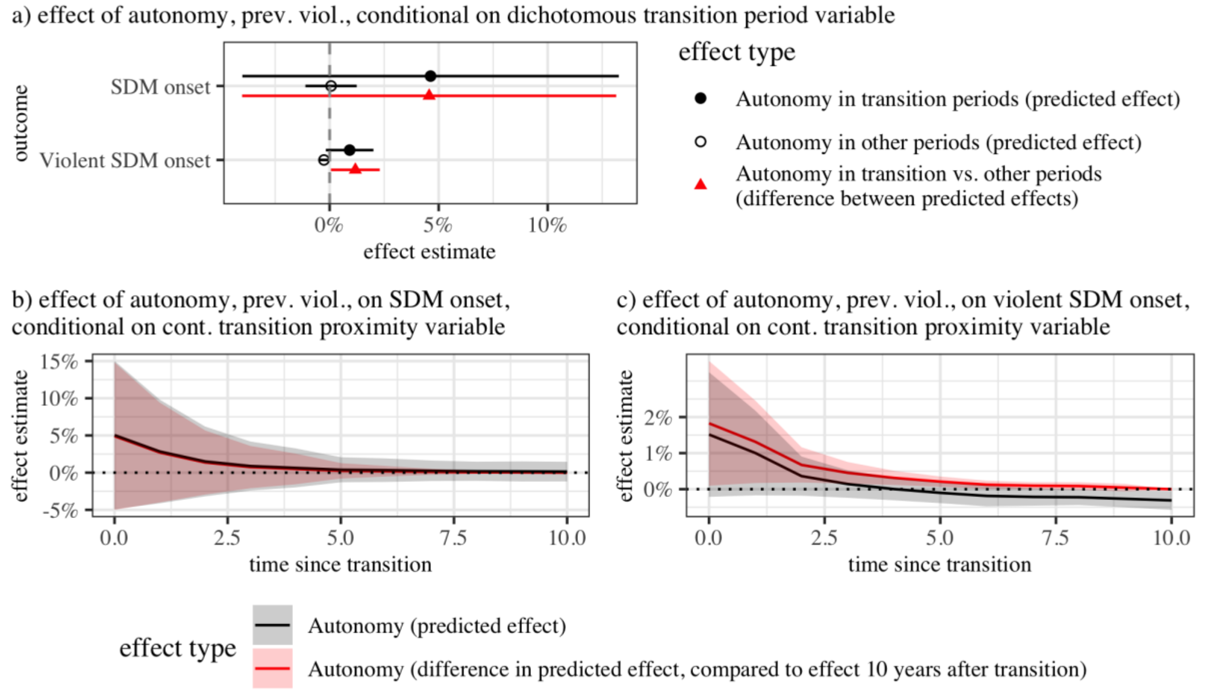

Figure A4. First and second-differences in the predicted probability of (violent) SDM onset, depending on autonomy, previous violence, and transition periods. Based on models 1-4 in table A3.

Appendix B.1.3: Autonomy and ethno-regional organizations

Table A4. Results: autonomy and ethno-regional organizations (first operationalization using % of total vote share of ethno-regional parties in last national election, full sample).

	**SDM onset Model 1**	**Violent SDM onset Model 2**	**SDM onset Model 3**	**Violent SDM onset Model 4**
Autonomy	-0.032	-0.332	-0.026	-0.394
	(0.416)	(0.388)	(0.429)	(0.421)
Transition (0-5)	0.718***	-0.021		
	(0.246)	(0.226)		
Autonomy x transition (0-5)	1.184**	1.108***		
	(0.516)	(0.404)		
Transition proximity			0.946***	0.119
			(0.283)	(0.271)
Autonomy x transition proximity			1.178**	1.259***
			(0.558)	(0.488)
Regional party vote share	0.0004	0.0001	0.0001	0.001
	(0.006)	(0.004)	(0.007)	(0.004)
Autonomy x regional party vote share	-0.017	-0.0002	-0.017	0.0005
	(0.014)	(0.009)	(0.014)	(0.009)
Transition (0-5) x regional party vote share	-0.004	-0.003		
	(0.007)	(0.005)		
Transition proximity x regional party vote share			-0.004	-0.007
			(0.008)	(0.006)
Constant	-3.157***	-1.163	-3.268***	-1.149
	(1.103)	(1.143)	(1.125)	(1.194)
Wald $\chi 2$				
$\beta_{\text{Autonomy x transition (0-5)}} = 0$	5.275*	7.5141**		
$\beta_{\text{Autonomy}} + \beta_{\text{Autonomy x transition (0-5)}} = 0$	10.065**	7.5616**		
$\beta_{\text{Autonomy x transition proximity}} = 0$			4.4604*	6.6578**
$\beta_{\text{Autonomy}} + \beta_{\text{Autonomy x transition proximity}} = 0$			8.8155**	8.3116**
N	24466	31044	24466	31044
Log Likelihood	-1169.156	-866.410	-1163.682	-864.581
AIC	2532.311	1928.819	2521.365	1925.162

***p < .01; **p < .05; *p < .1; Country-clustered errors in parentheses. Cubic term for time dependence of dependent variable, control variables, and year- and region-fixed effects included but not reported.

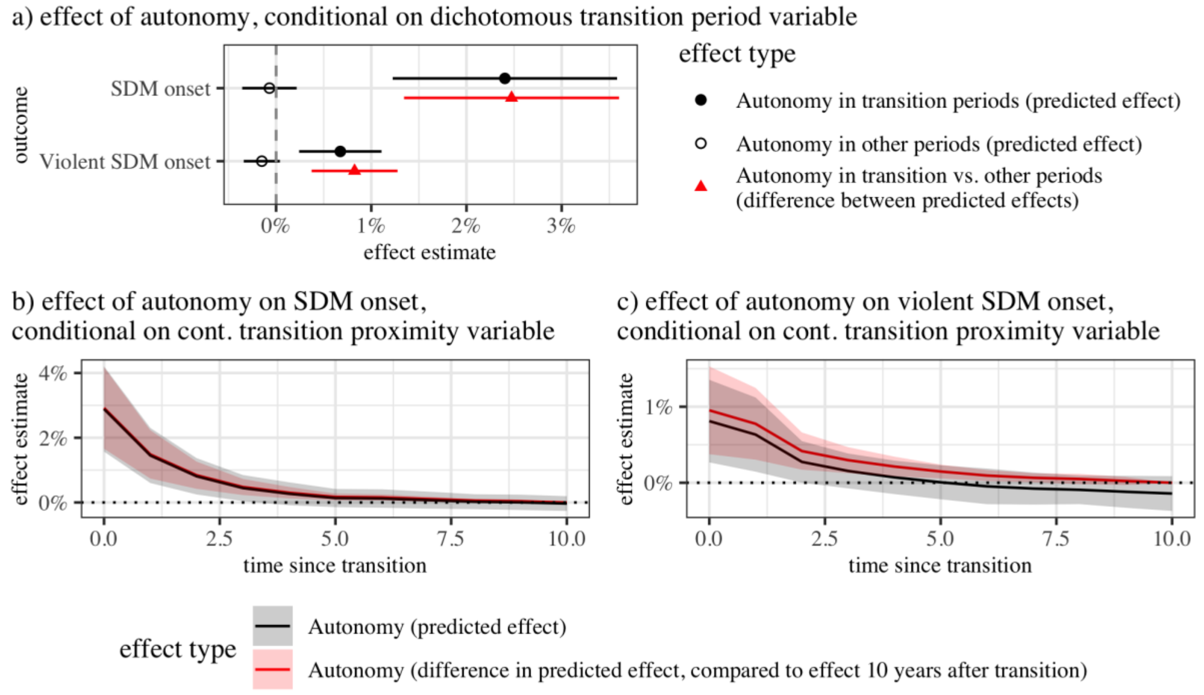

Figure A5. First and second-differences in the predicted probability of (violent) SDM onset, depending on autonomy and transition periods. Based on models 1-4 in table A4.

Table A5. Results: autonomy and ethno-regional organizations (second operationalization with logged number of group-wise ethnic organizations, for all countries in EPR-Organizations dataset, 1946-2013).

	**SDM onset Model 1**	**Violent SDM onset Model 2**	**SDM onset Model 3**	**Violent SDM onset Model 4**
Autonomy	-0.409	0.338	-0.354	0.191
	(0.502)	(1.061)	(0.506)	(1.070)
Transition (0-5)	1.445*	1.348***		
	(0.836)	(0.417)		
Autonomy x transition (0-5)	2.630***	0.730		
	(0.550)	(0.817)		
Transition proximity			1.718*	1.288**
			(0.901)	(0.532)
Autonomy x transition proximity			2.562***	1.110
			(0.540)	(0.959)
No. ethnic organizations (log)	1.181***	1.566***	1.382***	1.569***
	(0.372)	(0.463)	(0.407)	(0.456)
Autonomy x no. ethnic organizations (log)	0.240	-0.883*	0.222	-0.868
	(0.578)	(0.536)	(0.525)	(0.537)
Transition (0-5) x no. ethnic organizations (log)	-0.279	-0.575**		
	(0.669)	(0.243)		
Transition proximity x no. ethnic organizations (log)			-0.695	-0.612**
			(0.759)	(0.280)
Constant	-1.090	0.637	-1.645	0.652
	(2.364)	(3.014)	(2.526)	(2.984)
Wald $\chi 2$				
$\beta_{\text{Autonomy x transition (0-5)}} = 0$	22.871***	0.7994		
$\beta_{\text{Autonomy}} + \beta_{\text{Autonomy x transition (0-5)}} = 0$	18.968***	3.4619		
$\beta_{\text{Autonomy x transition proximity}} = 0$			22.533***	1.3395
$\beta_{\text{Autonomy}} + \beta_{\text{Autonomy x transition proximity}} = 0$			18.46***	5.2398*
N	7763	9942	7763	9942
Log Likelihood	-294.265	-234.313	-294.835	-234.116
AIC	772.530	654.626	773.670	654.231

***p < .01; **p < .05; *p < .1; Country-clustered errors in parentheses. Cubic term for time dependence of dependent variable, control variables, and year- and region-fixed effects included but not reported.

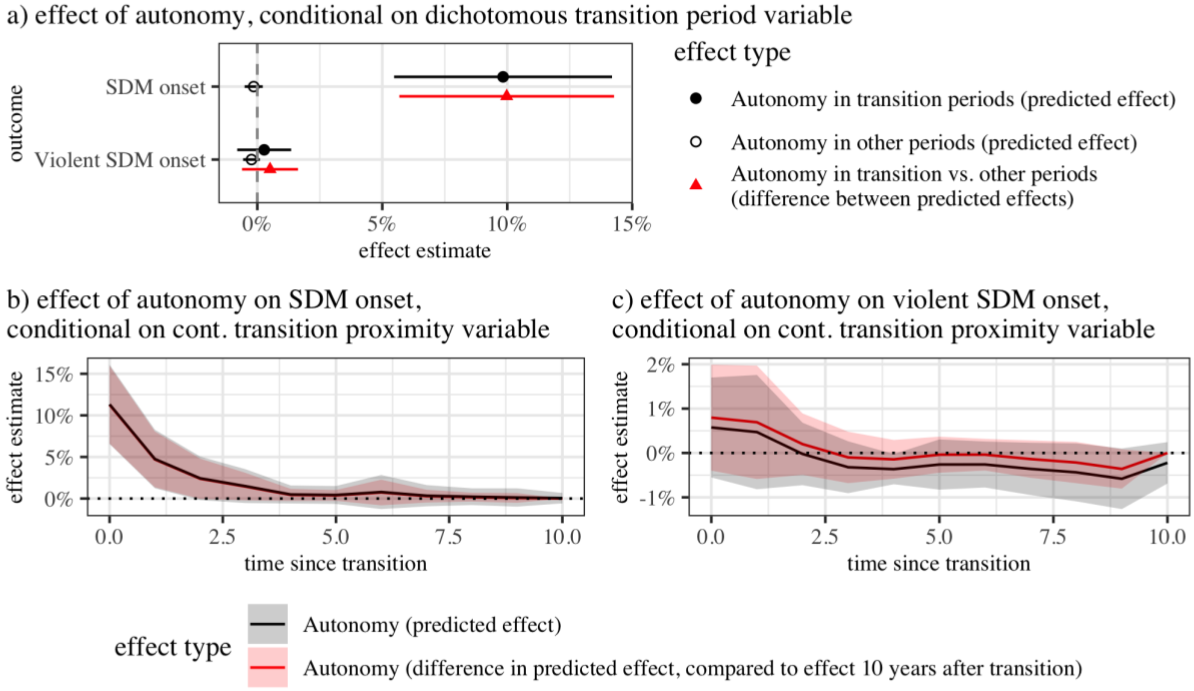

Figure A6. First and second-differences in the predicted probability of (violent) SDM onset, depending on autonomy and transition periods. Based on models 1-4 in table A5.

Appendix B.1.4: Autonomy and economic inequality

Table A6. Results: autonomy and economic inequality.

	**SDM onset**	**Violent SDM onset**	**SDM onset**	**Violent SDM onset**
	**Model 1**	**Model 2**	**Model 3**	**Model 4**
Autonomy	-0.197	-0.514	-0.201	-0.601
	(0.394)	(0.355)	(0.406)	(0.388)
Transition (0-5)	0.763***	0.094		
	(0.228)	(0.263)		
Autonomy x transition (0-5)	1.082**	1.226***		
	(0.517)	(0.402)		
Transition proximity			0.998***	0.213
			(0.256)	(0.307)
Autonomy x transition proximity			1.096**	1.422***
			(0.557)	(0.479)
Autonomy x low ratio	-0.002	0.003	-0.003	0.003
	(0.019)	(0.007)	(0.019)	(0.007)
Transition (0-5) x low ratio	-0.001	-0.019***		
	(0.017)	(0.007)		
Transition proximity x low ratio			-0.001	-0.019**
			(0.019)	(0.009)
Autonomy x high ratio	0.239	0.262	0.239	0.288
	(0.189)	(0.314)	(0.190)	(0.305)
Transition (0-5) x high ratio	-0.144	-0.321		
	(0.136)	(0.228)		
Transition proximity x high ratio			-0.147	-0.413
			(0.131)	(0.315)
Low ratio	0.021***	0.014*	0.021***	0.014*
	(0.007)	(0.008)	(0.008)	(0.008)
High ratio	-0.078	-0.024	-0.078	0.003
	(0.136)	(0.176)	(0.133)	(0.190)
Constant	-3.060***	-0.997	-3.156***	-1.003
	(1.094)	(1.168)	(1.101)	(1.243)
Wald $\chi 2$				
$\beta_{\text{Autonomy x transition (0-5)}} = 0$	4.3841*	9.2962**		
$\beta_{\text{Autonomy}} + \beta_{\text{Autonomy x transition (0-5)}} = 0$	4.6528*	5.4175*		
$\beta_{\text{Autonomy x transition proximity}} = 0$			3.8722*	8.7998**
$\beta_{\text{Autonomy}} + \beta_{\text{Autonomy x transition proximity}} = 0$			4.4062*	6.2954*
N	24466	31044	24466	31044
Log Likelihood	-1166.378	-864.631	-1160.572	-861.573
AIC	2532.756	1929.262	2521.145	1925.147

$^{***}p < .01$; $^{**}p < .05$; $^{*}p < .1$; Country-clustered errors in parentheses. Cubic term for time dependence of dependent variable, control variables, and year- and region-fixed effects included but not reported.

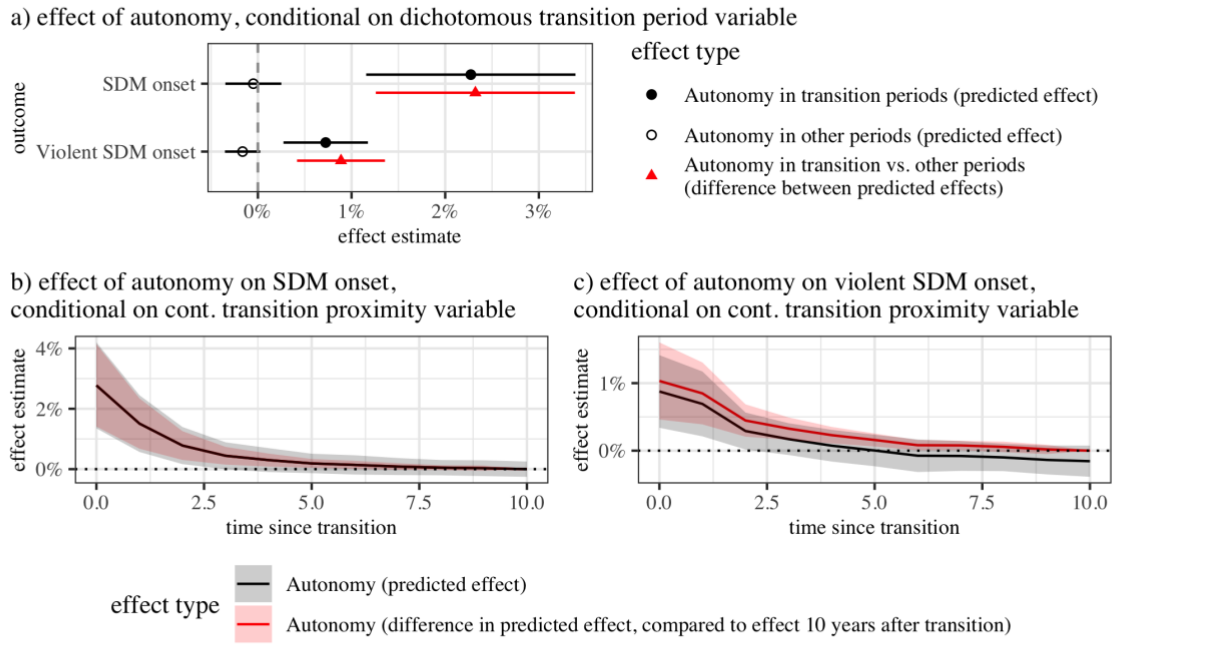

Figure A7. First and second-differences in the predicted probability of (violent) SDM onset, depending on autonomy and transition periods. Based on models 1-4 in table A6.

Appendix B.1.5: Autonomy and cultural cleavages

Table A7. Results: Autonomy and cultural (religious and linguistic) cleavages.

	SDM onset	Violent SDM onset	SDM onset	Violent SDM onset
	**Model 1**	**Model 2**	**Model 3**	**Model 4**
Autonomy	0.607	0.035	0.719	0.125
	(0.779)	(0.725)	(0.781)	(0.735)
Transition (0-5)	0.076	0.209		
	(0.545)	(0.600)		
Autonomy x transition (0-5)	1.195**	1.141***		
	(0.472)	(0.425)		
Transition proximity			0.284	0.117
			(0.620)	(0.678)
Autonomy x transition proximity			1.203**	1.305**
			(0.519)	(0.518)
Autonomy x cultural cleavages	-1.237	-0.580	-1.419	-0.816
	(1.052)	(1.229)	(1.045)	(1.292)
Transition (0-5) x cultural cleavages	0.976	-0.432		
	(0.813)	(0.796)		
Transition proximity x cultural cleavages			1.013	-0.129
			(0.873)	(0.912)
Cultural cleavages	1.405*	1.217*	1.432*	1.093
	(0.788)	(0.690)	(0.784)	(0.699)
Constant	-3.885***	-2.165	-4.001***	-2.080
	(1.151)	(1.346)	(1.171)	(1.414)
Wald $\chi 2$				
$\beta_{\text{Autonomy x transition (0-5)}} = 0$	6.4041*	7.1935**		
$\beta_{\text{Autonomy}} + \beta_{\text{Autonomy x transition (0-5)}} = 0$	4.1762*	1.775		
$\beta_{\text{Autonomy x transition proximity}} = 0$			5.3742*	6.3479*
$\beta_{\text{Autonomy}} + \beta_{\text{Autonomy x transition proximity}} = 0$			4.635*	2.2859
N	24444	30991	24444	30991
Log Likelihood	-1163.372	-855.151	-1157.649	-853.832
AIC	2520.744	1906.301	2509.297	1903.664

***p < .01; **p < .05; *p < .1; Country-clustered errors in parentheses. Cubic term for time dependence of dependent variable, control variables, and year- and region-fixed effects included but not reported.

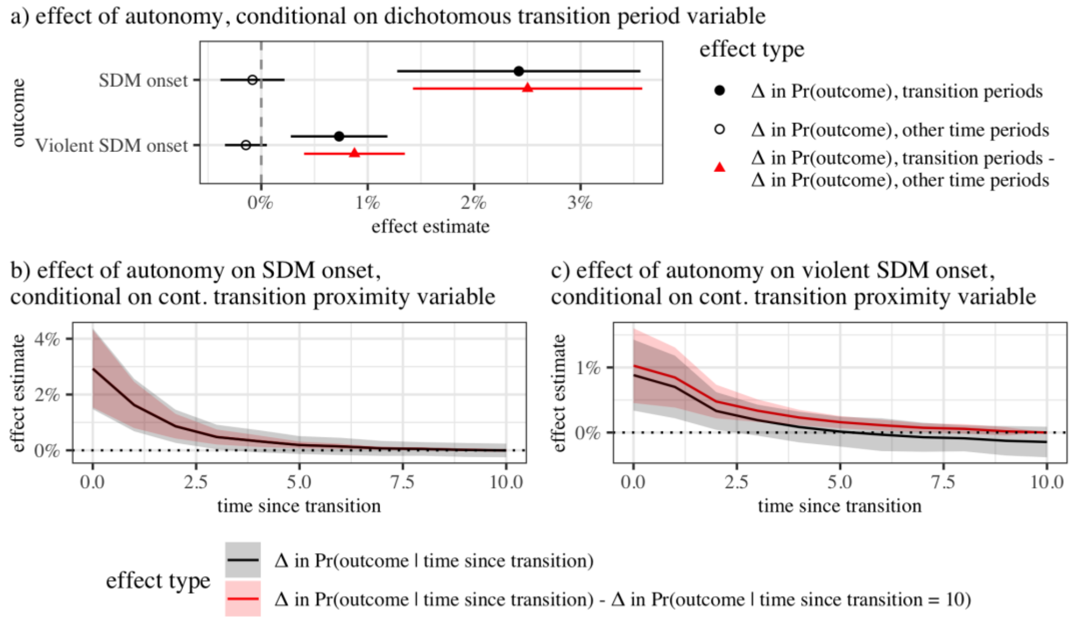

Figure A8. First and second-differences in the predicted probability of (violent) SDM onset, depending on autonomy and transition periods. Based on models 1-4 in table A7.

Appendix B.1.6: Autonomy and changes in the government's support group

Table A8. Results: autonomy and changes in the government's support group.

	SDM onset Model 1	Violent SDM onset Model 2	SDM onset Model 3	Violent SDM onset Model 4
Autonomy	-0.191	-0.907***	-0.336	-1.104***
	(0.419)	(0.331)	(0.422)	(0.366)
Transition (0-5)	0.849**	0.142		
	(0.343)	(0.288)		
Autonomy x transition (0-5)	1.408**	1.086**		
	(0.565)	(0.492)		
Transition proximity			1.081***	0.001
			(0.383)	(0.340)
Autonomy x transition proximity			1.529***	1.458***
			(0.587)	(0.550)
Gov. support change (0-5)	0.527	-0.353		
	(0.426)	(0.341)		
Autonomy x gov. support change (0-5)	1.753**	0.567		
	(0.700)	(0.489)		
Gov. support change proximity			1.016*	-0.212
			(0.597)	(0.464)
Autonomy x gov. support change proximity			2.617***	0.889
			(0.879)	(0.574)
Constant	-9.389***	-21.337***	-8.595***	-21.452***
	(2.933)	(1.773)	(2.704)	(1.554)
Wald $\chi 2$				
$\beta_{\text{Autonomy x transition (0-5)}} = 0$	6.2111*	4.8743*		
$\beta_{\text{Autonomy}} + \beta_{\text{Autonomy x transition (0-5)}} = 0$	13.187***	0.1955		
$\beta_{\text{Autonomy x transition proximity}} = 0$			6.7834**	7.0292**
$\beta_{\text{Autonomy}} + \beta_{\text{Autonomy x transition proximity}} = 0$			11.691***	0.6974
N	14943	20192	14943	20192
Log Likelihood	-552.451	-507.971	-545.166	-507.206
AIC	1246.902	1159.942	1232.331	1158.412

***p < .01; **p < .05; *p < .1; Country-clustered errors in parentheses. Cubic term for time dependence of dependent variable, control variables, and year- and region-fixed effects included but not reported.

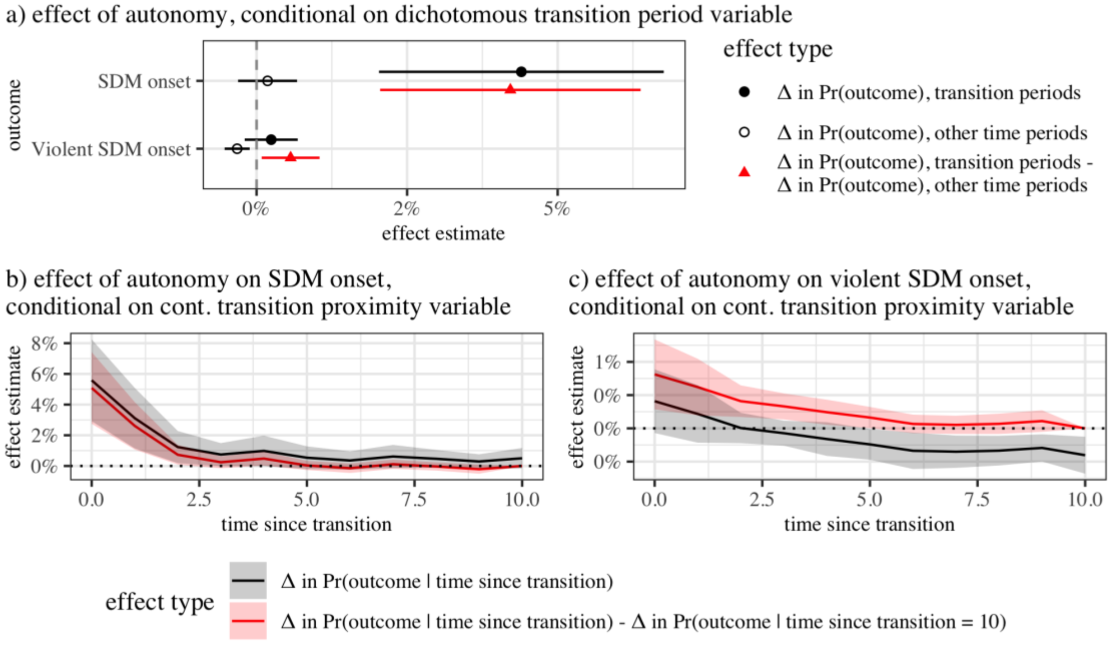

Figure A9. First and second-differences in the predicted probability of (violent) SDM onset, depending on autonomy and transition periods. Based on models 1-4 in table A8.

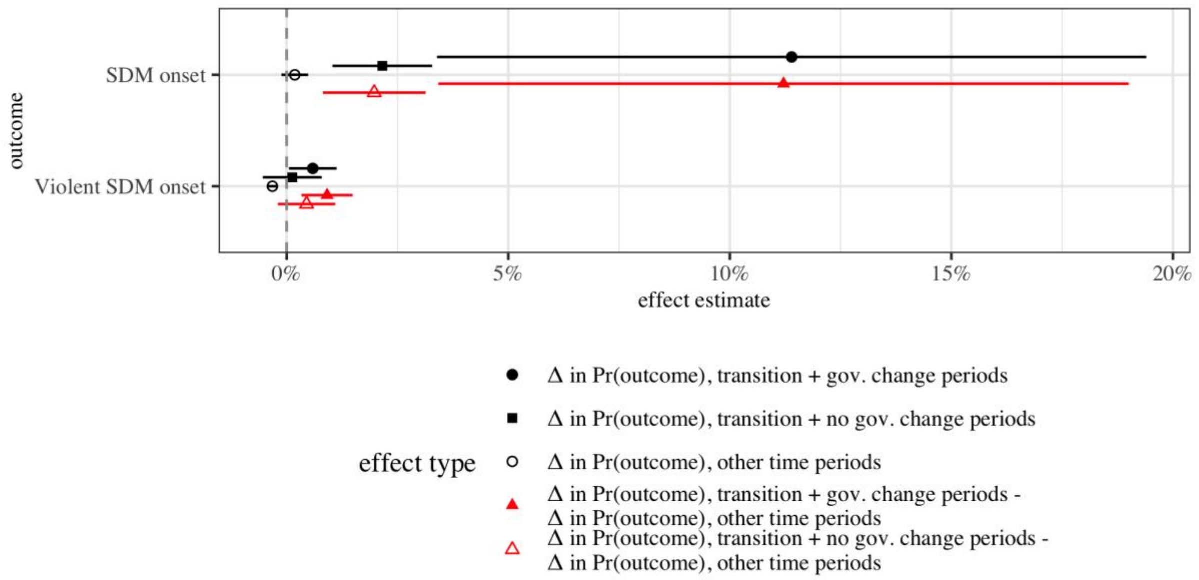

Figure A10. First and second-differences in the predicted probability of (violent) SDM onset, depending on autonomy, transition periods, and periods of changing government support groups. Based on models 1-4 in table A8.

Appendix B.2: Probing reverse causation and endogeneity further

In this section, we probe reverse causation and endogeneity concerns further. In a first step, we re-run our models with our dichotomous transition variables (models 1 and 3 in table 2), while **excluding any group from our sample that was engaged in a (violent) SDM within three years before the last regime transition**. We first do so for our full sample, while retaining our interaction between *autonomy* and *transition (0-5)*. Next, we repeat this procedure, while limiting the sample to the subset of group years during transition periods, in which we exclude our interaction term. Together, these analyses enable us to further reduce the risk of mobilization-induced autonomy (figure 1, challenge 2b), whereby autonomy may be given to mobilized groups in a bid to stave off looming transitions. Reassuringly, our results remain substantively similar to our main results in both procedures.

In a second step, we incorporate **group-fixed effects**, while switching to a linear probability model. Thereby, we limit our analysis to *over-time variation* in autonomy provided to each group. Conversely, we exclude variation between countries and groups that may be more prone to bias arising from endogenous autonomy (figure 1, challenge 2a). Thereby, autonomy might be predominantly used by states that are most prone to self-determination challenges; moreover, autonomy might be preferentially awarded to those groups with the highest threat capabilities in the first place. In these procedures, we discard our control variables except *government inclusion* and *ongoing SDM* in or models of violent SDM onset due to multicollinearity. Reassuringly, while weaker in magnitude and in statistical significance due to the much reduced variation analyzed in these procedures, our interaction terms between autonomy and transition periods retain positive sign and remain substantial in magnitude. Most reassuringly, our results for violent SDM onsets, which may be most prone to endogenous autonomy concerns, remain statistically significant at the 5%-level.

In a third step, we conduct a **causal sensitivity analysis** to assess how potential violations of the exogeneity assumptions affect our findings during transition periods. To assess the sensitivity of our estimates to violations of the exogeneity assumption, we follow the procedure developed by Cinelli and Hazlett (2020), using the R-package *sensemakr*. This procedure does not allow us to include interactions and requires a linear specification. Hence, we subset our sample to transition periods, identified by our *transition (0-5)* dummy used in our original models 1 and 2, and replace our logistic specifications with linear probability models.

Relying on these models, we estimate changes in the statistical significance of *autonomy* during periods of regime transition, depending on the presence of hypothetical confounders that are associated to varying degrees with both *autonomy* and our outcome variables, *SDM onset* and *Violent SDM onset*. As a benchmark confounder, we employ *Previous SDMs*, which is associated with a higher probability that a group attains autonomy, a higher probability of SDM onset, and a lower probability of violent SDM onset.

Figure A3 shows the results of this procedure. Its horizontal axis captures the residual variation in the treatment (*autonomy*) explained by the hypothetical confounder; its vertical axis captures the residual variation of the respective outcome explained by it. The contours show the adjusted t-value that would be obtained in the presence of an unobserved confounder, depending on the hypothesized values of the sensitivity parameters. The red dots highlight scenarios in which there is a confounder that is 1x, 2x, and 3x as strongly correlated with the treatment and outcome variables, as compared to the benchmark confounder, *Previous SDMs*.

Two conclusions can be drawn from the results: First, as regards *SDM onset* (panel a), the coefficient of *autonomy* remains significant at the 5%-level, even in the presence of an additional confounder that is 3x as strongly correlated with it and with *SDM onset* as the benchmark confounder. Second, the same applies to *Violent SDM onset*, though the significance level remains less comfortably below the indicated threshold. Overall, this indicates that our results are not overly sensitive to violations of the exogeneity assumption. Specifically, an omitted confounder that is more than 3x as strongly correlated with autonomy and our dependent variables than *Previous SDMs* would be required to render our results non-significant at conventional statistical levels.

Appendix B.2.1: Exclusion of groups with SDMs in 3-year time period preceding last transition

Table A9. Results: Excluding groups with SDM in 3-year time period preceding last transition.

	**SDM onset**	**Violent SDM onset**	**SDM onset**	**Violent SDM onset**
	**Model 1 (all)**	**Model 2 (all)**	**Model 3 (transition only)**	**Model 4 (transition only)**
Autonomy	-0.092	-0.389	0.730**	0.908***
	(0.431)	(0.367)	(0.362)	(0.332)
Transition (0-5)	0.666***	-0.075		
	(0.228)	(0.227)		
Autonomy x transition (0-5)	1.125**	1.124**		
	(0.507)	(0.436)		
Constant	-3.331***	-1.176	-3.737**	-21.269***
	(1.148)	(1.198)	(1.633)	(7.468)
Wald $\chi 2$				
$\beta_{\text{Autonomy x transition (0-5)}} = 0$	4.9196*	6.6344*		
$\beta_{\text{Autonomy}} + \beta_{\text{Autonomy x transition (0-5)}} = 0$	8.2501**	7.8889**		
N	24295	30664	8245	11011
Log Likelihood	-1168.374	-832.274	-590.835	-349.871
AIC	2524.748	1854.548	1365.670	885.741

***p < .01; **p < .05; *p < .1; Country-clustered errors in parentheses. Cubic term for time dependence of dependent variable, control variables, and year- and region-fixed effects included but not reported.

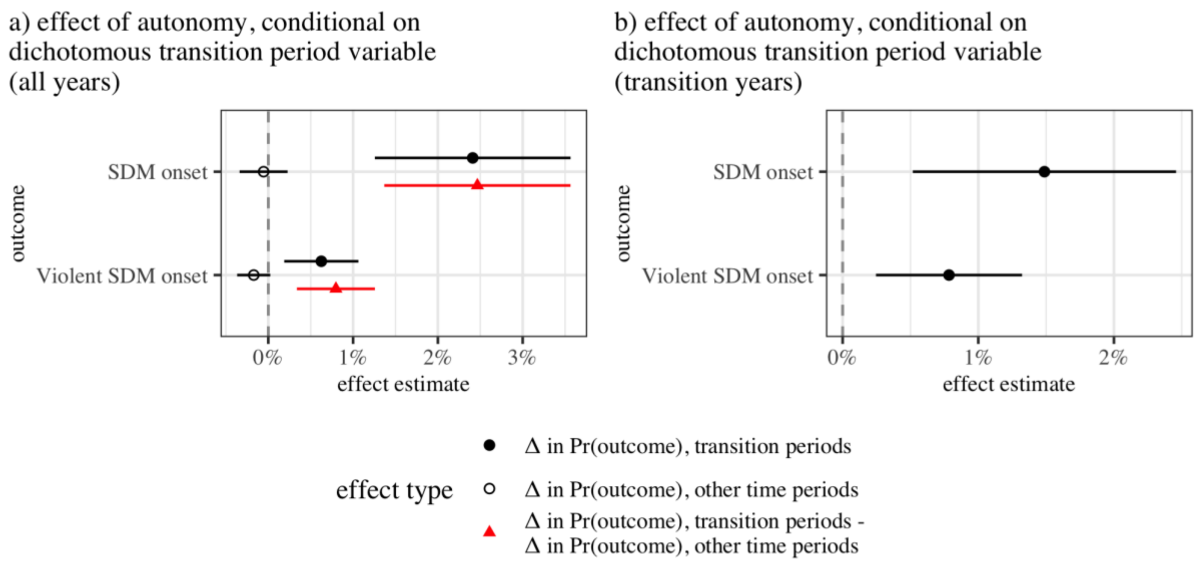

Figure A11. First and second-differences in the predicted probability of (violent) SDM onset, depending on autonomy and transition periods. Based on models 1-4 in table A9.

Appendix B.2.2: Incorporating group-fixed effects

Table A10. Results: incorporating group-fixed effects.

	SDM onset Model 1	Violent SDM onset Model 2	SDM onset Model 3	Violent SDM onset Model 4
Autonomy	-0.014	-0.001	-0.018	-0.001
	(0.021)	(0.006)	(0.021)	(0.006)
Transition (0-5)	0.009***	0.002		
	(0.003)	(0.002)		
Autonomy x transition (0-5)	0.064	0.007**		
	(0.043)	(0.003)		
Transition proximity			0.012***	0.003
			(0.003)	(0.002)
Autonomy x transition proximity			0.076*	0.007**
			(0.046)	(0.003)
Constant	0.047***	0.028*	0.044***	0.028*
	(0.017)	(0.015)	(0.017)	(0.015)
Wald $\chi 2$				
$\beta_{\text{Autonomy x transition (0-5)}} = 0$	2.2754	5.2917*		
$\beta_{\text{Autonomy}} + \beta_{\text{Autonomy x transition (0-5)}} = 0$	3.4657	1.2103		
$\beta_{\text{Autonomy x transition proximity}} = 0$			2.758	5.0355*
$\beta_{\text{Autonomy}} + \beta_{\text{Autonomy x transition proximity}} = 0$			3.5896	1.2445
N	24466	31044	24466	31044
R-squared	0.249	0.092	0.251	0.092
Adj. R-squared	0.227	0.070	0.229	0.070
Residual Std. Error	0.092 (df = 23769)	0.075 (df = 30327)	0.091 (df = 23769)	0.075 (df = 30327)
F Statistic	11.305*** (df = 696; 23769)	4.276*** (df = 716; 30327)	11.460*** (df = 696; 23769)	4.283*** (df = 716; 30327)

***p < .01; **p < .05; *p < .1; Country-clustered errors in parentheses. Cubic term for time dependence of dependent variable, control variables, and group- and year-fixed effects included but not reported.

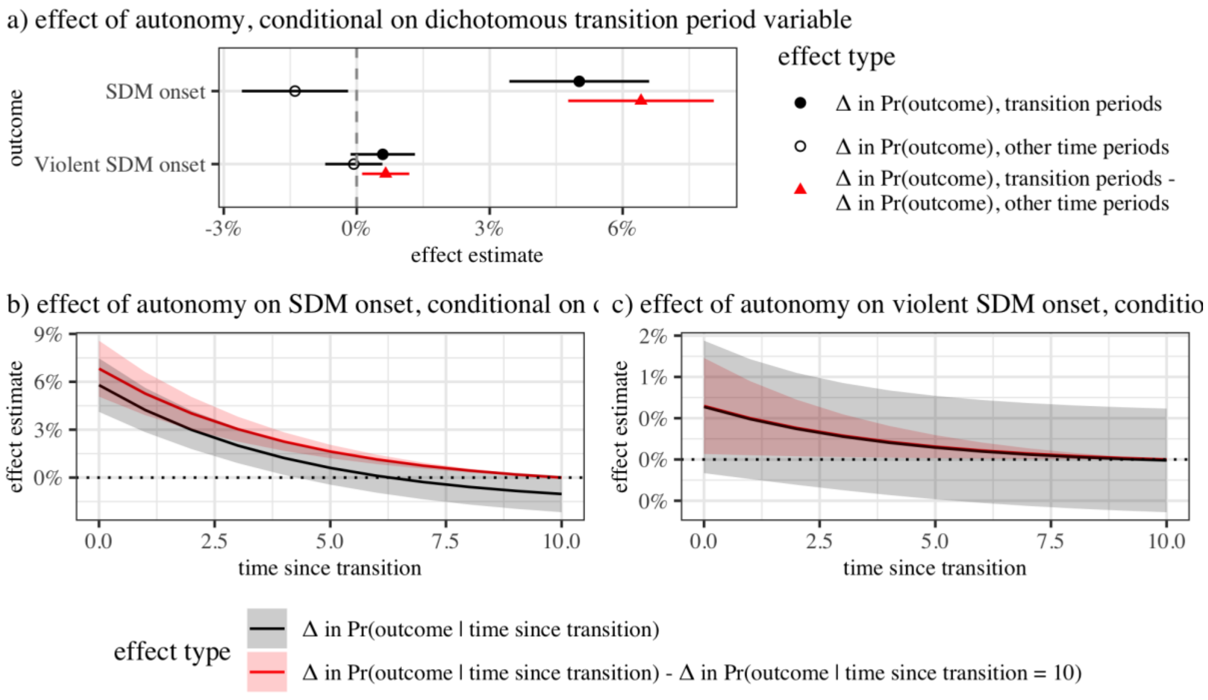

Figure A12. First and second-differences in the predicted probability of (violent) SDM onset, depending on autonomy and transition periods. Based on models 1-4 in table A10.

Appendix B.2.3: Causal sensitivity analyses

a) SDM onset b) Violent SDM onset

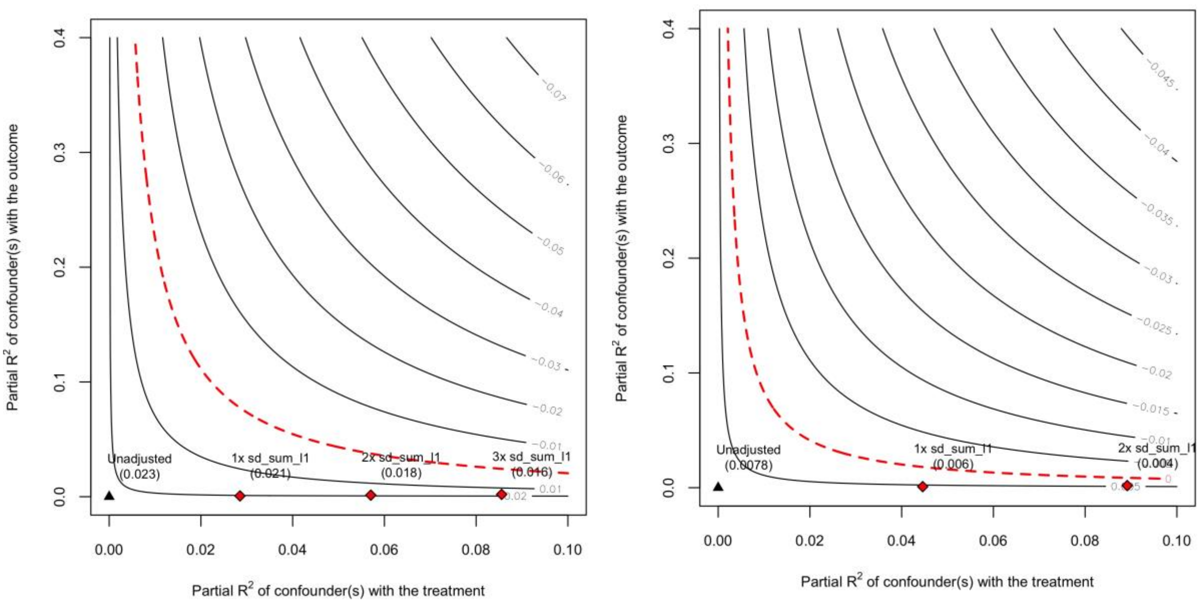

Figure A13. Sensitivity analyses. Benchmark confounder = *Previous SDMs*.

Appendix B.3: Unpacking transitions

In this section, we scrutinize the effect of regime transitions more carefully, distinguishing between multiple types of transitions, and analyzing different time frames.[5]

In a first step, we disaggregate our original, binary transition variable into its underlying components and thereby distinguish between **different types of transitions** (appendix B.3.1). We distinguish between *state transitions*, which refer to periods following the creation of new states, *democratic transitions*, and *autocratic transitions*, thereby adopting a more fine-grained classification.

Our results remain robust to these alterations, but provide more nuance on the relationship between autonomy and (violent) SDM onsets, conditional on the type of transition. First, we find that the **positive association of autonomy with initial SDM onsets during regime transitions is driven by periods following democratization processes**. State transitions (creation of new states) are associated with new self-determination movements, irrespective of the previous level of autonomy. Second, we find that the **positive association of autonomy with violent SDM onsets is greatest following state transitions (creation of new states),** though the coefficient and marginal effect is also significant for autocratization processes.

Following our hypothesized mechanisms, this may be because the degree of uncertainty and the government's willingness to accommodate challengers differs between different types of transitions. New democracies may be more accommodative to challengers, enabling autonomous groups to press their demands in a peaceful way (Cohen 1997; Hechter 2000). Moreover, new democracies are likely more transparent in building new institutions and thereby in a better position to settle self-determination disputes peacefully (cf. Hale 2008, p. 81). This may enable new democracies to react to SDMs with negotiated concessions that avoid violent confrontations. In contrast, the consolidation of autocracies is a process that yields reliable information on the new regime's intentions more quickly (Cederman et al., 2010), such as its willingness to accommodate minorities or violently repress their demands. Moreover, autocratizing and newly created states might be particularly reluctant to react to self-determination demands with concessions, as they may fear emboldening other challengers that might question the new autocratic government's monopoly to power or new states' still unconsolidated international borders (Walter 2006). As a result, autonomous groups might be more likely to violently escalate self-determination movements during autocratizing and state transitions, but not during democratizing transitions.

In a second step, we alter our **operationalization of regime transitions** (appendix B.3.2). First, we employ an alternative measure for regime transitions, based on the corrected **Polity index**. Here, we code *regime transition periods* for each year during which our revised regime durability indicator, constructed analogously to the original Polity regime durability indicator while excluding PARREG, is 5 or lower (that is, within 5 years of a transition). Analogously to the original Polity regime durability indicator, this captures each year within 5 years of changes that saw either:

1. at least a three-point change in the revised Polity score, which excludes PARREG, over a three-year period; or
2. the end of a transition period defined by the lack of stable political institutions, denoted by a standardized Polity authority score: -66 (foreign interruption), -77 (interregnum), -88 (transition).

Second, we use **alternative time windows** (3 years after transition / only transition years) to code our binary transition measure, based on V-Dem, and employ **alternative half-year values** (5 and 1 years) to code our regime transition proximity measure. Finally, we modify our original models by incorporating the squared term of our Polity measure to make sure our effects are not driven by **anocratic regime types** more broadly, that might be associated with many regime transitions in our sample. Reassuringly, our results remain remarkably unsensitive to these incisive changes of our transition variables and the additional control for anocracy. This indicates that our findings are not driven by our specific operationalization of transition periods nor by anocratic regime types.

[5] Where not otherwise specified, we employ the same group- and country-year control variables as in our main specification. However, we omit their coefficient from our reports, which enables us to provide an efficient overview of our main results. Our R script in the supplementary material creates the full tables.

Appendix B.3.1: Different types of regime transitions

Table A11. Results: different types of transitions.

	SDM onset	Violent SDM onset	SDM onset	Violent SDM onset
	Model 1	Model 2	Model 3	Model 4
Autonomy	-0.032	-0.283	-0.093	-0.339
	(0.449)	(0.318)	(0.441)	(0.338)
Democratic transition (0-5)	0.121	-0.120		
	(0.230)	(0.266)		
Autocratic transition (0-5)	-0.125	-0.420		
	(0.348)	(0.445)		
State transition (0-5)	1.240**	0.284		
	(0.487)	(0.447)		
Autonomy x democratic transition (0-5)	1.670***	0.582		
	(0.568)	(0.407)		
Autonomy x autocratic transition (0-5)	-0.893	1.301**		
	(0.936)	(0.584)		
Autonomy x state transition (0-5)	-1.149	0.972		
	(0.870)	(0.729)		
Democratic transition proximity			0.306	-0.118
			(0.263)	(0.341)
Autocratic transition proximity			-0.278	-0.993
			(0.432)	(0.650)
State transition proximity			2.417***	1.044*
			(0.618)	(0.622)
Autonomy x democratic transition proximity			1.634***	0.386
			(0.631)	(0.523)
Autonomy x autocratic transition proximity			-0.811	1.885**
			(1.364)	(0.790)
Autonomy x state transition proximity			-1.093	1.716**
			(1.311)	(0.812)
Constant	-2.811***	-1.112	-3.150***	-1.330
	(1.084)	(1.144)	(1.160)	(1.157)
Wald $\chi 2$				
$\beta_{\text{Autonomy x democratic transition (0-5)}} = 0$	8.6299**	2.0432		
$\beta_{\text{Autonomy}} + \beta_{\text{Autonomy x democratic transition (0-5)}} = 0$	17.116***	0.8294		
$\beta_{\text{Autonomy x autocratic transition (0-5)}} = 0$	0.9107	4.9707*		
$\beta_{\text{Autonomy}} + \beta_{\text{Autonomy x autocratic transition (0-5)}} = 0$	0.7516	3.5611		
$\beta_{\text{Autonomy x state transition (0-5)}} = 0$	1.7429	1.7803		
$\beta_{\text{Autonomy}} + \beta_{\text{Autonomy x state transition (0-5)}} = 0$	1.2813	0.7471		
$\beta_{\text{Autonomy x democratic transition proximity}} = 0$			6.7088**	0.5466
$\beta_{\text{Autonomy}} + \beta_{\text{Autonomy x democratic transition proximity}} = 0$			12.552***	0.0135
$\beta_{\text{Autonomy x autocratic transition proximity}} = 0$			0.3537	5.6971*
$\beta_{\text{Autonomy}} + \beta_{\text{Autonomy x autocratic transition proximity}} = 0$			0.3905	4.4187*
$\beta_{\text{Autonomy x state transition proximity}} = 0$			0.6951	4.4717*
$\beta_{\text{Autonomy}} + \beta_{\text{Autonomy x state transition proximity}} = 0$			0.6815	2.444
N	24466	31044	24466	31044
Log Likelihood	-1167.181	-864.697	-1150.570	-856.480
AIC	2530.361	1927.393	2497.141	1910.960

***p < .01; **p < .05; *p < .1; Country-clustered errors in parentheses. Cubic term for time dependence of dependent variable, control variables, and year- and region-fixed effects included but not reported.

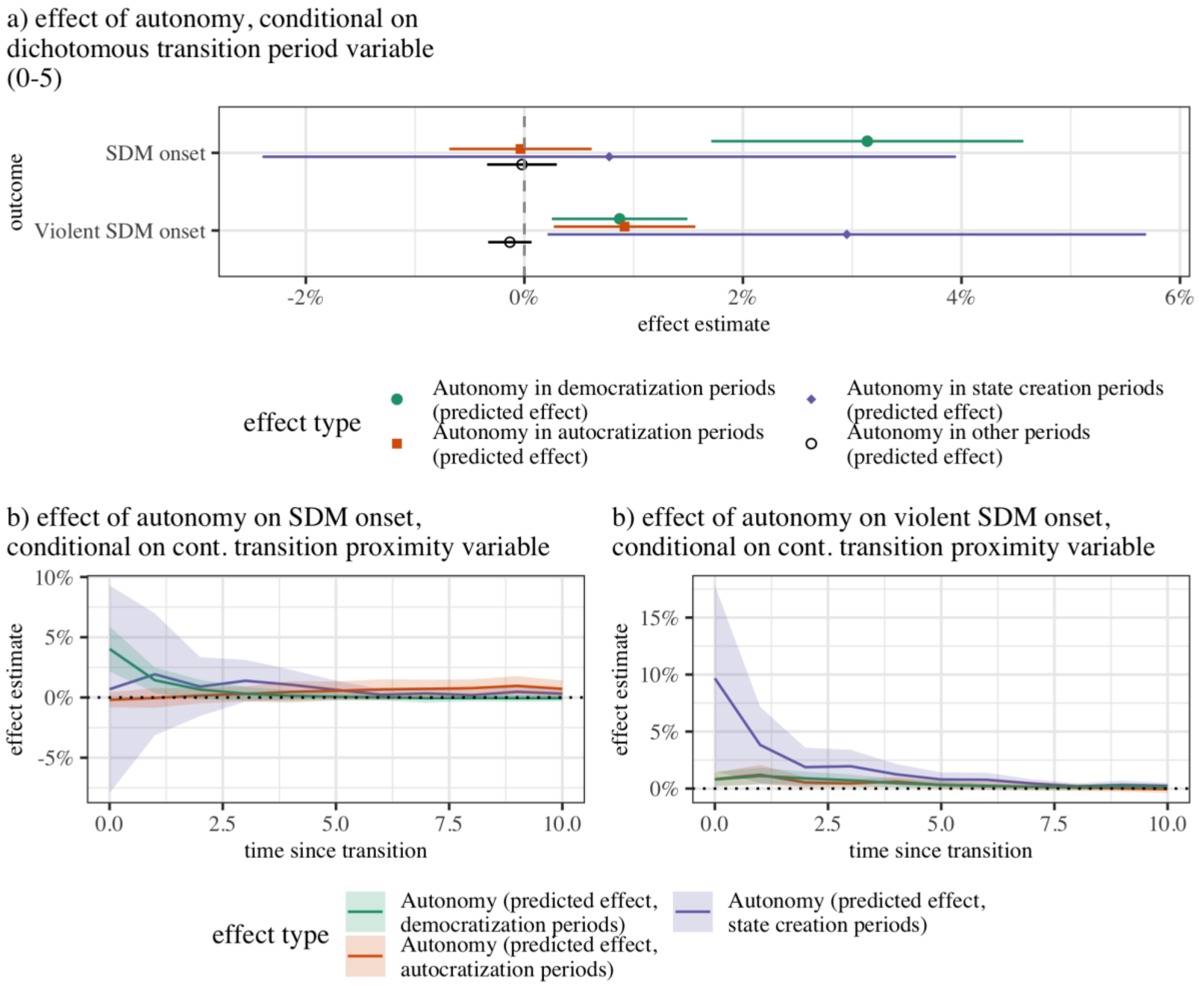

Figure A14. First and second-differences in the predicted probability of (violent) SDM onset, depending on autonomy and (democratizing, autocratizing, and state creation-based) transition periods. Based on models 1-4 in table A11.

Appendix B.3.2: Different operationalization of regime transitions and control for anocracy

Table A12. Results: different measure for transition (based on corrected Polity durability measure).

	**SDM onset Model 1**	**Violent SDM onset Model 2**	**SDM onset Model 3**	**Violent SDM onset Model 4**
Autonomy	-0.191	-0.119	-0.172	-0.082
	(0.384)	(0.261)	(0.359)	(0.286)
Transition (0-5)	0.836***	0.186		
	(0.234)	(0.243)		
Autonomy x transition (0-5)	1.514***	0.988**		
	(0.416)	(0.393)		
Transition proximity			1.368***	0.755**
			(0.277)	(0.314)
Autonomy x transition proximity			1.648***	1.013**
			(0.406)	(0.495)
Constant	-4.168***	-1.607	-4.778***	-2.209*
	(1.092)	(1.146)	(1.083)	(1.190)
Wald $\chi 2$				
$\beta_{\text{Autonomy x transition (0-5)}} = 0$	13.228***	6.3299*		
$\beta_{\text{Autonomy}} + \beta_{\text{Autonomy x transition (0-5)}} = 0$	18.475***	7.0196**		
$\beta_{\text{Autonomy x transition proximity}} = 0$			16.49***	4.1927*
$\beta_{\text{Autonomy}} + \beta_{\text{Autonomy x transition proximity}} = 0$			24.071***	6.2355*
N	24466	31044	24466	31044
Log Likelihood	-1155.364	-865.895	-1140.358	-859.266
AIC	2498.728	1921.789	2468.715	1908.532

***p < .01; **p < .05; *p < .1; Country-clustered errors in parentheses. Cubic term for time dependence of dependent variable, control variables, and year- and region-fixed effects included but not reported.

Table A13. Results: Different transition time windows (3/0 years).

	**SDM onset Model 1**	**Violent SDM onset Model 2**	**SDM onset Model 3**	**Violent SDM onset Model 4**
Autonomy	-0.124	-0.300	-0.074	-0.213
	(0.428)	(0.352)	(0.410)	(0.344)
Transition (0-3)	0.720***	0.013		
	(0.225)	(0.231)		
Autonomy x transition (0-3)	1.179**	1.168***		
	(0.514)	(0.421)		
Transition (0)			0.810***	0.171
			(0.208)	(0.232)
Autonomy x transition (0)			1.108**	1.089**
			(0.527)	(0.446)
Constant	-3.067***	-1.104	-2.989***	-1.079
	(1.101)	(1.165)	(1.075)	(1.191)
Wald $\chi 2$				
$\beta_{\text{Autonomy x transition (0-3)}} = 0$	5.2616*	7.6862**		
$\beta_{\text{Autonomy}} + \beta_{\text{Autonomy x transition (0-3)}} = 0$	7.9618**	11.249***		
$\beta_{\text{Autonomy x transition}} = 0$			4.4268**	5.9561*
$\beta_{\text{Autonomy}} + \beta_{\text{Autonomy x transition}} = 0$			7.0997**	8.825**
N	24466	31044	24466	31044
Log Likelihood	-1168.797	-865.237	-1165.314	-864.211
AIC	2525.593	1920.475	2518.628	1918.422

***p < .01; **p < .05; *p < .1; Country-clustered errors in parentheses. Cubic term for time dependence of dependent variable, control variables, and year- and region-fixed effects included but not reported.

Table A14. Results: different decaying times for transition proximity measure (5/1 year half-life).

	SDM onset<br>Model 1	Violent SDM onset<br>Model 2	SDM onset<br>Model 3	Violent SDM onset<br>Model 4
Autonomy	-0.089	-0.443	-0.094	-0.296
	(0.463)	(0.432)	(0.427)	(0.355)
Transition proximity (d5)	0.955***	-0.013		
	(0.281)	(0.268)		
Autonomy x transition proximity (d5)	1.094*	1.267**		
	(0.572)	(0.533)		
Transition proximity (d1)			1.134**	1.230***
			(0.532)	(0.450)
Autonomy x transition proximity (d1)			0.854***	0.106
			(0.229)	(0.246)
Constant	-3.279***	-1.192	-3.050***	-1.077
	(1.136)	(1.155)	(1.085)	(1.190)
Wald $\chi 2$				
$\beta_{\text{Autonomy x transition proximity (d5)}} = 0$	3.6587	5.6596*		
$\beta_{\text{Autonomy}} + \beta_{\text{Autonomy x transition proximity (d5)}} = 0$	7.0216**	8.976**		
$\beta_{\text{Autonomy x transition proximity (d1)}} = 0$			4.5384*	7.4836**
$\beta_{\text{Autonomy}} + \beta_{\text{Autonomy x transition proximity (d1)}} = 0$			7.4946**	10.896***
N	24466	31044	24466	31044
Log Likelihood	-1168.114	-866.629	-1165.428	-863.877
AIC	2524.228	1923.259	2518.857	1917.753

***p < .01; **p < .05; *p < .1; Country-clustered errors in parentheses. Cubic term for time dependence of dependent variable, control variables, and year- and region-fixed effects included but not reported.

Table A15. Results: control for anocracy (squared polity score).

	SDM onset<br>Model 1	Violent SDM onset<br>Model 2	SDM onset<br>Model 3	Violent SDM onset<br>Model 4
Autonomy	-0.095	-0.325	-0.094	-0.396
	(0.435)	(0.364)	(0.450)	(0.397)
Transition (0-5)	0.677***	-0.098		
	(0.229)	(0.218)		
Autonomy x transition (0-5)	1.111**	1.105***		
	(0.508)	(0.420)		
Transition proximity			0.934***	-0.019
			(0.265)	(0.264)
Autonomy x transition proximity			1.128**	1.289***
			(0.545)	(0.498)
Normalized polity score	0.534	1.361	-0.061	1.133
	(1.409)	(1.454)	(1.457)	(1.471)
Normalized polity score 2	0.053	-1.315	0.560	-1.146
	(1.359)	(1.252)	(1.403)	(1.252)
Constant	-3.069***	-1.368	-3.052***	-1.336
	(1.100)	(1.148)	(1.103)	(1.186)
Wald $\chi 2$				
$\beta_{\text{Autonomy x transition (0-5)}} = 0$	4.7812*	6.9221**		
$\beta_{\text{Autonomy}} + \beta_{\text{Autonomy x transition (0-5)}} = 0$	7.8437**	9.4125**		
$\beta_{\text{Autonomy x transition proximity}} = 0$			4.2889*	6.6917**
$\beta_{\text{Autonomy}} + \beta_{\text{Autonomy x transition proximity}} = 0$			7.6129**	10.299**
N	24466	31044	24466	31044
Log Likelihood	-1172.669	-866.149	-1166.746	-864.837
AIC	2535.338	1924.298	2523.492	1921.674

***p < .01; **p < .05; *p < .1; Country-clustered errors in parentheses. Cubic term for time dependence of dependent variable, control variables, and year- and region-fixed effects included but not reported.

Appendix B.4: Alterations to the dependent variable, sample, and specification

In this section, we probe the robustness of our results to general alterations of the dependent variable, sample, and specification. In a first step, we employ a broader **dependent variable**, which captures all *SDM radicalization* events, rather than only initial SDM onsets (appendix B.4.1). This accounts for the possibility that, during regime transitions, autonomous groups might switch from autonomy-seeking to more radical demands, such as full independence or irredentism. Our dependent variable in these analyses, *SDM radicalization*, captures group-wise switches from (1) no demands to autonomy-seeking demands, (2) from autonomy-seeking to secessionist demands, and (3) from secessionist to irredentist demands (Cederman et al. 2022). As their demands cannot radicalize further by definition, we exclude groups with irredentist demands in the previous year from our sample. Our results remain comparable in these analyses, indicating that uncertainty during regime transitions may not only prompt autonomous groups to initiate and violently escalate SDMs, but also to radicalize their demands towards full independence or aggregation with a neighboring kin state.

In a second step, we probe the robustness of our findings to **alterations to the sample** (appendix B.4.2). In one check, we **exclude the 1989 transition** in Central and Eastern Europe and the Former Soviet Union from our sample to make sure that our findings are not solely driven by this prominently-discussed set of transitions, which we revisit in our second set of analyses. In another check, we **exclude new states** in their first five years of existence from our sample altogether to make sure our findings are not solely driven by irredentism following the disintegration of multiethnic states that might not be fully captured by our control variables (Cederman et al., 2022). In a final check, we **exclude all groups that hold the highest political status position** according to EPR (Vogt et al. 2015) from our sample.[6]

In a third and final step, we probe the robustness of our findings to the particular **specification** we used (appendix B.4.3). A key concern is that the region- and year-fixed effects in our logistic regression models might entail a data loss, as groups without variation in the dependent variable are dropped in such specifications (Timoneda 2021). However, in our case, this concern should be attenuated, as our fixed effects capture comparably highly-aggregated groups. Indeed, there are recorded (violent) SDM onsets for almost every region and year, meaning that virtually no data is dropped in our main models. Nevertheless, we probe the sensitivity of our findings to this concern, by estimating a **linear probability model.** Reassuringly, our findings are almost unchanged.

Appendix B.4.1: Broader dependent variable (SDM radicalization)

Table A16. Results: broader dependent variable (SDM radicalization).

	**Model 1**	**Model 2**
Autonomy	-0.023	0.004
	(0.339)	(0.353)
Transition (0-5)	0.601^{**}	
	(0.235)	
Autonomy x transition (0-5)	0.838^{*}	
	(0.466)	
Transition proximity		0.924^{***}
		(0.234)
Autonomy x transition proximity		0.791
		(0.504)
Constant	-2.822^{***}	-3.006^{***}
	(0.944)	(0.956)
Wald $\chi 2$		
$\beta_{\text{Autonomy x transition (0-5)}} = 0$	3.229	
$\beta_{\text{Autonomy}} + \beta_{\text{Autonomy x transition (0-5)}} = 0$	6.4006*	
$\beta_{\text{Autonomy x transition proximity}} = 0$		2.4654
$\beta_{\text{Autonomy}} + \beta_{\text{Autonomy x transition proximity}} = 0$		5.5712*
N	32934	32934
Log Likelihood	-1482.646	-1473.540
AIC	3155.292	3137.080

$^{***}p < .01$; $^{**}p < .05$; $^{*}p < .1$; Country-clustered errors in parentheses. Cubic term for time dependence of dependent variable, control variables, and year- and region-fixed effects included but not reported.

[6] Again, if multiple groups jointly hold the highest status (e.g., Yugoslavia, where numerous groups are coded as 'senior partners') we identify the demographically largest of these groups as the most powerful, analogously to Bormann et al. (2017) (in the case of Yugoslavia, the Serbs).

Appendix B.4.2: Sample alterations

Table A17. Results: excluding CEE/FSU post-1989 transition.

	SDM onset Model 1	Violent SDM onset Model 2	SDM onset Model 3	Violent SDM onset Model 4
Autonomy	-0.341	-0.395	-0.325	-0.465
	(0.491)	(0.424)	(0.498)	(0.463)
Transition (0-5)	0.700***	0.048		
	(0.245)	(0.222)		
Autonomy x transition (0-5)	1.201**	0.872*		
	(0.497)	(0.469)		
Transition proximity			0.976***	0.150
			(0.282)	(0.265)
Autonomy x transition proximity			1.168**	1.030*
			(0.536)	(0.562)
Constant	-2.454*	-0.958	-2.559**	-0.976
	(1.257)	(1.528)	(1.281)	(1.566)
Wald $\chi 2$				
$\beta_{\text{Autonomy x transition (0-5)}} = 0$	5.8348*	3.448		
$\beta_{\text{Autonomy}} + \beta_{\text{Autonomy x transition (0-5)}} = 0$	4.7846*	3.6772		
$\beta_{\text{Autonomy x transition proximity}} = 0$			4.7375*	3.3526
$\beta_{\text{Autonomy}} + \beta_{\text{Autonomy x transition proximity}} = 0$			4.1941*	4.3262*
N	23187	28560	23187	28560
Log Likelihood	-1025.694	-760.267	-1021.062	-759.249
AIC	2239.388	1710.535	2230.123	1708.498

***p < .01; **p < .05; *p < .1; Country-clustered errors in parentheses. Cubic term for time dependence of dependent variable, control variables, and year- and region-fixed effects included but not reported.

Table A18. Results: excluding new states.

	SDM onset Model 1	Violent SDM onset Model 2	SDM onset Model 3	Violent SDM onset Model 4
Autonomy	-0.026	-0.320	-0.056	-0.407
	(0.436)	(0.347)	(0.449)	(0.371)
Transition (0-5)	0.454*	-0.087		
	(0.240)	(0.251)		
Autonomy x transition (0-5)	1.428***	0.966**		
	(0.496)	(0.421)		
Transition proximity			0.595**	-0.119
			(0.275)	(0.319)
Autonomy x transition proximity			1.523***	1.222**
			(0.511)	(0.499)
Constant	-3.480***	-1.791	-3.460***	-1.735
	(1.174)	(1.207)	(1.195)	(1.224)
Wald $\chi 2$				
$\beta_{\text{Autonomy x transition (0-5)}} = 0$	8.293**	5.2744*		
$\beta_{\text{Autonomy}} + \beta_{\text{Autonomy x transition (0-5)}} = 0$	21.777***	5.1085*		
$\beta_{\text{Autonomy x transition proximity}} = 0$			8.8735**	5.9816*
$\beta_{\text{Autonomy}} + \beta_{\text{Autonomy x transition proximity}} = 0$			24.77***	6.4591*
N	23180	29562	23180	29562
Log Likelihood	-951.921	-744.086	-948.684	-743.029
AIC	2089.842	1678.172	2083.367	1676.058

***p < .01; **p < .05; *p < .1; Country-clustered errors in parentheses. Cubic term for time dependence of dependent variable, control variables, and year- and region-fixed effects included but not reported.

Table A19. Results: excluding most powerful groups.

	SDM onset<br>Model 1	Violent SDM onset<br>Model 2	SDM onset<br>Model 3	Violent SDM onset<br>Model 4
Autonomy	-0.103	-0.372	-0.101	-0.446
	(0.438)	(0.356)	(0.452)	(0.388)
Transition (0-5)	0.656^{***}	-0.053		
	(0.228)	(0.221)		
Autonomy x transition (0-5)	1.180^{**}	1.117^{***}		
	(0.519)	(0.420)		
Transition proximity			0.899^{***}	0.021
			(0.259)	(0.266)
Autonomy x transition proximity			1.175^{**}	1.308^{***}
			(0.561)	(0.496)
Constant	-3.025^{***}	-1.238	-3.133^{***}	-1.233
	(1.153)	(1.184)	(1.166)	(1.223)
Wald $\chi2$				
$\beta_{\text{Autonomy x transition (0-5)}} = 0$	5.1768*	7.0808**		
$\beta_{\text{Autonomy}} + \beta_{\text{Autonomy x transition (0-5)}} = 0$	8.5045**	8.2546**		
$\beta_{\text{Autonomy x transition proximity}} = 0$			4.3869*	6.9626**
$\beta_{\text{Autonomy}} + \beta_{\text{Autonomy x transition proximity}} = 0$			7.7209**	9.2474**
N	21661	28066	21661	28066
Log Likelihood	-1112.737	-846.906	-1107.134	-845.459
AIC	2411.474	1881.811	2400.269	1878.917

$^{***}p < .01$; $^{**}p < .05$; $^{*}p < .1$; Country-clustered errors in parentheses. Cubic term for time dependence of dependent variable, control variables, and year- and region-fixed effects included but not reported.

Appendix B.4.3: Alternative specification

Table A20. Results: linear probability model.

	SDM onset<br>Model 1	Violent SDM onset<br>Model 2	SDM onset<br>Model 3	Violent SDM onset<br>Model 4
Autonomy	-0.004	-0.001	-0.004	-0.001
	(0.005)	(0.002)	(0.005)	(0.002)
Transition (0-5)	0.005^{**}	-0.001		
	(0.002)	(0.001)		
Autonomy x transition (0-5)	0.031^{*}	0.008^{**}		
	(0.018)	(0.003)		
Transition proximity			0.008^{***}	-0.001
			(0.003)	(0.002)
Autonomy x transition proximity			0.036^{*}	0.009^{**}
			(0.022)	(0.004)
Constant	0.072^{***}	0.058^{***}	0.069^{***}	0.058^{***}
	(0.022)	(0.013)	(0.022)	(0.013)
Wald $\chi2$				
$\beta_{\text{Autonomy x transition (0-5)}} = 0$	2.89	6.0646*		
$\beta_{\text{Autonomy}} + \beta_{\text{Autonomy x transition (0-5)}} = 0$	2.7101	4.3587*		
$\beta_{\text{Autonomy x transition proximity}} = 0$			2.7166	6.2392*
$\beta_{\text{Autonomy}} + \beta_{\text{Autonomy x transition proximity}} = 0$			2.5655	4.569*
N	24466	31044	24466	31044
R-squared	0.038	0.022	0.039	0.023
Adj. R-squared	0.034	0.019	0.035	0.020
Residual Std. Error	0.102 (df = 24372)	0.077 (df = 30949)	0.102 (df = 24372)	0.077 (df = 30949)
F Statistic	10.291^{***} (df = 93; 24372)	7.567^{***} (df = 94; 30949)	10.610^{***} (df = 93; 24372)	7.588^{***} (df = 94; 30949)

$^{***}p < .01$; $^{**}p < .05$; $^{*}p < .1$; Country-clustered errors in parentheses. Cubic term for time dependence of dependent variable, control variables, and year- and region-fixed effects included but not reported.

Appendix C: Territorial autonomy data (CEE-FSU sample)

In this appendix, we present our newly-collected data for territorial autonomy in the Central and Eastern Europe-Former Soviet Union region. This comprises the following countries: Poland, Hungary, Czechoslovakia, Czech Republic, Slovakia, Albania, Yugoslavia, Serbia, Montenegro, North Macedonia, Croatia, Bosnia and Herzegovina, Kosovo, Slovenia, Bulgaria, Moldova, Romania, Russia/USSR, Estonia, Latvia, Lithuania, Ukraine, Belarus, Armenia, Georgia, Azerbaijan, Turkmenistan, Tajikistan, Kyrgyzstan, Uzbekistan, and Kazakhstan.

In what follows, we explain how we identify territorial autonomy arrangements for this sample (appendix C.1) and provide detail on how we measure each administrative unit's degree of territorial autonomy with our indicators (appendix C.2). Next, we explain how we geo-code each administrative unit's boundaries (appendix C.3) and how we use these to aggregate our territorial autonomy data onto the ethnic group-level (appendix C.4). In appendix C.5, we provide examples for our coding procedure by tracing the evolution of territorial autonomy in our data for the case of the Soviet Union and Russia (1936-2017). Finally, in appendix C.6, we provide a list of all unique autonomy tiers and their aggregate autonomy scores in our sample.

Appendix C.1: Identification of territorial autonomy arrangements

In a first step, we **identify** any instances of territorial autonomy for all cases in the CEE-FSU region, for the time period 1946-2017. For the Soviet Union, we cover an extended time period that goes back until 1936, as necessitated by our robustness checks (see appendix E). For this purpose, we read and assess all constitutional texts that were operative at any time point for this sample. We checked all **constitutional events coded by the Comparative Constitutions Project** (Elkins, Ginsburg & Melton 2014). We obtained original texts for each constitutional event, predominantly relying on Hein Online's World Constitutions Illustrated database[7] and the Constitute Database.[8] In these texts, we identify arrangements that the constitution calls 'confederal', ‘federal’, ‘decentralized’, or ‘autonomous’ to derive a list of ‘candidate’ autonomous units.

We then assess, based on the obtained **constitutions and any autonomy statutes** referenced by it, each ‘candidate’ unit's formally-institutionalized degree of autonomy. For this purpose, we also rely on secondary data, if information on autonomy statutes was not readily publicly available. Most importantly, for cases covered by them, we rely on the detailed case descriptions by Hooghe and colleagues (2016).

Using these sources, and in each case documenting our sources and noting down details in text form (see appendix C.5), we code seven indicators, each normalized to a range from 0 to 1 (see appendix C.2), which we aggregate in several steps into an overall measure for each unit's degree of autonomy. Similar to the Regional Authority Index (RAI) (Hooghe et al. 2016), these indicators are grouped along three components (see figure A15):

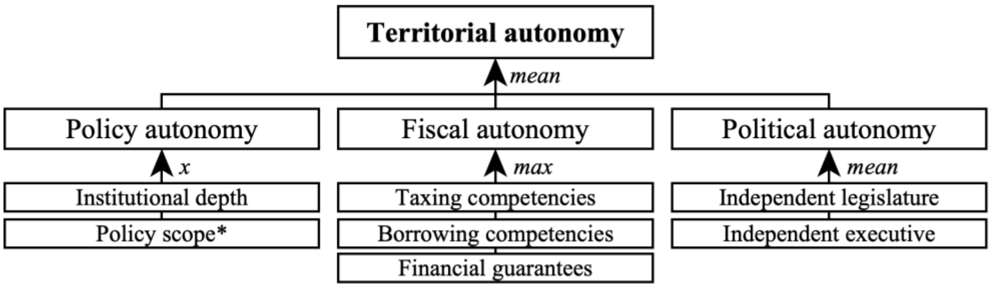

Figure A15. Territorial autonomy: components and indicators.
Note: All indicators and components are normalized to a range from 0 to 1. *Policy scope = weighted average of competencies over matters related to economy (weight = 1), welfare (1), culture (1), military (2), police (2), judiciary (2), institutional set-up (2), residual powers (3), community (3), secession rights (3).

- The first component, **policy autonomy**, captures each unit's competence to formulate its own policies. We capture this with two indicators: First, *institutional depth*, which refers to the degree to which a unit's decisions are independent from or circumscribed by central government interference. Second, *policy scope*, which captures the number and relative importance of issue areas that a unit may legislate on. As both aspects are jointly necessary for a unit to enjoy substantial policy autonomy, we multiply these indicators to arrive at an overall measure for *policy autonomy*.
- The second component, **fiscal autonomy**, encompasses financial measures that enable a unit to effectively take up its competencies. We capture this with three indicators: *taxing competencies* over the rate and base of minor and major taxes, *borrowing competencies*, depending on the need for central government authorization and restrictions, and *financial guarantees*, for instance through fiscal transfers from the center or natural resource rents. As these represent substitutable alternatives for endowing a unit

[7] Available at <https://home.heinonline.org/content/world-constitutions-illustrated/>.
[8] Available at <https://www.constituteproject.org/>.

with financial resources, we take their maximum to arrive at an overall measure for a unit's degree of *fiscal autonomy*.

- The third component, **political autonomy**, refers to a unit's ability to select its own governing institutions. We capture this with two indicators: *independent legislature* and *independent executive*. We conceive of these aspects as mutually reinforcing but not jointly necessary. We hence take their mean to arrive at an overall measure for *political autonomy*.

Each component (*policy autonomy*, *fiscal autonomy*, and *political autonomy*) is required for a tier to classify as fully autonomous. However, the absence of one component, while diminishing overall autonomy, does not make the others completely meaningless. In a final step, we hence take the average across all three components, each normalized to a range from 0 to 1, to obtain an overall measure for the degree of territorial autonomy enjoyed by each administrative unit, also ranging from 0 to 1.

Appendix C.2: Measurement of territorial autonomy indicators

Table A21. Measurement of territorial autonomy indicators.

Indicator	Values
1. Policy autonomy	
1.1 Institutional depth	{0,3}, 0 = no autonomous decision-making (e.g., no institutionalized autonomy or deconcentrated tier that implements central government decisions); 1 = decision-making subject to ex-ante central government approval; 2 = decision-making subject to ex-post central government veto; 3 = decision-making not subject to either central government approval or veto.
1.2 Policy scope	{0,20}, weighted sum of competencies coded as binary sub-indicators; includes competencies over matters related to economy (weight = 1), welfare (1), culture (1), military (2), police (2), judiciary (2), institutional set-up (2), residual powers (3), community (3), secession rights (3).
2. Fiscal autonomy	
2.1 Taxing competencies	{0,4}, 0 = none; 1 = competency to set the rate of minor taxes; 2 = competency to set base and rate of minor taxes; 3 = competency to set the rate of major taxes; 4 = competency to set base and rate of major taxes. *Note: major taxes include personal income, corporate, value added, and sales taxes.*
2.2 Borrowing competencies	{0,3}, 0 = none; 1 = borrowing possible under ex-ante authorization by the central government; 2 = borrowing possible without ex-ante authorization, but with restrictions; 3 = borrowing possible without authorization or restrictions.
2.3 Financial guarantees	{0,4}, sum of two sub-indicators: 1. explicit targeting of unit with guarantee (1 = vague, e.g. provisions that ‘all subnational units’ are entitled to the financial guarantee; 2 = explicit, e.g. provision that ‘specific unit X’ is entitled to the financial guarantee) and 2. type of financial guarantee (1 = minor, 2 = major). *Note: major financial guarantees include income from major taxes, as defined above, explicitly-specified percentages of natural resource rents accruing in the unit's territory, and explicitly-specified percentages of total national income redistributed to the unit.*
3. Political autonomy	
3.1 Independent legislature	{0,2}, 0 = subnational legislature does not exist or is fully appointed by the central government; 1 = subnational legislature is partly selected by the local population or local elites, partly appointed by the central government; 2 = subnational legislature is fully selected by the local population or local elites.
3.2 Independent executive	{0,2}, 0 = subnational executive does not exist or is fully appointed by the central government; 1 = subnational executive is partly selected by the local population or local elites, partly appointed by the central government; 2 = subnational executive is fully selected by the local population or local elites.

Note: All indicators are subsequently normalized to a range between 0 and 1 before aggregation. Details on tier-level territorial autonomy coding, including indicator-level values, comments, and references to relevant constitutional articles and autonomy statutes are included in the coding comments attached to the dataset.

Appendix C.3: Geo-coding of administrative boundaries

To aggregate our territorial autonomy scores onto the ethnic group-level, we require geo-coded polygons for each administrative unit's boundaries. To obtain these, we start off with the most recent first-order administrative unit polygons, which we take from the **Database of Global Administrative Areas (GADM)**.[9] In cases where we need to code subordinate units, we rely on information on second-order administrative unit polygons, again from

[9] Available at <http://gadm.org/data.html>.

GADM. In such cases, we use the polygons of subordinate units and the spatial difference from their respective first-order units (e.g., in Yugoslavia, Kosovo and the spatial difference between Serbia and Kosovo). We then consult additional sources to identify changes in these boundaries over time. For this purpose, we follow Law (2010) and the accompanying webpage: <http://www.statoids.com>. To obtain polygons for historical internal administrative boundaries, we conduct three complementary procedures:

1) We apply a first procedure in cases where historical administrative units were the aggregate of administrative units coded by GADM. This is the case if regions or states were gradually split up into their constituent areas over time. In such cases, we **backwards-aggregated their polygons**, following the description by Law (2010) and the accompanying webpage: <http://www.statoids.com>.
2) If historical boundaries cannot be backwards-aggregated (see procedure 1 above) and were in place in 1998, we rely on **ESRI's Country Administrative Units 1998 Dataset**,[10] which comprises polygons of all first-order administrative units that existed in that year.
3) Finally, if neither of these two procedures were sufficient to obtain historical boundaries, we looked up and manually **geo-referenced historical maps**. To obtain images of such maps, we relied on the University of Texas at Austin's Perry-Castañeda Library (PCL) Map Collection,[11] David Rumsey Map Collection,[12] and Old Maps Online.[13] We then georeferenced these images in the geographical information software QGIS. Using QGIS, we manually coded polygons corresponding to the historical boundaries visible on these maps.

Appendix C.4: Aggregation onto group-level

We aggregate the autonomy tier-level information on the group level by considering the degree of autonomy that the average group member lives under in a given year. To this end, we need to obtain estimates for each group's population in each administrative unit. For this purpose, we proceed as follows:

- **Spatial intersection:** First, we intersect the geocoded boundaries of all administrative units (see appendix C.3) with each group's settlement patterns, provided by the Geo-EPR dataset (Vogt et al. 2015). This yields the area of overlap. We excluded all intersections that do not overlap temporally (e.g., where the administrative unit was introduced after a group's settlement pattern has been replaced with a different one according to Geo-EPR).
- **Calculation of each intersection's area and population density:** Second, we calculate the area of each of these intersections. At the same time, we overlay them with a raster layer that contains information on the time-variant local population density, based on the History Database of the Global Environment (HYDE version 3.2, Klein Goldewijk et al. 2017), interpolated linearly for years without data. This allows us to adjust my estimates and account for large territorial units that nevertheless feature low populations (e.g. in Siberia).
- **Intersection of intersections and calculation of 'share factor'**: Third, we intersected each intersection with all other intersections that were operative in the same country year, according to the start and end dates of each administrative unit and ethnic settlement pattern. This enables us to calculate a 'share factor' that accounts for the degree to which each group's spatial overlap with any territorial unit is shared territory with other groups (and weigh accordingly in the final formula):

$$share\ factor_{g,u} = \frac{size_g \times\ area_{g,u} \times density_{g,u}}{\sum_{g=1}^{G} size_g \times\ area_{g,u} \times density_{g,u}} \ (1)$$

  where $size_g$ is the group's overall size a a share of the state's population (Vogt et al. 2015), $area_{g,u}$ is the area of the calculated intersection area in 1000's of square kilometers, and density is the average population per square kilometer in this area from GPW.
- **Calculation of 'absolute' group size in each territorial unit in each year:** The resulting information enables us to calculate the 'absolute' group size in each unit for a given year, as a fraction of the state's total population. To this end, we employ the following formula:

$$size_{g,u} = \frac{size_g \times area_{g,u} \times density_{g,u} \times share\ factor_{g,u}}{\sum_{u=1}^{U} size_g \times area_{g,u} \times density_{g,u} \times share\ factor_{g,u}} \ (2)$$

  This yields an estimate, ranging between 0 and 1 of a group's 'absolute' size (as a fraction of the state's total population) in a given unit and year. In a final step, we adjusted this estimate to make sure the sum of these estimates adds up to the group's total size as given by the EPR dataset to account for slight deviations and rounding errors.

Using this measure of absolute group size, we can calculate the average degree of autonomy a group member lives under in each given year by:

$$autonomy_g = \frac{size_{g,u} \times autonomy_u}{\sum_{u=1}^{U} size_{g,u}} \ (3)$$

[10] Available at <https://www.arcgis.com/sharing/rest/content/items/1ee881e919e1477a926f74586ddbdba9/info/metadata/metadata.xml?format=default&output=html>.
[11] Available at <https://maps.lib.utexas.edu/maps/index.html>.
[12] Available at <https://www.davidrumsey.com/>.
[13] Available at <https://www.oldmapsonline.org/>.

Appendix C.5: Coding examples

In this section, we illustrate how we coded our territorial autonomy indicators. We do so for the example of the Soviet Union and Russia, covering the time period between 1936 and 2017. We display our coding notes for five periods, which together cover the most major changes in institutionalized autonomy: 1936-1976 (the Soviet Union's second constitution), 1977-1989 (the Soviet Union's third constitution), Russia in the immediate post-transition period (1990 and 1991), and Russia between 2011-2017. In each case, we provide maps chronicling the evolution of our geo-coded administrative units (see appendix C.3) and their unit-wise autonomy scores (see appendices C.1-C.2) during the respective time periods.

USSR, 1936-1976

General information

[Federal or decentralized | Autonomous territories]: The Soviet Union is federal. It is divided into several Soviet Socialist Republics. There are ethnic autonomous areas at different levels: Autonomous Socialist Republics, Autonomous Provinces, and Autonomous Areas. *(Arts. 13; 22-29; 20)*

Autonomous Oblast and Okrug

- **Policy Autonomy: Institutional Depth [2]:** Each autonomous oblast and okrug is subordinate to the higher-level Union Republic, which determines their electoral modalities, whose law is superior and whose constitution determines their power. The executive organs of the autonomous republics are accountable to the superior level as well. **Policy Scope [2: Economy, Culture, Welfare, Judiciary]:** The autonomous oblast and okrug direct the government organs that are subordinate to them, ensure public order, the observance of the laws, protect citizen rights, direct local economic and cultural affairs and draw up and approve local budgets. *(Arts. 96; 97; 98; 101; 108)*
- **Financial Autonomy: Taxing competencies [0]:** Based on the fact that the fixing of taxes is a federal competence and that nowhere are any other fiscal powers specified explicitly, it appears that the autonomous oblast and okrug have no fiscal authority. Rather, they seem to depend on redistribution from the centre. **Borrowing competencies [0]:** Based on the fact that loans are a federal competence and that nowhere are any other borrowing powers specified explicitly, it appears that the autonomous oblast and okrug have no borrowing authority. Rather, they seem to depend on redistribution from the centre. **Fiscal guarantees [0].** *(Arts. 14; 57-61)*
- **Political Autonomy: Legislature [2]:** Each autonomous oblast and okrug has a Soviet of Working People's Deputies which is elected by its 'working people'. The precise modalities are determined in each case by the superior Union Republic. **Executive [1]:** Each Soviet of Working People's Deputies elects an executive committee, which consists of a Chairman, Vice-Chairman, a secretary and other members. The executive is also accountable to the next higher level, i.e., to the Union Republic. *(Arts. 94; 95; 96; 99; 100)*

Autonomous Republics

- **Policy Autonomy: Institutional Depth [2]:** Each autonomous republic has its own constitution which has to be in full conformity with the constitution of the Union Republic above it. The autonomous republics are supervised by their higher-level union republics., which confirms their constitutions and defines their territorial boundaries. The council of ministers of the higher level entity can further suspend their decisions directly. **Policy Scope [3: Economy, Culture, Welfare, Military, Police, Judiciary, Institutions]:** It appears that the Autonomous Republics have a similar policy scope as the Union Republics, but without the residual powers and rights to secession. *(Arts. 60; 82; 92; 107)*
- **Financial Autonomy: Taxing competencies [0]:** Based on the fact that the fixing of taxes is a federal competence and that nowhere are any other fiscal powers specified explicitly, it appears that the autonomous republics have no fiscal authority. Rather, they seem to depend on redistribution from the centre. **Borrowing competencies [0]:** Based on the fact that loans are a federal competence and that nowhere are any other borrowing powers specified explicitly, it appears that the autonomous republics have no borrowing authority. Rather, they seem to depend on redistribution from the centre. **Fiscal guarantees [0].** *(Arts. 14; 57-61)*
- **Political Autonomy: Legislature [2]:** Each Autonomous Republic directly elects a Supreme Soviet, which is its highest legislative organ. **Executive [2]:** Each Supreme Soviet of an autonomous republic elects its Presidium and appoints a council of ministers. *(Arts. 89; 90; 91; 93)*

Union Republics

- **Policy Autonomy: Institutional Depth [2]:** The federal government exercises oversight over the Union Republics to ensure the conformity of their constitution with the overall constitution. The federal government has to approve any border changes (although the approval of the affected the republics is required). In addition, in case of conflict, Union law prevails. **Policy Scope [4: Economy, Culture, Welfare, Military, Police, Judiciary, Institutions, Residual powers, Secession]:** The division of powers between the Soviet Union and the Union Republics is made via a list of competences given to the federal government and the provision that the republics have residual powers. The exclusive competences

of the federal state are international relations, war and diplomacy, foreign trade, the approval of economic plans, the state budget and distribution of revenues between the Union, Republican and local budgets, the administration of banks and industrial, agricultural and trading enterprises and the direction of industrial and building policy, important transport and communications, monetary policy and the credit system, state insurance, loans, basic principles of land tenure, the use of mineral wealth, forests, and water, the definition of basic principles of education and public health, economic statistics, fundamental labor legislation, fundamental judicial system and procedure, fundamental civil and criminal legislation, citizenship and immigration, and marriage and family law. Each union republic has its own constitution, the right to secede from the USSR, its own military formations, and rights to enter into direct relations with foreign states. Furthermore, explicitly reserved competences are also oversight over its lower-level autonomous republics and the definition of their boundaries, the approval of economic plans and the budget, rights of amnesty, and representation in international relations. *(Arts. 14; 15; 16; 17; 17a; 18; 18b; 20; 57-60; 106)*

- **Financial Autonomy: Taxing competencies [0]:** Based on the fact that the fixing of taxes is a federal competence and that nowhere are any other fiscal powers specified explicitly, it appears that the Union Republics have no fiscal authority. Rather, they seem to depend on redistribution from the centre. **Borrowing competencies [0]:** Based on the fact that loans are a federal competence and that nowhere are any other borrowing powers specified explicitly, it appears that the Union Republics have no borrowing authority. Rather, they seem to depend on redistribution from the centre. **Fiscal guarantees [0].** *(Arts. 14; 57-61)*
- **Political Autonomy: Legislature [2]:** Each Union Republic has a Supreme Soviet that is elected by the citizens directly. **Executive [2]:** The Supreme Soviet elects a Presidium consisting of the President of the Presidium, Vice-President, a Secretary, and further members. It also selects a council of ministers. *(Arts. 57; 58; 59; 61; 83)*

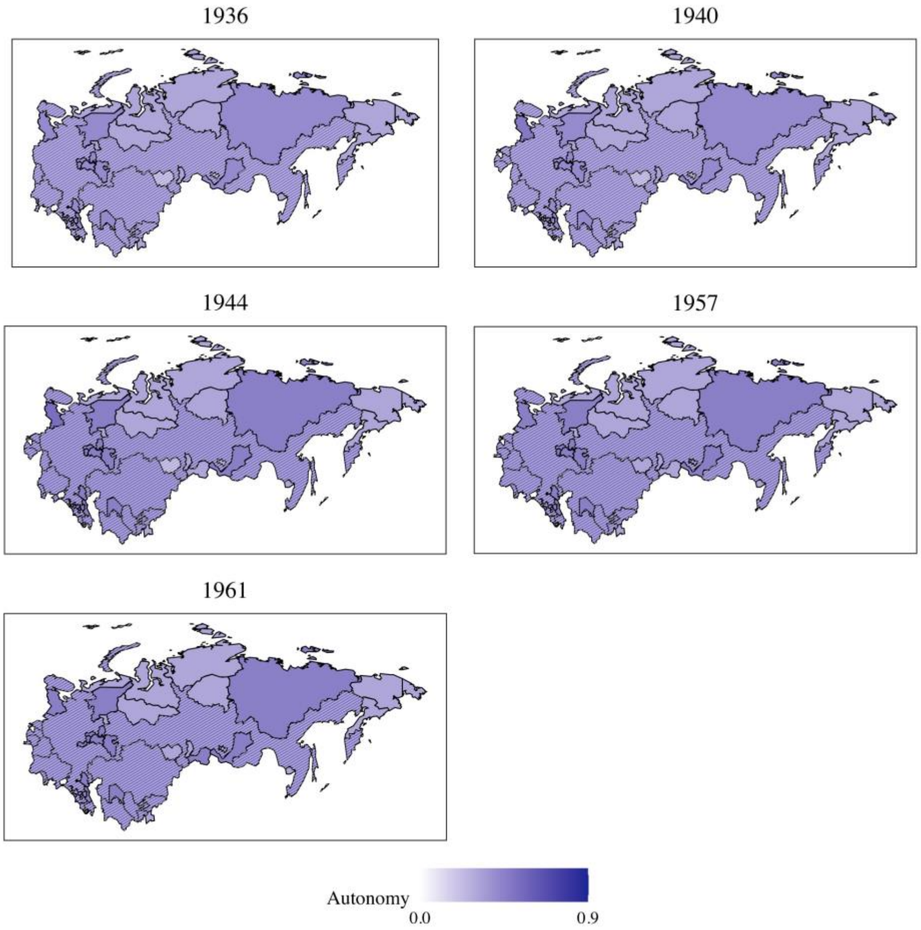

Figure A16. Administrative units and their degree of territorial autonomy in the Soviet Union, 1936-1961.

USSR, 1977-1989

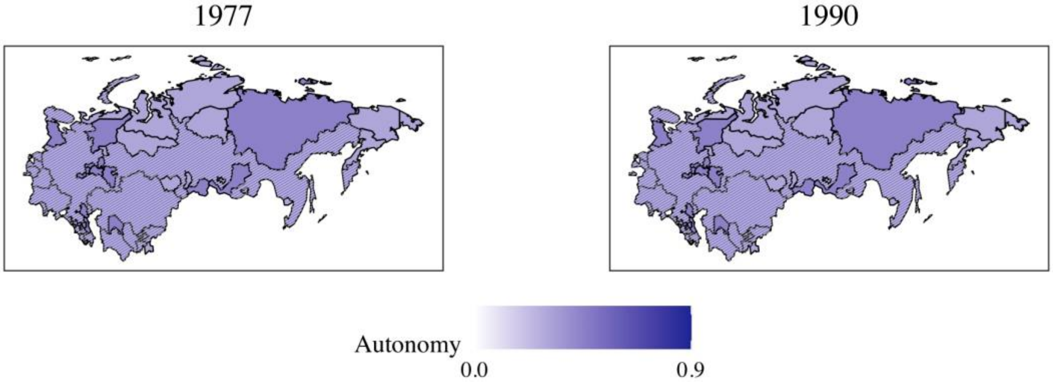

Figure A17. Administrative units and their degree of territorial autonomy in the Soviet Union, 1977-1990.

General information

[Federal or decentralized | Autonomous territories]: The Soviet Union is federal. It is divided into several Soviet Socialist Republics. There are ethnic autonomous areas at different levels: Autonomous Socialist Republics, Autonomous Provinces, and Autonomous Areas. *(Arts. 70; 82; 86; 74)*

Autonomous Oblast and Okrug

- **Policy Autonomy: Institutional Depth [2]:** Each autonomous oblast and okrug is subordinate to the higher-level Union Republic, which enacts a law on an autonomous province, whose law is superior and whose constitution determines their power. The higher organ of power can also annul decisions and resolutions of the autonomous oblast and okrug. The executive organs of the autonomous republics are accountable to the superior level as well. **Policy Scope [2: Economy, Culture, Welfare, Judiciary]:** The autonomous oblast and okrug direct the government organs that are subordinate to them, ensure public order, the observance of the laws, protect citizen rights, direct local economic and cultural affairs and draw up and approve local budgets. They are also responsible for land use, nature protection, construction, the utilization of labor resources, the production of consumer goods, and socio-cultural services. They can furthermore propose plans to the higher levels. It appears that their policy scope can be limited by the higher levels, including the Union Republics and Autonomous Republics. They have their own courts. *(Arts. 86; 88; 141; 146; 147; 148; 150; 151)*
- **Financial Autonomy: Taxing competencies [0]:** Based on the fact that the establishment of taxes is a federal competence and that nowhere are any other fiscal powers specified explicitly, it appears that the autonomous oblast and okrug have no fiscal authority. Rather, they seem to depend on redistribution from the centre. **Borrowing competencies [0]:** Based on the fact that the establishment of revenue and the distribution of the budget are federal competences and that nowhere are any other borrowing powers specified explicitly, it appears that the autonomous oblast and okrug have no borrowing authority. Rather, they seem to depend on redistribution from the centre. **Fiscal guarantees [0].** *(Arts. 74)*
- **Political Autonomy: Legislature [2]:** Each autonomous oblast and okrug has a Soviet of People's Deputies which is directly elected. **Executive [1]:** Each Local Soviet of People's Deputies elects an executive committee. The executive is also accountable to the next higher level, i.e., to the Union or Autonomous Republic above. *(Arts. 89-101; 145; 149; 150)*

Autonomous Republics

- **Policy Autonomy: Institutional Depth [2]:** Each autonomous republic has its own constitution which has to be in full conformity with the constitution of the Union Republic above it. The autonomous republics are supervised by their higher-level union republics, which !!!!!!confirms their constitutions and defines their territorial boundaries (////however, their consent is required). The council of ministers of the higher level entity can further suspend their decisions directly.!!!! **Policy Scope [3: Economy, Culture, Welfare, Judiciary, Institutions, Residual powers]:** It appears that the Autonomous Republics have a similar policy scope as the Union Republics, but without the residual powers and rights to secession. They are explicitly given the tasks of ensuring integrated economic and social development, implementing higher-level decisions, supervising enterprises and local organizations. *(Arts. 73; 74; 76; 77; 79; 80; 84; 137; 151)*
- **Financial Autonomy: Taxing competencies [0]:** Based on the fact that the establishment of taxes is a federal competence and that nowhere are any other fiscal powers specified explicitly, it appears that the Autonomous Republics have no fiscal authority. Rather, they seem to depend on redistribution from the centre. **Borrowing competencies [0]:** Based on the fact that the establishment of revenue and the distribution of the budget are federal competences and that nowhere are any other borrowing powers

specified explicitly, it appears that the Autonomous Republics have no borrowing authority. Rather, they seem to depend on redistribution from the centre. **Fiscal guarantees [0].** *(Arts. 74)*

- **Political Autonomy: Legislature [2]:** Each Autonomous Republic directly elects a Supreme Soviet, which is its highest legislative organ. **Executive [2]:** Each Supreme Soviet of an autonomous republic elects its Presidium and appoints a council of ministers. *(Arts. 89-101; 143; 144)*

Union Republics

- **Policy Autonomy: Institutional Depth [2]:** The federal government exercises oversight over the Union Republics to ensure the conformity of their constitution with the overall constitution. The federal government has to approve any border changes (although the approval of the affected the republics is required). In addition, in case of conflict, Union law prevails. **Policy Scope [4: Economy, Culture, Welfare, Police, Judiciary, Institutions, Residual powers, Secession]:** The division of powers between the Soviet Union and the Union Republics is made via a list of competences given to the federal government and the provision that the republics have residual powers. The exclusive competences of the federal state are the admittance of new republics, confirming changes to the union boundaries, establishing general principles for subordinate organs of power, ensuring the unity of legislative regulation within the USSR, a unified socio-economic policy, the direction of the economic system, the determination of the basic directions of scientific-technical progress, natural resources, the formulation of an integrated state budget, monetary policy, credit policy, establishing taxes and revenues, the price and wage policy, foreign policy, defence, and security, foreign trade, and 'other questions of all-union importance'. Each union republic has its own constitution, the right to secede from the USSR, ensures integrated economic and social development within its territory, implements higher-level decisions, supervises the local economy, determines its territorial and administrative subdivision, and has the right to enter into foreign relations. *(Arts. 3; 73; 74; 76; 77; 78; 79; 80; 137; 151)*
- **Financial Autonomy: Taxing competencies [0]:** Based on the fact that the establishment of taxes is a federal competence and that nowhere are any other fiscal powers specified explicitly, it appears that the Union Republics have no fiscal authority. Rather, they seem to depend on redistribution from the centre. **Borrowing competencies [0]:** Based on the fact that the establishment of revenue and the distribution of the budget are federal competences and that nowhere are any other borrowing powers specified explicitly, it appears that the Union Republics have no borrowing authority. Rather, they seem to depend on redistribution from the centre. **Fiscal guarantees [0].** *(Arts. 74)*
- **Political Autonomy: Legislature [2]:** Each union republic directly elects its Supreme Soviet. **Executive [2]:** Each Supreme Soviet of an autonomous republic elects its Presidium and appoints a council of ministers. *(Arts. 89-101; 137; 138; 139)*

Russia, 1990

General information

[Federal or decentralized | Autonomous territories]: Russia is a federation. There are autonomous oblasts and autonomous okrugs. *(Arts. 1; 3)*

Republics

- **Policy Autonomy: Institutional Depth [2]:** The constitution of each republic has to conform to the Russian one. Russian laws are of superior legal standing. **Policy Scope [4: Economy, Culture, Welfare, Police, Judiciary, Institutions, Residual powers]:** The division of powers between Russia and Republics is made via a list of competences given to the federal government and the provision that the republics have residual powers. Russia has authority over the federated structure, the general government principles, general principles of economic, ecological, social, cultural and ethnic development, the establishment of the market, financial, monetary, credit and customs regulations, price policy, the federal budget, federal taxes and duties, federal funds for regional development, power engineering, railways, communications, foreign policy, war and peace, defense and security, basic judicial administration, standards and measures, and other things. Republics have their own constitutions. The joint jurisdiction includes the republican constitution, freedoms and rights, law and order, resources, culture, education, public health, social security, catastrophe management, general taxation and duties, labor, land, housing, resource management, the judicial system, the protection of native land and habitats, local self-government. *(Arts. 72; 78; 81.1)*
- **Financial Autonomy: Taxing competencies [2]:** Republics are given some vaguely-defined tax autonomy. **Borrowing competencies [0]:** The constitution does not award any borrowing autonomy to the republics. **Fiscal guarantees [0].** *(Arts. 81.1)*
- **Political Autonomy: Legislature [2]:** Republics have their own congresses of people's deputies and supreme soviets. **Executive [2]:** Each Republic has its own President, elected according to its constitution. It also has a Council of Ministers formed by its Supreme Soviet. *(Arts. 85; 86.1; 131; 132.2; 133)*

Autonomous Okrugs and Oblasts

- **Policy Autonomy: Institutional Depth [2]:** The basic legislation of each autonomous okrug and oblast has to conform to the Russian one. Russian laws are of superior legal standing. **Policy Scope [3: Economy, Culture, Welfare]:** Joint jurisdiction that the autonomous okrugs and oblasts have include the conformity of their legislation instruments, local self-government, the general principles of taxation, habitats and lifestyles of small ethnic communities, housing, land, water and forest, resources, education, culture, public health, social security, quarantines, and others. *(Arts. 82; 83; 84.1)*
- **Financial Autonomy: Taxing competencies [2]:** Autonomous okrugs and oblasts are given some vaguely-defined tax autonomy. **Borrowing competencies [0]:** The constitution does not award any borrowing autonomy to the autonomous oblasts and okrugs. **Fiscal guarantees [0].** *(Arts. 84.1)*
- **Political Autonomy: Legislature [2]:** Autonomous okrugs and oblasts have their own congresses of people's deputies and supreme soviets. **Executive [1]:** Each Autonomous Okrug and Oblast has its own executive body accountable to the corresponding soviet of people's deputies and also to superior executive bodies. *(Arts. 85; 86.1; 136.1; 136.3; 136.4)*

Krays and oblasts

- **Policy Autonomy: Institutional Depth [2]:** The basic legislation of each kray and oblast has to conform to the Russian one. Russian laws are of superior legal standing. **Policy Scope [3: Economy, Culture, Welfare]:** Joint jurisdiction that the autonomous okrugs and oblasts have include the conformity of their legislation instruments, local self-government, the general principles of taxation, habitats and lifestyles of small ethnic communities, housing, land, water and forest, resources, education, culture, public health, social security, quarantines, and others. *(Arts. 84.10; 84.11; 84.7; 84.8)*
- **Financial Autonomy: Taxing competencies [2]:** Krays and oblasts are given some vaguely-defined tax autonomy. **Borrowing competencies [0]:** The constitution does not award any borrowing autonomy to the krays and oblasts. **Fiscal guarantees [0].** *(Arts. 84.11)*
- **Political Autonomy: Legislature [2]:** Republics have their own congresses of people's deputies and supreme soviets. **Executive [1]:** Each Kray and Oblast has its own executive body accountable to the corresponding soviet of people's deputies and also to superior executive bodies. *(Arts. 85; 86.1; 136.1; 136.3; 136.4)*

<u>Russia, 1991</u>

General information

[Federal or decentralized | Autonomous territories]: Russia is a federation. There are autonomous oblasts and autonomous okrugs. *(Arts. 1; 3)*

Republics

- **Policy Autonomy: Institutional Depth [2]:** The constitution of each republic has to conform to the Russian one. Russian laws are of superior legal standing. **Policy Scope [4: Economy, Culture, Welfare, Police, Judiciary, Institutions, Residual powers]:** The division of powers between Russia and Republics is made via a list of competences given to the federal government and the provision that the republics have residual powers. Russia has authority over the federated structure, the general government principles, general principles of economic, ecological, social, cultural and ethnic development, the establishment of the market, financial, monetary, credit and customs regulations, price policy, the federal budget, federal taxes and duties, federal funds for regional development, power engineering, railways, communications, foreign policy, war and peace, defense and security, basic judicial administration, standards and measures, and other things. Republics have their own constitutions. The joint jurisdiction includes the republican constitution, freedoms and rights, law and order, resources, culture, education, public health, social security, catastrophe management, general taxation and duties, labor, land, housing, resource management, the judicial system, the protection of native land and habitats, local self-government. *(Arts. 72; 78; 81.1)*
- **Financial Autonomy: Taxing competencies [2]:** Republics are given some vaguely-defined tax autonomy. **Borrowing competencies [0]:** The constitution does not award any borrowing autonomy to the republics. **Fiscal guarantees [0].** *(Arts. 81.1)*
- **Political Autonomy: Legislature [2]:** Republics have their own congresses of people's deputies and supreme soviets. **Executive [2]:** Each Republic has its own President, elected according to its constitution. It also has a Council of Ministers formed by its Supreme Soviet. *(Arts. 85; 86.1; 131; 132.2; 133)*

Autonomous Okrugs and Oblasts

- **Policy Autonomy: Institutional Depth [2]:** The basic legislation of each autonomous okrug and oblast has to conform to the Russian one. Russian laws are of superior legal standing. **Policy Scope [3: Economy, Culture, Welfare]:** Joint jurisdiction that the autonomous okrugs and oblasts have include the conformity of their legislation instruments, local self-government, the general principles of taxation, habitats and lifestyles of small ethnic communities, housing, land, water and forest, resources, education, culture, public health, social security, quarantines, and others. *(Arts. 82; 83; 84.1)*

- **Financial Autonomy: Taxing competencies [2]:** Autonomous okrugs and oblasts are given some vaguely-defined tax autonomy. **Borrowing competencies [0]:** The constitution does not award any borrowing autonomy to the autonomous oblasts and okrugs. **Fiscal guarantees [0].** *(Arts. 84.1)*
- **Political Autonomy: Legislature [2]:** Autonomous okrugs and oblasts have their own congresses of people's deputies and supreme soviets. **Executive [1]:** Each Autonomous Okrug and Oblast has its own executive body accountable to the corresponding soviet of people's deputies and also to superior executive bodies. *(Arts. 85; 86.1; 136.1; 136.3; 136.4)*

Krays and oblasts

- **Policy Autonomy: Institutional Depth [2]:** The basic legislation of each kray and oblast has to conform to the Russian one. Russian laws are of superior legal standing. **Policy Scope [3: Economy, Culture, Welfare]:** Joint jurisdiction that the autonomous okrugs and oblasts have include the conformity of their legislation instruments, local self-government, the general principles of taxation, habitats and lifestyles of small ethnic communities, housing, land, water and forest, resources, education, culture, public health, social security, quarantines, and others. *(Arts. 84.10; 84.11; 84.7; 84.8)*
- **Financial Autonomy: Taxing competencies [2]:** Krays and oblasts are given some vaguely-defined tax autonomy. **Borrowing competencies [0]:** The constitution does not award any borrowing autonomy to the krays and oblasts. **Fiscal guarantees [0].** *(Arts. 84.11)*
- **Political Autonomy: Legislature [2]:** Republics have their own congresses of people's deputies and supreme soviets. **Executive [1]:** Each Kray and Oblast has its own executive body accountable to the corresponding soviet of people's deputies and also to superior executive bodies. *(Arts. 85; 86.1; 136.1; 136.3; 136.4)*

Russia, 2011-2018

General information

[Federal or decentralized | Autonomous territories]: Russia is federal. It consists of republics, krays, oblasts, cities of federal significance, an autonomous oblast and autonomous okrugs. There exist several smaller and larger autonomous units, which, however, also serve as first-level administrative units. *(Arts. 1.1; 5; 5M65; 76.5)*

Federal Subjects

- **Policy Autonomy: Institutional Depth [2]:** The Russian Federal subjects include the republics (which have a differentiated legal treatment), the oblasts/provinces, the kraya/territories, the autonomous okrugs/districts, one autonomous oblast/province, and two federal cities (Moscow and St. Petersburg). The federation treaty gives the others subjects less institutional depth than to the republics, in particular putting their legislation below the national one. Constitutionally, they have equal constitutional status and with the exception of the republics are governed by a statute. Each boundary change requires the assent of the federal subject in question. In contrast to the other subjects, the autonomous okrugs are supervised jointly by the federal government and another federal subject; this does not seem to legally change their institutional depth, however. Since 2000, the institutional depth of the republics is more circumscribed, however, as the Russian president now has the authority to dissolve the parliaments of all federal subjects and suspend their decisions while waiting for court adjudication. **Policy Scope [3: Economy, Culture, Welfare, Police, Judiciary, Institutions, Residual powers]:** The other federal subjects have the same policy competences as the republics. *(Arts. 65; 71; 72; 73; Federation Treaty of 1992)*
- **Financial Autonomy: Taxing competencies [3]:** All federal subjects have the same fiscal autonomy as the republics. **Borrowing competencies [2]:** All federal subjects have the same borrowing autonomy as the republics. **Fiscal guarantees [0].** *(Arts. 71; Law No. 145/1998, Arts . 92, 95; Law on the Foundation of Budgetary Rights, 1993)*
- **Political Autonomy: Legislature [2]:** Federal assemblies, including those of the all subjects that are not republics, are directly elected. **Executive [1]:** The governor of the federal subjects is chosen in the same way as the president of the republics.

Republics

- **Policy Autonomy: Institutional Depth [2]:** The federation treaty of 1992 gives the republics more institutional depth than the other subjects, denoting them as ‘sovereign’. Furthermore, it puts republican and federal law on an equal basis, which suggests that the central state has no veto right over their legislation. The Russian Federal subjects all have equal constitutional status, but the republics have still legally attained some special powers. In contrast to the other subjects, the republics have their own constitutions. Each boundary change requires the assent of the republic in question. Since 2000, the institutional depth of the republics is more circumscribed, however, as the Russian president now has the authority to dissolve the parliaments of all federal subjects and suspend their decisions while waiting for court adjudication. **Policy Scope [3: Economy, Culture, Welfare, Police, Judiciary, Institutions, Residual powers]:** The Federation Treaty of 1992 gives the republics further policy competences than to the other subjects. In particular, they can hold direct elections for the presidents, have control over land and natural resources, property rights and trade, and are allowed to declare a state of emergency. The

division of competences is constitutionally-enshrined through a list of exclusive federal competences, concurrent federal-subject competences, and residual competences for all federal subjects. The federal government has exclusive competence over the jurisdictional architecture of the federation, the single market, monetary policy, foreign and defense policy, trade policy, the legal system, accounting standards, and citizenship and immigration. It also sets the framework legislation of the economy, the environment, the socio-cultural fabric of Russia, and energy policy. It is also responsible for federation-wide infrastructure in transport, communications, and energy. Concurrent policies are very numerous and include natural resource management, the environment, the coordination of external economic relations, emergency services, taxation, education, science, culture and sports, coordination of health and social security, the judiciary and law enforcement, minority rights, the protection of rights and freedoms, law and order, and local government. Each subject determines its own internal organization, although the federal state lays down basic rules through laws. Since 2000, some powers have been attributed to the new higher tier of federal okrugs, however, including the supervision of law and order. *(Arts. 65; 71; 72; 73; Federation Treaty of 1992)*

- **Financial Autonomy: Taxing competencies [3]:** Taxation is concurrent between the federation and all federal subjects, including the republics. Exclusively federal taxes are the value added tax, alcohol and vehicle excises, taxes on bank and insurance profits, taxes on currency exchange and securities, and customs duties. The federal government also sets the base and rate of shared taxes, including personal and corporate income taxes, and excise taxes (except for motor vehicles and alcohol). Federal subjects set the rate of property, roads, gambling, transport, and land as well as corporate profits tax (from 17.5 down to 13.5). **Borrowing competencies [2]:** Borrowing of all federal subjects, including the republics, is tied to specific conditions: Federal authorization is required for bonds issued by subject governments, and there are registration disclosure requirements. For other components, no prior approval is needed. Their budget deficit can only be 10 percent of budget revenues, the overall level of debt is restricted to 15 percent of expenditures, and borrowing is constrained to capital investment (except to refinance old debt). **Fiscal guarantees [0].** *(Arts. 71; Law No. 145/1998, Arts . 92, 95; Law on the Foundation of Budgetary Rights, 1993)*
- **Political Autonomy: Legislature [2]:** Federal assemblies, including those of the republics, are directly elected. **Executive [1]:** The assembly votes on a candidate for president that is nominated by the Russian president. If it rejects a candidate for three times, the president can dissolve the assembly. This was coded as a dual executive.

Tatarstan

- **Policy Autonomy: Institutional Depth [3]:** Tatarstan's autonomy goes beyond that of the other republics, as it has signed a bilateral treaty with the Russian government in 1994. **Policy Scope [3: Economy, Culture, Welfare, Police, Judiciary, Institutions, Residual powers]:** Tatarstan has a wider policy scope than the other republics through its treaty with the central government, which additionally gives it authority over foreign economic policy, the protection of civil liberties and rights, amnesty to individuals convicted by regional courts, and symbolic citizenship provisions (e.g. the passport language). *(Arts. 65; 71; 72; 73; Federation Treaty of 1992)*
- **Financial Autonomy: Taxing competencies [3]:** Tatarstan has the same fiscal autonomy as the other federal subjects. **Borrowing competencies [2]:** Tatarstan has the same borrowing autonomy as the other federal subjects. **Fiscal guarantees [0].** *(Arts. 71; Law No. 145/1998, Arts . 92, 95; Law on the Foundation of Budgetary Rights, 1993)*
- **Political Autonomy: Legislature [2]:** Federal assemblies, including the one of Tatarstan, are directly elected. **Executive [1]:** The president of Tatarstan is chosen in the same way as the other federal subjects.

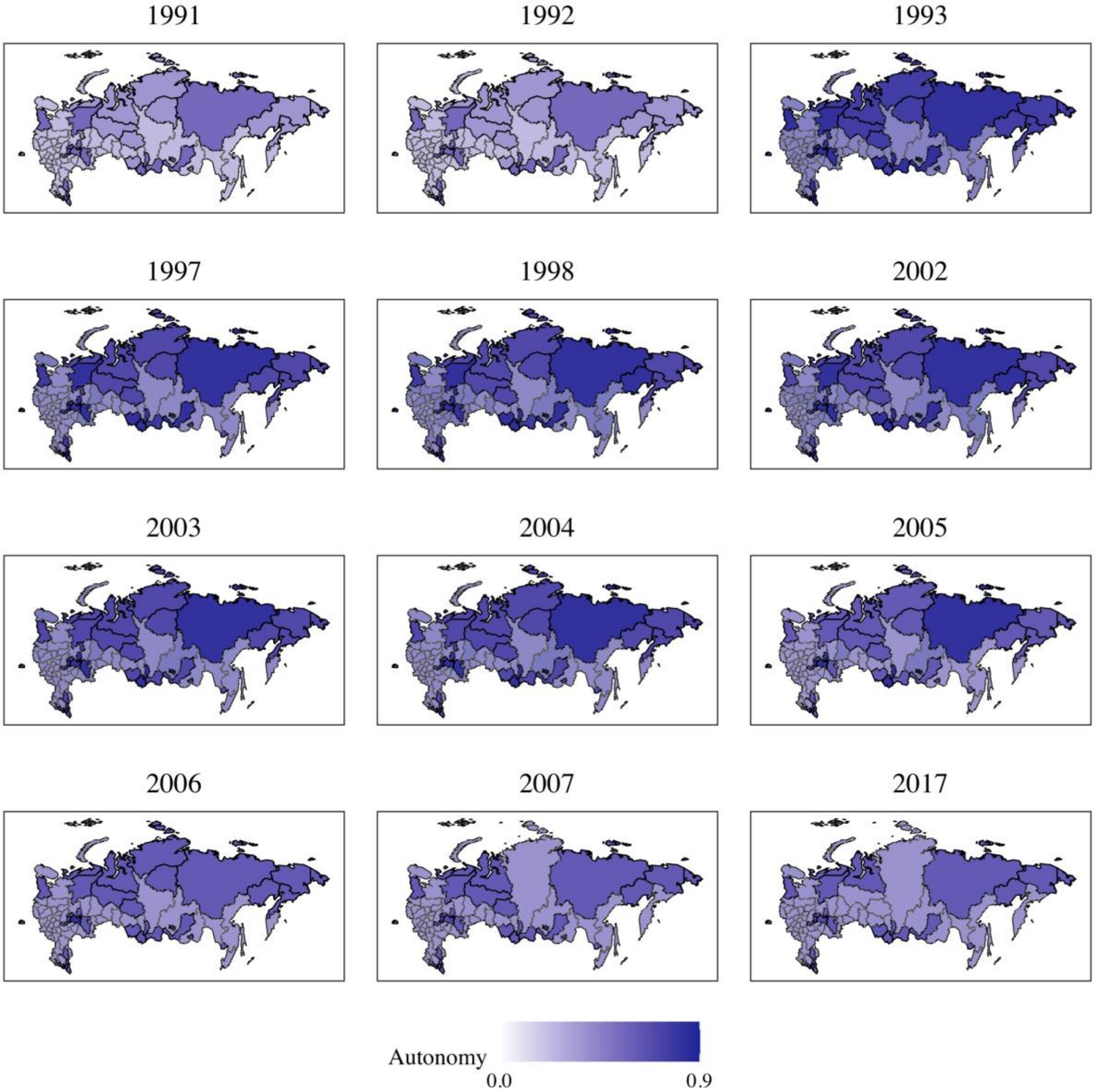

Figure A18. Administrative units and their degree of territorial autonomy in Russia, 1991-2017.

Appendix C.6: Complete list of autonomy scores in our sample

Poland
Provinces [0.27] (1944-1946)
Provinces [0.27] (1947-1951)
Municipalities [0.39] (1997-2017)
Czechoslovakia
Slovakia [0.31] (1948-1959)
Slovakia [0.36] (1960-1967)
Czech Socialist Republic [0.66] (1968-1970)
Slovak Socialist Republic [0.66] (1968-1970)
Czech Socialist Republic [0.57] (1971-1989)
Slovak Socialist Republic [0.57] (1971-1989)
Czech Socialist Republic [0.73] (1990-1992)
Slovak Socialist Republic [0.73] (1990-1992)
Albania
Regions [0.25] (2000-2017)
Macedonia
Municipalities [0.43] (1991-2000)
Municipalities [0.47] (2001-2004)
Municipalities [0.52] (2005-2017)
Croatia
Autonomous Districts of Glina and Knin [0.49] (1992-1996)
Yugoslavia
Banovina of Croatia [0.48] (1939-1945)
Autonomous Kosovo-Metohijan region [0.33] (1946)
Autonomous Province of Vojvodina [0.36] (1946)
People's Republics [0.48] (1946-1952)
Autonomous Kosovo-Metohijan region [0.34] (1947-1952)
Autonomous Province of Vojvodina [0.38] (1947-1952)
Autonomous Kosovo-Metohijan region [0.34] (1953-1962)
Autonomous Province of Vojvodina [0.38] (1953-1962)
People's Republics [0.55] (1953-1962)
Autonomous Provinces [0.43] (1963-1967)
People's Republics [0.73] (1963-1970)
Autonomous Provinces [0.49] (1968-1970)
Autonomous Provinces [0.56] (1971-1973)
People's Republics [0.8] (1971-1973)
Autonomous Provinces [0.8] (1974-1987)
People's Republics [0.8] (1974-1987)
Autonomous Provinces [0.7] (1988-1989)
People's Republics [0.7] (1988-1991)
Autonomous Provinces [0.41] (1990-1991)
Montenegro [0.8] (1992-2002)
Serbia [0.8] (1992-2002)
Autonomous Province of Kosovo [0.7] (2003-2005)
Autonomous Province of Vojvodina [0.41] (2003-2005)
Montenegro [0.95] (2003-2005)
Serbia [0.95] (2003-2005)
Serbia
Autonomous Province of Kosovo [0.81] (2006-2007)
Autonomous Province of Vojvodina [0.52] (2006-2017)
Bosnia
Cantons [0.72] (1995-2017)
Federation [0.87] (1995-2017)
Republika Srpska [0.87] (1995-2017)
Brcko [0.66] (1999-2007)
Brcko [0.78] (2008-2017)
Moldova
Gagauzia [0.39] (1995-2017)
Transnistria [0.28] (2005-2017)
Romania
Magyar Autonomous Region [0.27] (1950-1959)
Magyar Autonomous Region [0.27] (1960-1964)
Magyar Autonomous Region [0.27] (1965-1968)
Counties [0.34] (1991-2002)
Counties [0.34] (2003-2017)

USSR
Autonomous Oblast and Okrug [0.31] (1936-1976)
Autonomous Republics [0.41] (1936-1943)
Union Republics [0.48] (1936-1943)
Autonomous Republics [0.46] (1944-1976)
Union Republics [0.52] (1944-1976)
Autonomous Oblast and Okrug [0.31] (1977-1990)
Autonomous Republics [0.44] (1977-1990)
Union Republics [0.5] (1977-1990)
Russia
Autonomous Okrugs and Oblasts [0.37] (1991)
Krays and oblasts [0.37] (1991)
Republics [0.55] (1991)
Autonomous Okrugs and Oblasts [0.37] (1992)
Krays and oblasts [0.37] (1992)
Republics [0.55] (1992)
Federal Subjects [0.76] (1993-1995)
Federal Subjects with a bilateral treaty [0.82] (1993-2005)
Republics [0.82] (1993-1994)
Republics with a bilateral treaty [0.82] (1993-2005)
Bashkortostan [0.88] (1994-1995)
Tatarstan [0.88] (1994-1995)
Republics [0.82] (1995)
Bashkortostan [0.82] (1996-2004)
Federal Subjects [0.7] (1996-1999)
Republics [0.77] (1996-1997)
Tatarstan [0.82] (1996-2006)
Republics [0.77] (1998-1999)
Federal Subjects [0.7] (2000-2004)
Republics [0.7] (2000-2004)
Federal Subjects [0.62] (2005-2017)
Republics [0.62] (2005-2006)
Republics [0.62] (2007-2017)
Tatarstan [0.69] (2007-2017)
Ukraine
Autonomous Republic of the Crimea [0.43] (1991)
Republic of the Crimea [0.41] (1992-1994)
Autonomous Republic of the Crimea [0.33] (1995)
Autonomous Republic of the Crimea [0.31] (1996-1997)
Autonomous Republic of the Crimea [0.39] (1998-2017)
Georgia
Abkhazia [0.41] (1991)
Adjaria [0.41] (1991)
South Ossetia [0.37] (1991)
Abkhazia [0.46] (1992-1994)
Adjaria [0.46] (1992-1994)
Abkhazia [0.38] (1995-1999)
Adjaria [0.46] (1995-2003)
Abkhazia [0.37] (2000-2004)
Adjaria [0.28] (2004-2017)
Abkhazia [0.37] (2005-2017)
Azerbaijan
Nagorno-Karabakh [0.39] (1991-1994)
Nakhchivan [0.41] (1991-1994)
Nakhchivan [0.39] (1995-2017)
Tajikistan
Gorno-Badakhshan [0.41] (1991-1993)
Gorno-Badakhshan [0.51] (1994)
Gorno-Badakhshan [0.51] (1995-2006)
Gorno-Badakhshan [0.51] (2007-2017)
Uzbekistan
Karakalpakstan [0.41] (1991)
Karakalpakstan [0.47] (1992)
Karakalpakstan [0.44] (1993-2017)

Appendix D: Illustrative cases (CEE-FSU sample)

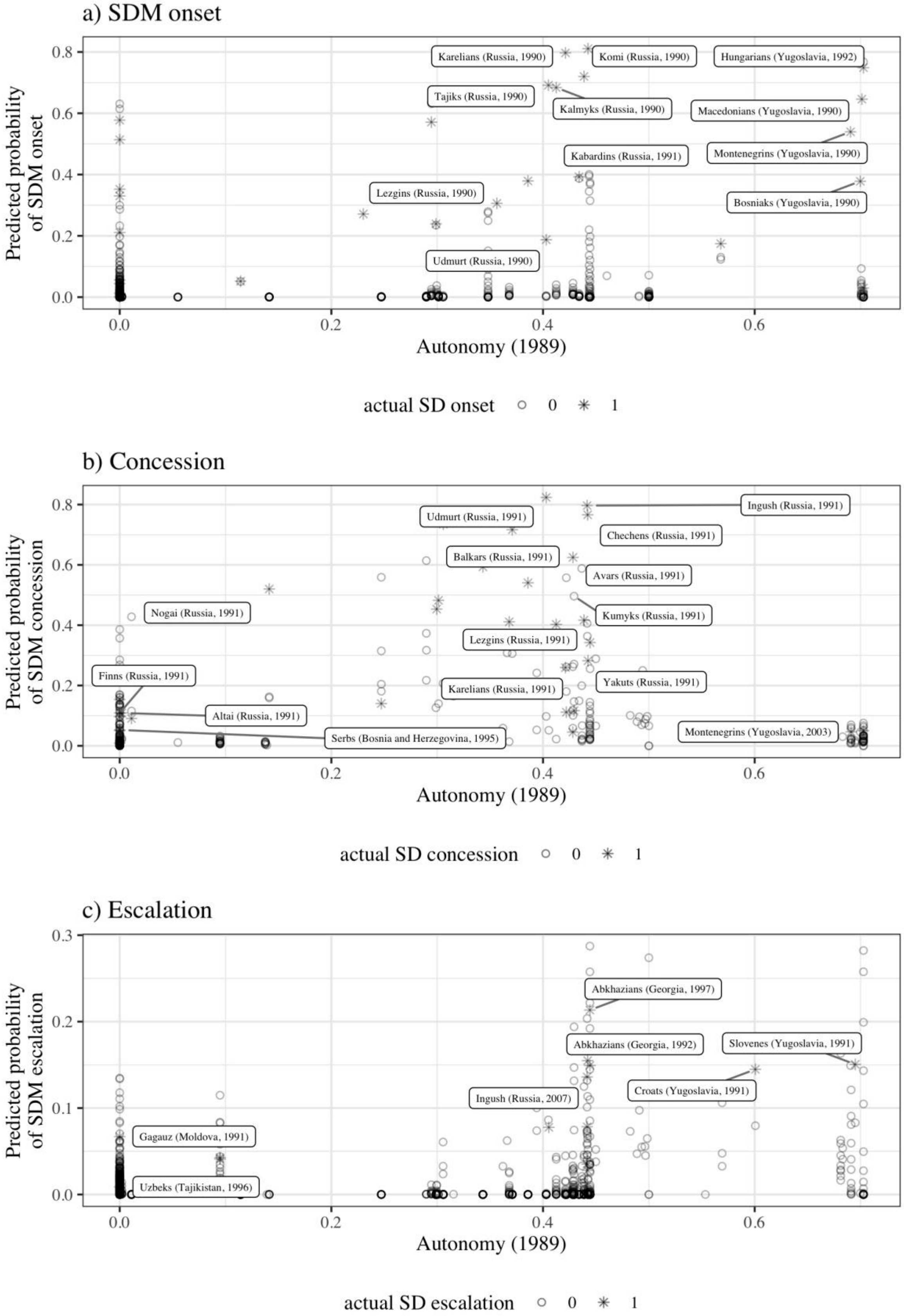

Figure A19. Fitted probabilities (models 5, 6, and 7) and actual cases.

Appendix E: Robustness checks (CEE-FSU sample)

In this appendix, we provide robustness checks for our analyses focused on our CEE-FSU sample (table 3 in the main article text).[14] In a first step, we probe the robustness of our findings to **different 'time windows'** (appendix E.1). In our main models, we had included all group years that classify as transition years as coded either by our *transition (0-5)* measure, based on the V-Dem Episodes of Regime Transition Dataset (Maerz et al., 2021), or within five years after the initial shock of 1989 (1990-1994). We variably alter this criterion in two ways. First, we include **all group years between 1990 and 2017** in our sample, even if they do not classify as transition periods according to our operationalization. In this temporally more extensive sample that encompasses later years during which the post-Communist successor states were more consolidated, our findings remain similar. The most important change applies to the association between *autonomy (1989)* and SDM onsets. While this association remains positive, it loses its statistical significance. Our other findings remain statistically significant but are slightly weaker in magnitude, which is consistent with our theory. Second, we conversely **restrict our sample further, by limiting it to the five years immediately following the initial shock of 1989** (1990-1994). Here, our findings on how *autonomy (1989)* affects SDM onsets and concessions remain comparable to those from our main models. However, the positive association between autonomy and violent SDM escalation loses statistical significance. These findings likely reflect the fact that, during transition periods, autonomous groups may move to initiate SDMs relatively quickly. However, the escalation of such SDMs may be subject to more slow-moving bargaining processes. One example is the escalation of the Albanians' SDM in Serbia and Montenegro in 1998, which occurred after the period denoted as transitional by our variable.

In a second step, we probe the robustness of our findings to **alterations to our sample** (appendix E.2). We variably limit our sample to those states with significant minority population shares (above 10%) and to the states in the Former Soviet Union.[15] For the latter, we variable use our original *autonomy (1989)* measure and replace it with a temporally less proximate for autonomy in 1936, the year the Soviet Union's second constitution was instituted (see appendix C.5). In the latter sub-sample, that only encompasses the Former Soviet Union, internal bargaining dynamics played a less substantial role in the initial provision of territorial autonomy than in other cases, such as Yugoslavia and Czechoslovakia. Focusing on these cases thereby helps us guard better against the challenges of endogenous and mobilization-induced autonomy (figure 1, challenges 2a/2b). Reassuringly, our results do not appear sensitive to any of these alterations.

In a third step, we incorporate **additional control variables** (appendix E.3). These aim to capture developments in the post-transition period that might bias our findings as to the post-transition consequences of autonomy transmitted from 1989. A first important concern we address is the role of **lost autonomy**. A particular concern here is that the positive association between autonomy in 1989 and self-determination demands in the post-transition period might not reflect the continuing capabilities it provides to ethnic groups. Instead, it might reflect instances where groups have *lost* their previously high degrees of autonomy in the post-transition period and mobilize to reclaim it (cf. Siroky & Cuffe 2015; Germann & Sambanis 2021). To account for this alternative mechanism, we construct and incorporate a control variable for lost autonomy:

$$lost\ autonomy_{i,c,t} = \begin{cases} autonomy\ (1989)_{i,c,} - autonomy_{i,c,t} & \text{if } autonomy_{i,c,t} < autonomy\ (1989) \\ 0 & \text{otherwise.} \end{cases}$$

Reassuringly, our results remain robust to the inclusion of this control variable. Moreover, while *lost autonomy* is positively associated with SDM onsets and (renewed) concessions, it exhibits a strong negative correlation with the violent escalation of SDMs in our sample. This may reflect the fact that, in the post-transition period, autonomy was less likely to be withdrawn from groups that were in a position to violently resist such efforts. Moreover, several cases where lost autonomy sparked the escalation of SDMs (such as Serbia and Montenegro's Albanians in 1998) drop out of our main sample, as they occur outside the transition period variable whereby we delimit our sample (see above).

Another concern is posed by the **wider bargaining environment facing the newly consolidating post-communist successor states**. Importantly, owing to reputational concerns (Walter 2006), transitioning states might be reluctant to offer autonomy in response to self-determination movements to avoid emboldening other potential challengers, especially if they have new, unconsolidated international borders, as applies in many cases in our CEE-FSU sample. In turn, if governments *are* forced to offer such concessions, this might inspire other groups to make similar demands and initiate SDMs of their own. To account for these processes, we control for the *number of ethnic groups* and for *recent concessions offered to other groups*, using similar operationalizations as previous research (Walter 2006; Bormann & Savun 2018).[16] Reassuringly, the incorporation of these controls does not affect our findings.

[14] Again, we include our standard country- and group-year control variables but omit them from the reported tables. Our R-code in the supplementary material provides the full tables.

[15] In this much more limited sample encompassing only states of the Former Soviet Union, we are forced to omit all country-level controls except for democracy (normalized polity score) due to multicollinearity.

[16] We operationalize this in two steps, equivalently to Bormann and Savun (2018): First, we code group years where a group witnesses another group obtaining autonomy concessions, which are reflected in its degree of autonomy increasing by at least 0.1. In all such years, the value of

Another concern is that some groups mobilized for self-determination during Communist rule, attained autonomy, and resumed mobilization for further concessions in the post-transition period. Thereby, our finding that autonomy in 1989 was associated with subsequent SDM outbreaks may reflect the continuing, if temporally removed, mobilization of such groups. To address this possibility, we control for the (logged) number of **years each group was involved in an SDM before the onset of the 1989 transition**,[17] helping us further address concerns of mobilization-induced autonomy (figure 1, challenge 2b). Reassuringly, our results remain robust. Highlighting the disjuncture between pre- and post-transition periods, and attenuating concerns of reverse causation, this variable even takes a negative association with post-1989 SDM onsets and their violent escalation.

In a fourth step, we probe the robustness of our findings to a different empirical approach. Instead of predicting SDM onsets, concessions, and violent escalation during SDMs with our *autonomy (1989)* variable, we use this variable to **instrument for each group's time-variant degree of autonomy** (appendix E.4). We first provide evidence that autonomy in the post-transition period is ‘sticky’, as shown by the persistent, high correlations between a group's autonomy in a given year between 1990 and 2017 and its autonomy in 1989 (figure A28). Echoing this descriptive finding, our first-stage models indicate that autonomy in 1989 is a highly significant and substantially meaningful predictor of autonomy in the post-transition period. Moreover, our instrumented autonomy variable predicts *SDM onset* and *SDM escalation* in similar ways as our original autonomy (1989) variable. The only difference relates to concessions, where we do not attain a statistically significant effect of our instrumented autonomy variable. In part, this may reflect the fact that groups with high degrees of autonomy in a given year (as opposed to high degrees of autonomy in 1989) are by definition less likely to obtain further, substantial concessions. For example, in the hypothetical cases where a group has the maximum degree of autonomy in a given year (1), it can by definition not obtain further concessions. Overall, the results from this highly dissimilar procedure thereby appear comparable with the results from our main models. This further reassures us of their robustness.

In a final step, we probe two key assumptions whereby we justified our case selection (appendix E.5). First, we have argued that bargaining dynamics over ethnic autonomy were muted before 1989 when the Communist party was the dominant state institution, helping us mitigate the dangers of mobilization-induced autonomy (figure 1, challenge 2b). To probe this assumption, we **examine whether a group's involvement in SDMs predicts changes in its degree of autonomy in the pre-1989 period.** We find a positive association between SDM involvement and changes in a group's autonomy in the pre-1989 period, although reassuringly this has a limited magnitude (SDM involvement is associated with a subsequent increase of autonomy by 0.007 on a scale ranging from 0 to 1). Moreover, this association is driven by pre-1989 SDMs in Czechoslovakia (the Slovaks, who were mobilized in an SDM and obtained substantial concessions in 1948 and 1968) and Yugoslavia (most importantly, the Albanians received concessions in 1963 and 1974, and the Slovenes and Croats in 1971). As we argued in the main article text, bargaining dynamics played a larger role in these cases. Moreover, we excluded these more problematic cases in our models that subset the sample to the Former Soviet Union only (see appendix E.2). Reassuringly, when re-running our analyses of how SDM involvement affects changes in autonomy for groups in the Soviet Union only, we find no substantial or statistically significant association. This reassures us that bargaining dynamics played no major role at least in this subset of cases, which we have highlighted in our argument, and in which our findings remain robust.

Second, we have also argued in a similar fashion that a group’s inherited autonomy should be largely unrelated to its future bargaining power vis-à-vis the respective successor states, helping us to address the challenges of endogenous and mobilization-induced autonomy (figure 1, challenges 2a/2b). To test this assumption, we reverse our main analyses, **probing whether we can predict a group's 'inherited' autonomy in 1989 with its post-transition SDM onsets, obtained concessions, and escalation years**. Consistent with our argument, we do not find a strong explanatory role of these factors.

our *autonomy concession (other group)* variable takes the value 1. In years thereafter, this initially high value decays, with a three-year half-life.

[17] For this purpose, we count the number of years that a group was involved in an ongoing SDM between 1946 (the start of our data) and 1989.

Appendix E.1: Robustness to different 'time windows'

Table A22. Results: different time windows I - all years between 1990 and 2017.

	SDM onset Model 1	Concession Model 2	Escalation during SDM Model 3
Autonomy (1989)	6.437	2.217^{**}	2.855^{***}
	(4.420)	(0.919)	(1.074)
Recent concession			-0.612^{*}
			(0.329)
Constant	-16.448^{*}	7.373	-2.108
	(8.672)	(4.650)	(6.137)
N	1223	585	1192
Log Likelihood	-63.888	-83.060	-75.887
AIC	159.776	192.121	185.774

$^{***}p < .01$; $^{**}p < .05$; $^{*}p < .1$; Country-clustered errors in parentheses. Cubic term for time dependence of dependent variables and control variables included but not reported.

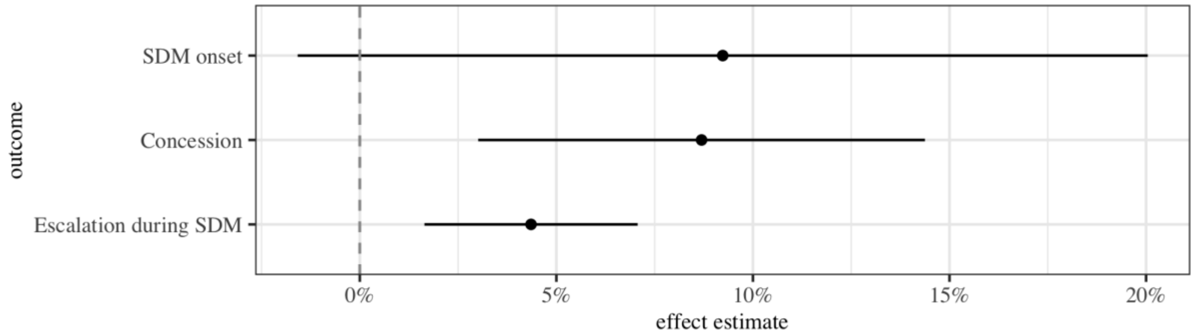

Figure A20. First difference in the predicted probability of SDM onset, concessions during SDM, and escalation during SDM, depending on autonomy in 1989. Based on models 1-3 in table A22.

Table A23. Results: different time windows II - immediate post-transition phase (1990-1994).

	SDM onset Model 1	Concession Model 2	Escalation during SDM Model 3
Autonomy (1989)	3.021^{***}	3.321^{***}	4.575
	(0.969)	(0.883)	(3.391)
Recent concession			2.008^{***}
			(0.731)
Constant	-5.448	32.807	-8.898
	(4.139)	(27.469)	(13.337)
N	170	195	245

$^{***}p < .01$; $^{**}p < .05$; $^{*}p < .1$; Country-clustered errors in parentheses. Cubic term for time dependence of dependent variables and control variables included but not reported.

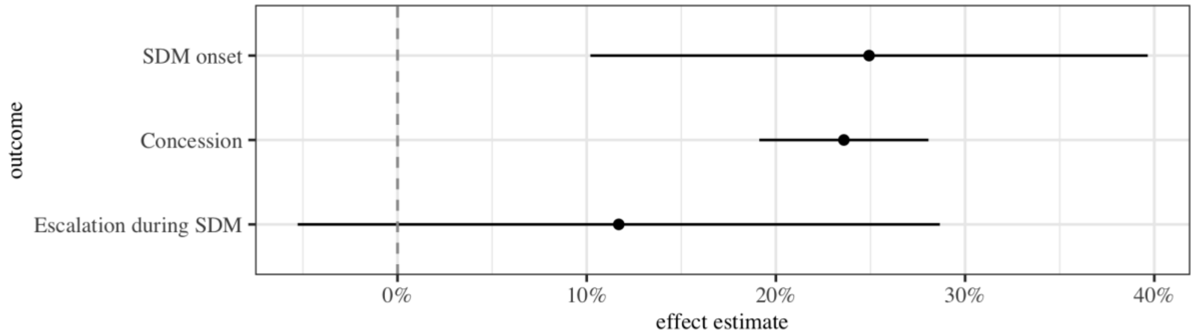

Figure A21. First difference in the predicted probability of SDM onset, concessions during SDM, and escalation during SDM, depending on autonomy in 1989. Based on models 1-3 in table A23.

Appendix E.2: Robustness to sample alterations

Table A24. Results: only multi-ethnic states (at least 10% minority population shares).

	**SDM onset**	**Concession**	**Escalation during SDM**
	**Model 1**	**Model 2**	**Model 3**
Autonomy (1989)	7.835***	2.711**	2.579**
	(2.874)	(1.092)	(1.199)
Recent concession			-0.509
			(0.326)
Constant	-19.301***	9.347	-3.273
	(7.351)	(7.023)	(6.937)
N	780	386	930
Log Likelihood	-55.468	-71.816	-54.959
AIC	142.936	169.632	143.918

***p < .01; **p < .05; *p < .1; Country-clustered errors in parentheses. Cubic term for time dependence of dependent variables and control variables included but not reported.

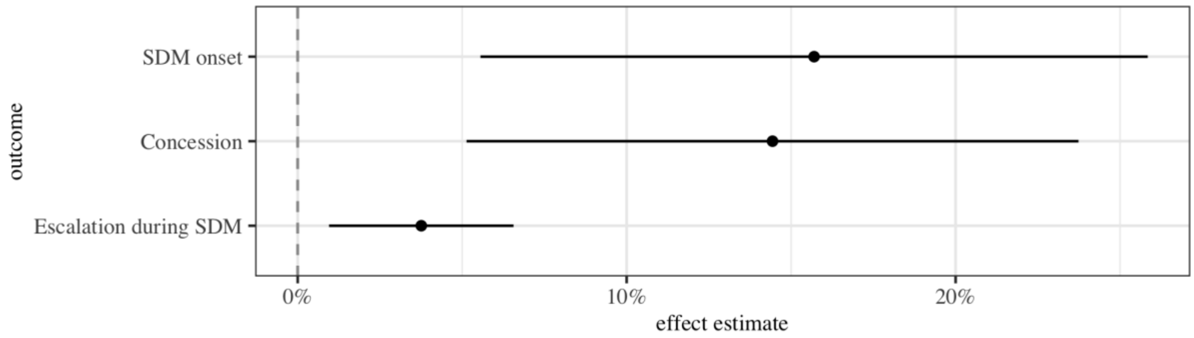

Figure A22. First difference in the predicted probability of SDM onset, concessions during SDM, and escalation during SDM, depending on autonomy in 1989. Based on models 1-3 in table A24.

Table A25. Results: only states in the Former Soviet Union.

	**SDM onset**	**Concession**	**Escalation during SDM**
	**Model 1**	**Model 2**	**Model 3**
Autonomy (1989)	3.603*	2.712***	5.629*
	(1.847)	(0.762)	(3.332)
Recent concession			-0.712**
			(0.285)
Constant	-3.093*	0.410	-8.548**
	(1.833)	(2.054)	(4.118)
N	654	280	771
Log Likelihood	-26.341	-58.421	-45.104
AIC	78.681	136.842	118.208

***p < .01; **p < .05; *p < .1; Country-clustered errors in parentheses. Cubic term for time dependence of dependent variables and control variables included but not reported.

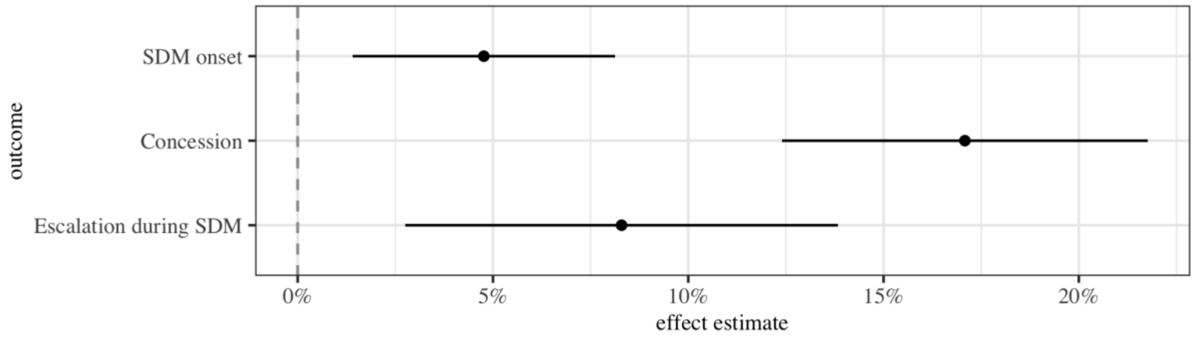

Figure A23. First difference in the predicted probability of SDM onset, concessions during SDM, and escalation during SDM, depending on autonomy in 1989. Based on models 1-3 in table A25.

Table A26. Results: only states in the Former Soviet Union + autonomy measured in 1936.

	SDM onset<br>Model 1	Concession<br>Model 2	Escalation during SDM<br>Model 3
Autonomy (1936)	2.473	1.958**	6.832**
	(1.784)	(0.819)	(3.321)
Recent concession			-0.693**
			(0.288)
Constant	-2.828	1.130	-8.249**
	(1.762)	(1.921)	(4.035)
N	654	280	771
Log Likelihood	-26.837	-59.221	-44.328
AIC	79.675	138.442	116.657

***p < .01; **p < .05; *p < .1; Country-clustered errors in parentheses. Cubic term for time dependence of dependent variables and control variables included but not reported.

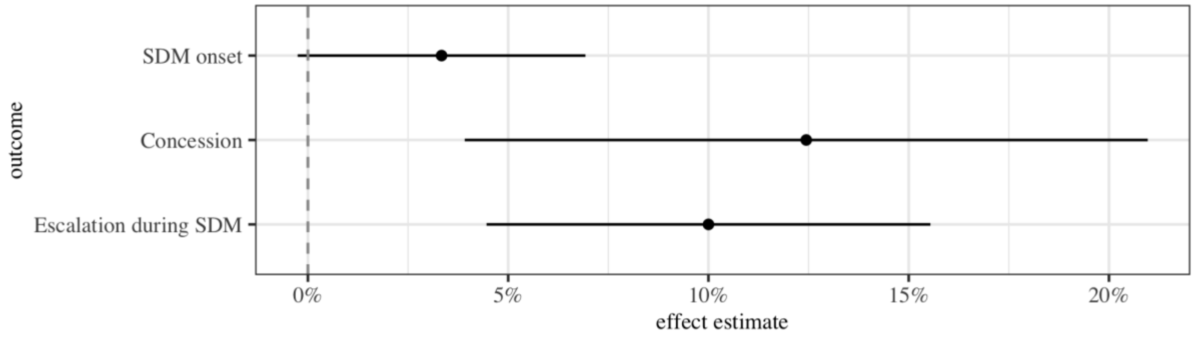

Figure A24. First difference in the predicted probability of SDM onset, concessions during SDM, and escalation during SDM, depending on autonomy in 1989. Based on models 1-3 in table A26.

Appendix E.3: Robustness to additional controls

Table A27. Results: controlling for autonomy loss since 1989.

	SDM onset<br>Model 1	Concession<br>Model 2	Escalation during SDM<br>Model 3
Autonomy (1989)	5.135***	2.450**	5.753**
	(1.941)	(0.989)	(2.482)
Recent concession			-0.831**
			(0.352)
Autonomy loss since 1989	6.402*	1.415	-7.884**
	(3.873)	(1.098)	(3.631)
Constant	-16.328**	9.717	-1.953
	(6.374)	(7.443)	(5.866)
N	879	429	969
Log Likelihood	-61.106	-71.752	-51.898
AIC	156.212	171.504	139.796

***p < .01; **p < .05; *p < .1; Country-clustered errors in parentheses. Cubic term for time dependence of dependent variables and further control variables included but not reported.

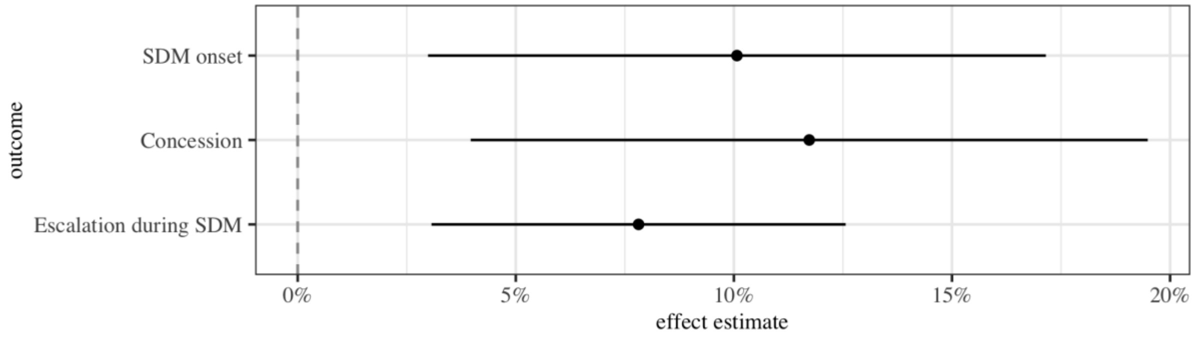

Figure A25. First difference in the predicted probability of SDM onset, concessions during SDM, and escalation during SDM, depending on autonomy in 1989. Based on models 1-3 in table A27.

Table A28. Results: accounting for the wider bargaining environment (number of groups in the same country, autonomy concessions provided to other groups)

	**SDM onset**<br>**Model 1**	**Concession**<br>**Model 2**	**Escalation during SDM**<br>**Model 3**
Autonomy (1989)	6.733**	2.739***	3.505***
	(2.879)	(1.004)	(1.297)
Recent concession			-0.121
			(0.401)
Number of groups	-0.032	-0.054	0.153***
	(0.064)	(0.054)	(0.044)
Autonomy concession (other group)	-1.816*	1.177	-0.913
	(1.028)	(0.759)	(0.640)
Constant	-16.410***	8.050	0.836
	(5.441)	(7.214)	(6.027)
N	879	429	969
Log Likelihood	-61.302	-69.802	-53.294
AIC	158.604	169.605	144.588

***p < .01; **p < .05; *p < .1; Country-clustered errors in parentheses. Cubic term for time dependence of dependent variables and further control variables included but not reported.

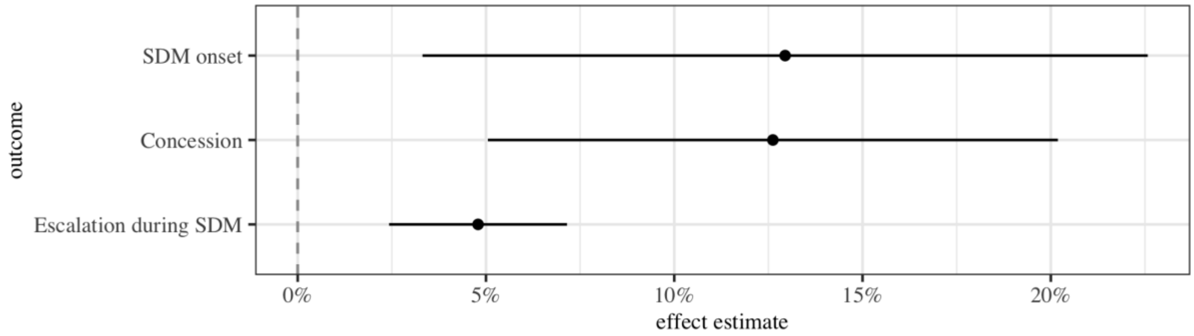

Figure A26. First difference in the predicted probability of SDM onset, concessions during SDM, and escalation during SDM, depending on autonomy in 1989. Based on models 1-3 in table A28.

Table A29. Results: controlling for each group's (logged) number of years of SDM involvement before 1989.

	SDM onset<br>Model 1	Concession<br>Model 2	Escalation during SDM<br>Model 3
Autonomy (1989)	6.240	2.292**	2.546**
	(3.822)	(0.984)	(1.105)
Recent concession			-0.592**
			(0.259)
SDM years before 1989 (log)	-0.094**	0.073***	-0.296***
	(0.041)	(0.020)	(0.108)
Constant	-15.495*	13.526**	0.498
	(7.962)	(6.082)	(6.018)
N	879	429	969
Log Likelihood	-62.135	-64.917	-53.800
AIC	158.270	157.833	143.601

***p < .01; **p < .05; *p < .1; Country-clustered errors in parentheses. Cubic term for time dependence of dependent variables and further control variables included but not reported.

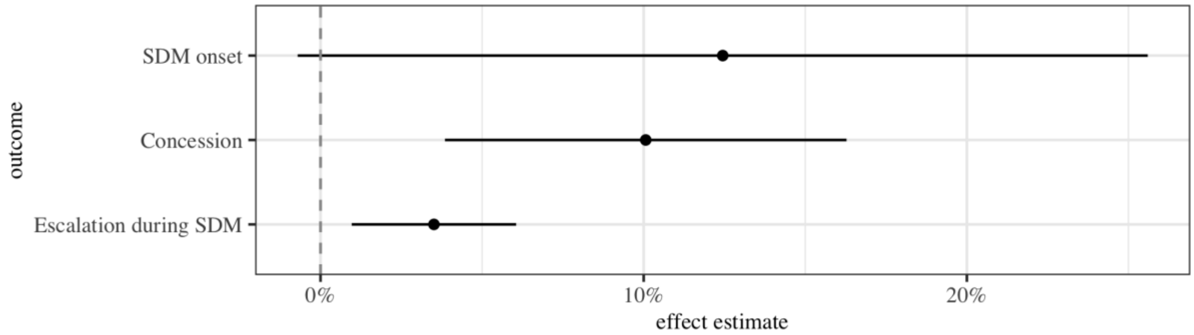

Figure A27. First difference in the predicted probability of SDM onset, concessions during SDM, and escalation during SDM, depending on autonomy in 1989. Based on models 1-3 in table A29.

Appendix E.4: Instrumenting for autonomy with autonomy in 1989

Table A30. Results: Instrumental variable approach.

	model 1		model 2		model 3	
	eq. 1	eq. 2	eq. 1	eq. 2	eq. 1	eq. 2
dependent variable	*Autonomy*	*SD onset*	*Autonomy*	*Concession*	*Autonomy*	*Escalation during SDM*
Autonomy 1989	0.338**		0.557***		0.628***	
	(0.107)		(0.102)		(0.136)	
Autonomy		4.513***		0.621		1.949**
		(0.705)		(1.329)		(0.657)
Most powerful	0.280*	-3.230***				
	(0.132)	(0.895)				
Included	-0.0591	0.213	0.14	0.149	0.250**	-0.131
	(0.0622)	(0.463)	(0.0904)	(0.918)	(0.0965)	(0.571)
Group size	-0.0548	2.584	0.335	-3.058	0.897†	-3.528
	(0.209)	(1.625)	(0.335)	(2.516)	(0.504)	(3.325)
Distance border (log)	0.0197†	-0.112†	0.00826**	0.126***	0.00597†	-523.9
	(0.0112)	(0.0642)	(0.00277)	(0.0204)	(0.00310)	(422.0)
TEK irredentism	-0.0309	0.597†	-0.0525	-1.023†		
	(0.0350)	(0.340)	(0.0650)	(0.522)		
TEK SDMs	0.0477†	-0.334†	-0.0965***	0.0294	-0.123***	0.403
	(0.0287)	(0.202)	(0.0222)	(0.300)	(0.0323)	(0.374)
Petroleum % in settlement area	-0.132**	0.622*	-0.0608**	0.522*	-0.0306	0.67
	(0.0462)	(0.312)	(0.0229)	(0.221)	(0.0199)	(0.410)
GDP p.c. (log)	0.0325	0.0961	-0.0341	-0.574	-0.130*	-0.0159
	(0.0286)	(0.214)	(0.0301)	(0.352)	(0.0561)	(0.337)
Population (log)	0.0693†	-0.387	0.0524**	-0.382*	0.0985†	-0.438**
	(0.0397)	(0.258)	(0.0192)	(0.165)	(0.0514)	(0.141)
Previous SDMs	0.00767	-0.0387	-0.000186	0.0729***	0.004	0.0145
	(0.00486)	(0.0285)	(0.00180)	(0.0188)	(0.00594)	(0.0110)
Normalized polity score	-0.0148	0.11	0.12	0.0905	0.347***	-0.27
	(0.0895)	(0.507)	(0.102)	(1.028)	(0.0932)	(0.321)
Constant	-0.325	-1.736	0.212	3.154	0.899*	-0.819
	(0.233)	(2.028)	(0.195)	(2.215)	(0.451)	(3.004)
Observations	879	879	425	425	939	939
ρ		-1.460***		1.013***		-0.442**
		(0.271)		(0.250)		(0.147)
σ		-1.806***		-1.851***		-1.757***
		(0.0657)		(0.107)		(0.0822)
F (equation 1)		9.7183		29.2690		20.6615
Log pseudolikelihood		277.73151		144.62909		262.63978
Prob > χ^2		0.000		0.000		0.000
Kleibergen-Paap rk LM statistic		4.471		3.817		2.735
Cragg-Donald Wald F statistic		68.741		226.400		379.802
Kleibergen-Paap rk Wald F statistic		9.7183		29.2690		20.6615

$^{***}p < .01$; $^{**}p < .05$; $^{*}p < .1$; Country-clustered errors in parentheses. Cubic term for time dependence of dependent variables included but not reported.

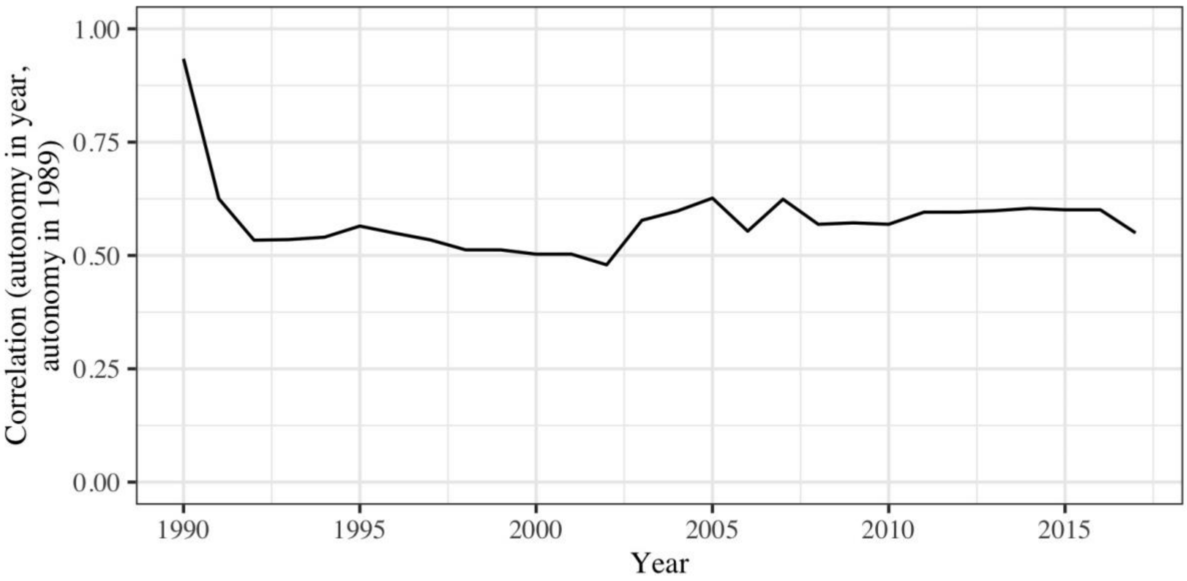

Figure A28. Correlation of group-wise degree of autonomy between 1990 and 2017 with corresponding degree of autonomy in 1989.

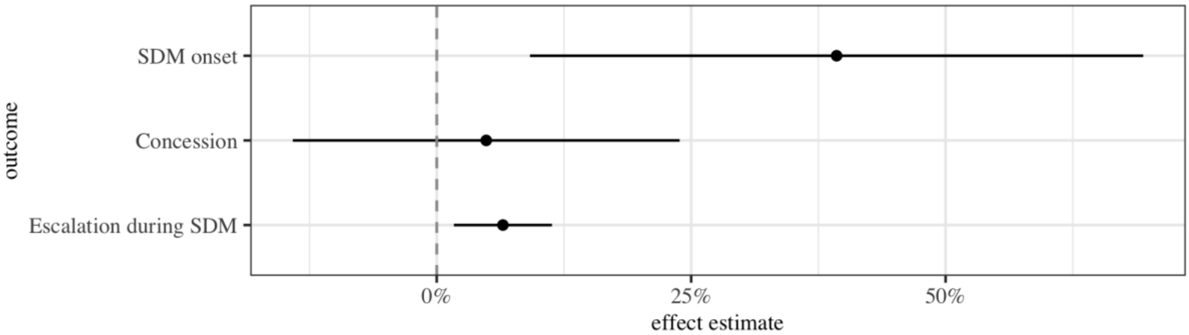

Figure A29. First difference in the predicted probability of SDM onset, concessions during SDM, and escalation during SDM, depending on autonomy in 1989. Based on models 1-3 in table A30.

Appendix E.5: Probing additional assumptions

Table A31. Probing the impact of SDMs on changes in the degree of autonomy in the pre-transition period.

	**Autonomy (t) - Autonomy (t-1)**	
	all Communist countries (CEE and SU)	*Only Soviet Union*
	**Model 1**	**Model 2**
Autonomy (t-1)	-0.033	-0.005***
	(0.024)	(0.002)
Ongoing SDM (t-1)	0.007*	0.001
	(0.004)	(0.003)
Most powerful	0.0004	0.034
	(0.005)	(0.045)
Included	0.023***	
	(0.008)	
Group size	-0.024***	-0.064
	(0.008)	(0.084)
TEK irredentism	-0.008	
	(0.009)	
TEK SDMs	-0.011	-0.002
	(0.007)	(0.002)
Petroleum % in settlement area	0.002	-0.001
	(0.002)	(0.001)
Previous SDMs	0.0001	-0.0003
	(0.0002)	(0.0002)
Normalized polity score	0.004	0.017*
	(0.018)	(0.010)
Distance border (log)	-0.00002	0.00004
	(0.0001)	(0.00004)
GDP p.c. (log)	-0.003	-0.001
	(0.003)	(0.001)
Population (log)	0.001	0.001
	(0.002)	(0.002)
Constant	0.034	0.0004
	(0.030)	(0.018)
N	2396	1622
R-squared	0.045	0.015
Adj. R-squared	0.039	0.008
Residual Std. Error	0.032 (df = 2382)	0.011 (df = 1610)
F Statistic	8.548*** (df = 13; 2382)	2.188** (df = 11; 1610)

***p < .01; **p < .05; *p < .1. Country-clustered errors in parentheses.

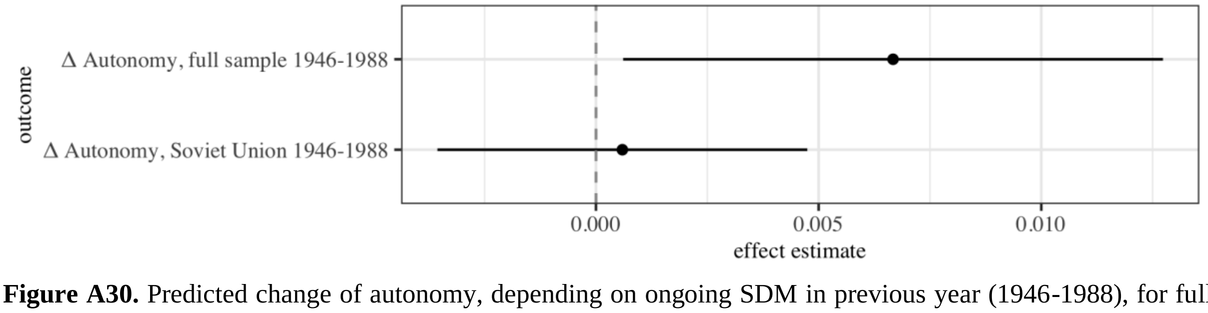

Figure A30. Predicted change of autonomy, depending on ongoing SDM in previous year (1946-1988), for full CEE-SU sample and for sub-sample of Soviet Union only. Based on models 1-2 in table A31.

Table A32. Reverse models.

	**Autonomy (1989)** **Model 1**	**Autonomy (1989)** **Model 2**
SDM onsets after 1989	0.083	
	(0.064)	
SDM escalations after 1989		0.020
		(0.017)
Size in successor state	0.250	0.240
	(0.196)	(0.200)
Petroleum % in settlement area	0.019	0.024
	(0.059)	(0.060)
Distance border successor state (log)	0.0003	0.0002
	(0.003)	(0.003)
Constant	0.205^{***}	0.218^{***}
	(0.058)	(0.054)
N	129	129
R-squared	0.043	0.026
Adj. R-squared	0.012	-0.005
Residual Std. Error (df = 124)	0.246	0.248
F Statistic (df = 4; 124)	1.399	0.840

$^{***}p < .01$; $^{**}p < .05$; $^{*}p < .1$. Country-clustered errors in parentheses.

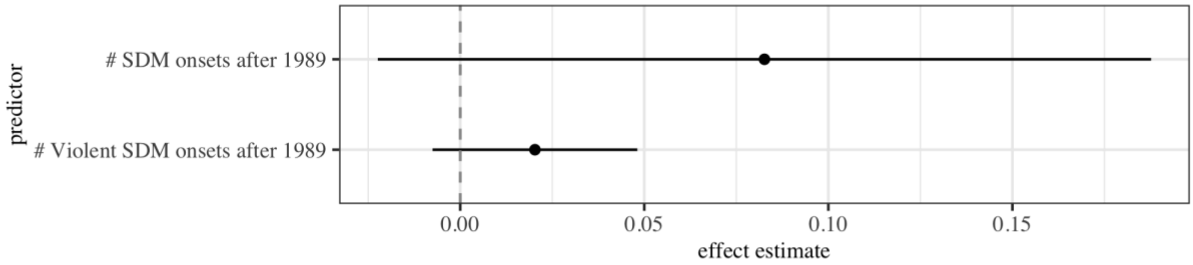

Figure A31. Change in predicted autonomy in 1989, depending on number of (violent) SDM onsets between 1990 and 2017. Based on models 1-2 in table A32.

Additional literature

Cederman, L.-E., Weidmann, N. B., & Gleditsch, K. S. (2011). Horizontal Inequalities and Ethnonationalist Civil War: A Global Comparison. *American Political Science Review*, *105*(3), 478–495.

Cinelli, C., & Hazlett, C. (2020). Making sense of sensitivity: Extending omitted variable bias. *Journal of the Royal Statistical Society: Series B (Statistical Methodology)*, *82*(1), 39–67.

Cohen, F. S. (1997). Proportional versus majoritarian ethnic conflict management in democracies. *Comparative Political Studies*, *30*(5), 607–630.

Deiwiks, C., Cederman, L.-E., & Gleditsch, K. S. (2012). Inequality and conflict in federations. *Journal of Peace Research*, *49*(2), 289–304.

Elkins, Z., Ginsburg, T., & Melton, J. (2014). *The Comparative Constitutions Project: A Cross-national Historical Dataset of Written Constitutions*. www.comparativeconstitutionsproject.org

Klein Goldewijk, K., Beusen, A., Doelman, J., & Stehfest, E. (2017). Anthropogenic land use estimates for the Holocene – HYDE 3.2. *Earth System Science Data, 9*(2), 927–953.

Law, G. (2010). *Administrative Subdivisions of Countries: A Comprehensive World Reference*. McFarland & Company.

Lindberg, S. I., Düpont, N., Higashijima, M., Kavasoglu, Y. B., Marquardt, K. L., Bernhard, M., Döring, H., Hicken, A., Laebens, M., Medzihorsky, J., Neundorf, A., Reuter, O. J., Ruth-Lovell, S. P., Weghorst, K. R., Wiesehomeier, N., Wright, J., Alizada, N., Bederke, P., Gastaldi, L., … Seim, B. (2022). *Varieties of Party Identity and Organization (V-Party) Dataset V2.*

Nordhaus, W. D. (2006). Geography and Macroeconomics: New Data and New Findings. *Proceedings of the National Academcy of Sciences*, *103*(10), 3510–3517.

Timoneda, J. C. (2021). Estimating group fixed effects in panel data with a binary dependent variable: How the LPM outperforms logistic regression in rare events data. *Social Science Research, 93*, 102486.

Vogt, M., Gleditsch, K. S., & Cederman, L.-E. (2021). From Claims to Violence: Signaling, Outbidding, and Escalation in Ethnic Conflict. *Journal of Conflict Resolution*, 002200272199643.
